# Supplementary material for: Comparative genomics reveals a unique nitrogen-carbon balance system in Asteraceae
Source: Nat Commun. 2023 Jul 20;14:4334. doi: 10.1038/s41467-023-40002-9 (PMC10359422; doi:10.1038/s41467-023-40002-9)
Supplement: Supplementary file 1 — Supplementary Information [file 41467_2023_40002_MOESM1_ESM.pdf]

Comparative genomics reveals a unique nitrogen-carbon balance system in Asteraceae

Shen *et al*.

Supplementary Note 1. Background information of Asteraceae

Angiosperm species (flowering plants) experienced rapid terrestrial colonization and diversification, leading to their ecological dominance before the end of the Cretaceous period. Charles Darwin famously characterized the origin of flowers as "an abominable mystery"[1]. In particular, the family Asteraceae (daisies) rivals the Orchidaceae as the most prolific family of flowering plants, comprising more than 1,620 genera and 30,000 species and accounting for ~10% of all flowering species (Supplementary Fig. 1a)[2,3]. Asteraceae represents the most ecologically successful flowering plant family with high diversity and excellent adaptability, as its members occupy nearly every habitat on earth, including extreme environments such as deserts and salt flats (Supplementary Fig. 1b,c)[4]. Another tribute to the extraordinary adaptability of Asteraceae is that some of its members rank among the top three invasive species (Supplementary Fig. 1d).

The ecological success of Asteraceae is thought to stem largely from its specific morphology and physiology. For instance, the characteristic inflorescence (*capitulum*) substantially contributes to ecological radiation by attracting insect pollinators[5], and the achene-like fruits (*cypselae*) with pappus of bristles promote dispersion by wind or attach to the fur or plumage of animals[2]. Seed dispersal is thus enhanced, and Asteraceae seeds can disperse over a greater distance than most other types of seeds. In addition, inulin-type fructans, rather than starches, are the primary reserve carbohydrates in Asteraceae, and they have potential functions in adaptation to adverse environments[6,7]. However, understanding the mechanisms underlying the explosive diversification and adaptability of Asteraceae, especially its genetic basis, remains a considerable challenge to biologists.

Moreover, the origin and early evolution of Asteraceae is inconclusive and mysterious. The family was considered relatively young (40–50 million years ago [MYA]) based on its fossil record, and this time frame is consistent with molecular clock dating studies[2,3,8]. However, recently discovered pollen fossils and phylogenetic studies with broader sampling of the family placed its origin sometime in the late Cretaceous period (69–89 MYA)[2]. In addition, near the crown node of Asteraceae, a paleopolyploidization (whole-genome triplication [WGT]) event was proposed by recent genomic analysis[9]. Frequent potential ancient whole-genome duplications (WGDs) within several tribes have been estimated and predicted to be a driving force in the evolution and increasing biodiversity of the family. Polyploidization events duplicate all genes simultaneously and provide abundant genetic materials for evolutionary processes such as neofunctionalization, subfunctionalization, and gene conservation owing to dosage effects[9,10]. Insights into polyploidization and the underlying genetic basis of specific traits of Asteraceae are now possible by exploiting high-quality genomes.

To solve these puzzles, we generated two high-quality genome assemblies of two species: stem lettuce (*Lactuca sativa* var. *angustana*), a representative economic crop of the Asteraceae family, and *Scaevola taccada*, a representative plant of the Goodeniaceae family that is the sister lineage to Calyceraceae and Asteraceae (Fig. 1a,b). We also performed a comparative genomics analysis of 29 taxa, consisting of 7 Asteraceae species and 22 species representing different evolutionary branches of terrestrial plants.

Supplementary Note 2. Plant materials and genome size estimation of stem lettuce

This study used the stem lettuce cultivar 'YanLing' (YL), which is widely planted in China, for genome sequencing. Multiple rounds of selfing were conducted to reduce the heterozygosity of the genome. Sequencing libraries with insert sizes of 300 bp were then constructed using Illumina TruSeq Nano DNA Library Prep Kits (San Diego, CA). The resulting library was subsequently sequenced using an Illumina HiSeq X Ten instrument. We obtained ~340 Gb of raw sequencing data (Supplementary Table 1). Jellyfish[11] software was used to calculate the *k*-mer frequency (*k*-mer length 21) from the clean reads, and genome heterozygosity, repeat content, and genome size were estimated using Genomescope[12]. The estimated genome size was ~2.54 Gb, slightly smaller than the estimated genome size (2.7 Gb) reported previously for the *La. sativa* cultivar 'Salinas' but close to the genome size estimated by flow cytometry (Supplementary Fig. 2)[13].

Supplementary Note 3. Genome and transcriptome sequencing of stem lettuce

After the 300-bp DNA library of stem lettuce was constructed, paired-end short reads were generated on an Illumina platform as described previously, reaching a fold coverage of 170× based on the estimated genome size of ~2.54 Gb (reaching 426.7 Gb of reads) (Supplementary Table 1). Other 20-kb libraries were constructed using SMRTbell Template Prep Kits (Pacific Biosciences, Menlo Park, CA) and subsequently sequenced via a PacBio RS II instrument (Pacific Biosciences) with the P6-C4 sequencing reagent. A fold genome coverage of ~112× was produced with PacBio single-molecule long reads (284.73 Gb with an N50 length of 19.8 kb) (Supplementary Table 1).

The protocol for the Hi-C library, including cellular cross-linking, chromatin digestion, labeling of DNA ends, DNA ligation, purification, and fragmentation, followed the standard technique[14]. Nuclear DNA from young leaves was cross-linked *in situ*, extracted, and digested with a restriction enzyme (MobII). The sticky ends of the digested fragments were biotinylated, diluted, and then ligated randomly. The biotinylated DNA fragments were enriched and sheared again to generate a sequencing library, which was subsequently sequenced on an Illumina HiSeq X Ten instrument, resulting in an ~117× coverage of Hi-C data (292.8 Gb) (Supplementary Table 1).

To achieve a high-quality reference genome, we also employed BioNano optical mapping technology. Since long DNA fragments are required as input, genomic DNA was extracted from fresh young YL leaves and embedded in a thin agarose layer for labeling at Nt.BspQI sites using the IrysPrep Reagent Kit protocol (BioNano Genomics, San Diego, CA) and subjected to optical scanning on the BioNano Irys platform (BioNano Genomics). This method produced an ~224× coverage optical map data (320 Gb with an N50 length of ~280 kb) (Supplementary Table 2).

Transcriptome sequencing was performed to obtain transcripts for gene model prediction. Following the manufacturer's instructions, samples from flowers, leaves, stems, and roots were collected and processed for library construction. The quality and quantity of the RNA samples were

evaluated using a NanoDrop D-1000 spectrophotometer (NanoDrop Technologies, Wilmington, DE), a Qubit® 3.0 Fluorometer (Thermo Fisher Scientific, USA), and an Agilent Bioanalyzer 2100 (Agilent Technologies, CA, USA). Paired-end libraries with insert sizes of 300 bp were constructed using a TruSeq Sample Preparation kit and sequenced with an Illumina HiSeq X Ten platform, generating ~25.6 Gb of sequencing data (Supplementary Table 1).

Supplementary Note 4. Genome assembly of stem lettuce

First, we used Falcon (version 0.4)[15] to construct the initial contigs. The initial polishing was then performed with Arrow (version 2.2.3) using PacBio-only long reads, after which Pilon (version 1.20)[16] was used to correct the contigs with Illumina short reads. The assembly was performed stepwise, and the initial assembly of the PacBio-only data generated a 2.60-Gb genome size comprising 1,959 contigs with a contig N50 of 4.95 Mb (Supplementary Tables 2 and 3).

The BioNano data were first assembled into a consensus map using the IrysView software (BioNano Genomics; version 2.5.1) with a molecular length threshold of 150 kb and a minimum label number per molecule of 8. Hybrid scaffolding of the PacBio-corrected contigs and BioNano-based consensus map was performed through the hybrid scaffolding module within the IrysView software (version 2.5.1) with the parameters suggested by the manufacturer. After filtering, we obtained 909,232 molecules with a total length of 267,783 Mb and an N50 of 315 kb, corresponding to nearly 104× coverage of the genome. Of these molecules, 735,574 could be aligned to the assembled contigs. We conducted *de novo* assembly using the obtained molecules and generated a genome map length of 2.56 Gb with 36 scaffolds and an N50 of 185.63 Mb. Hybrid scaffolding of the PacBio-corrected contigs and BioNano-based consensus map generated a 2.59-Gb assembly with 27 superscaffolds and a scaffold N50 of 186 Mb (Supplementary Tables 2 and 3). There were 955 initial contigs with an N50 of 4.75 Mb, covering 2.56 Gb in superscaffolds. To check the correctness of the superscaffolds, we aligned the superscaffolds to the Lsa_v1 genome[13] from lettuce cultivar 'Salinas' using MUMmer4.0[17] software, and the resulting showed that the superscaffolds are in consensus with the previously reported lettuce genome, which was assembled into pseudo-chromosomes using a high-quality linkage map.

We also tried to cluster the superscaffolds and remaining contigs into chromosomes using Hi-C sequencing data. The Hi-C sequencing data were first pre-processed and aligned to the assembled genome using the Juicer platform (version 1.5)[18]. The assembled scaffolds were then clustered into pseudo-chromosomes (superscaffolds) using the 3D *de novo* assembly pipeline[14]. Finally, scaffolding with Hi-C data allowed the accurate clustering and ordering of nine pseudochromosomes covering the 2.59-Gb assembly, with a contig N50 of 332.3 Mb and a maximum contig length of 397.9 Mb (Supplementary Tables 2 and 3; Supplementary Fig. 3).

We obtained the complete (circular) organellar genomes using GetOrganelle software[19] and PMAT software (https://github.com/bichangwei/PMAT). Annotation was performed with GeSeq[20] using default parameters to predict protein-coding genes, transfer RNA (tRNA) genes, and ribosomal RNA (rRNA) genes. Manual annotation was performed for genes with low sequence identity to determine the positions of their start and stop codons depending on the translated amino

acid sequence using the chloroplast/bacterial genetic code. OrganellarGenomeDRAW (OGDRAW)[21] was optimized to create detailed, high-quality gene maps of organellar genomes (plastid and mitochondria) (Supplementary Figs. 4 and 5). The total length of the chloroplast genome was 152,765 bp. A total of 88 protein-coding genes, 36 tRNAs and 8 rRNAs were annotated. The total length of the mitochondria genome was 363,326 bp with 41 protein-coding genes, 27 tRNAs and 6 rRNAs annotated (Supplementary Figs. 4 and 5). The sequences of organellar genomes and their annotation have been uploaded into Github (https://github.com/maypoleflyn/lettuce_data).

Supplementary Note 5. Genome annotation of stem lettuce

Protein-coding genes were predicted from the repeat-masked SL1.0 assembly with the MAKER-P program (v2.31.10)[22], which integrates evidence from protein homology, transcripts, and *ab initio* predictions. The homology-based evidence was derived by aligning protein sequences from seven plant species to the SL1.0 assembly: Arabidopsis, lettuce (*La. sativa*), sunflower (*Helianthus annuus*), cardoon (*Cynara cardunculus*), chrysanthemum (*Chrysanthemum nankingense*), sweet wormwood (*Artemisia annua*), and rice (*O. sativa*).

RNA-seq data derived from four different libraries was *de novo* assembled using Trinity (v2.8.2) software[23]. We extracted 2,033 mRNAs and 326,941 ESTs from the NCBI nucleotide database (https://www.ncbi.nlm.nih.gov/nucleotide/), which were also used for gene prediction. First, all transcript sequences were uploaded to the PASA pipeline[23] to conduct the alignment assembly. Five thousand complete gene models and sequences were extracted to train the parameters for SNAP and Augustus software[22]. All data and predictions were then used to produce a consensus gene set. Finally, the PASA pipeline was run again to refine the obtained gene models.

We identified 40,341 high-confidence protein-coding genes. The annotated genes covered 95.9% of the complete BUSCO genes, and ~86.68% of the annotated genes were expressed in at least one tissue or were homologous to known protein-coding genes, which suggested that our gene annotation was of high quality (Supplementary Table 4).

Gene functions were annotated using InterProScan (v5.24)[24] by searching against publicly available databases, including ProDom[25], PRINTS[26], Pfam[27], SMART[28], PANTHER[29], and PROSITE[30]. The Gene Ontology (GO) and Kyoto Encyclopedia of Genes and Genomes (KEGG) IDs for each gene were assigned according to the corresponding InterPro entry. Homology of SL1.0 proteins was determined by pairwise sequence comparison using the BLASTP algorithm against Swiss-Prot and TrEMBL databases (with a threshold of E-value $\leq$ 1e−5). A total of 34,462 genes were annotated using InterProScan; 21,094 genes and 7,553 genes were successfully assigned to GO and KEGG IDs, respectively, and 26,895 genes had homologous genes in the UniProt databases[31] (Supplementary Table 4).

Noncoding RNAs (including rRNA, tRNA, small noncoding RNA [snRNA], small nucleolar RNA [snoRNA], and microRNA [miRNA]) play important roles in cellular processes such as DNA replication, RNA transcription and processing, protein transport, and degradation. We conducted an

analysis of noncoding RNAs using rfam_scan.pl and the Rfam (version 11) database[32], which includes 383,004 sequences and 2,208 Rfam families. In total, we obtained 5,453 noncoding RNAs, consisting of 2,080 rRNAs, 2,255 tRNAs, 613 snRNAs, 90 signal recognition peptide (SRP) RNAs, and 278 snoRNAs (Supplementary Table 5).

To refine the identification of miRNAs, we constructed nine small RNA libraries using nine samples collected from roots, stems, and leaves (as three biological replicates). Reads were aligned to the repeat-masked genome using BWA software[33], and miRNAs were identified by miRDeep-P2[34], resulting in the identification of 137 high-confidence miRNAs in SL1.0 (Supplementary Table 5).

A customized repeat library was built to include known and novel repeat families. We first searched the assembly for miniature inverted transposable elements (MITEs) using MITE-Hunter[35] with default parameters. LTR_retriever pipeline[36] was then applied to integrate the candidate LTR-RTs identified by LTR_FINDER[37] and LTRharvest[38]. We obtained an LRT-RT library containing 36,018 intact LTR-RTs. An initial repeat masking of the SL1.0 assembly was performed with the repeat library derived by combining the identified MITEs and LTR-RTs. The repeat-masked genome was fed to RepeatModeler (version 1.0.11) to identify novel repeat families. Finally, all identified repeat sequences were combined and searched against a plant protein database from which transposon-encoding proteins were excluded. Elements with significant similarity to plant genes were removed. We employed RepeatMasker (version 4.0.6) to search for similar transposable elements (TEs) with the known Repbase TE library and customized repeat library.

Detailed annotation of the repeat sequence in the stem lettuce assembly revealed that ~2.28 Gb of the genome regions (or ~87.75% of the genome assembly) are occupied by different kinds of repeat sequences, which was consistent with *k*-mer frequency analysis but higher than that (74%) of the previously reported lettuce assembly. Except for about 1% of short simple repeat sequences, TEs represent ~2.27 Gb (or 87.64%) of the 2,597.9-Mb genome assembly. The most abundant repeats in this genome were retrotransposons or class I elements (91.67% of TE content, 80.43% of genome assembly), and in particular LTR-RTs, which represent up to 99.4% of this type of repeat, whereas non-LTR-RTs (long and short interspersed nuclear elements) accounted for the remaining 0.6%. DNA transposons or class II elements (DNA transposons and *Helitron*s) made up 1.67% of the TE content (1.46% of the genome assembly) (Supplementary Table 10).

Tandem repeat elements were enriched in the centromeric region. However, based on existing approaches, we could not detect significantly enhanced tandem centromeric repeat elements in SL1.0. We investigated the distribution of repeat sequences across the genome using a sliding window strategy (Supplementary Fig. 6). The percentage of repeat sequences across the genome reached a high level for all pseudochromosomes. Repeat sequences always occupy the vast majority of heterochromatin regions. Based on the profiles, some chromosomes (e.g., Chr02, Chr06, and Chr07) showed clear peaks in their center, suggesting the location of the heterochromatin region and centromeres. However, for other chromosomes, the location of centromeres was probably concealed by a complicated karyotype (e.g., acrocentric chromosomes). The complete genome sequence in this study could provide valuable references for karyotype studies of the Asteraceae

family (Supplementary Fig. 6).

Supplementary Note 6. Genome assessment of stem lettuce genome

We assessed the genome quality at different levels. We aligned all Illumina reads to the assembled YL genome, namely, SL1.0, using BWA software[33]. Of all reads, 1,932,299,337 (or 98.37%) of them properly aligned to the SL1.0 assembly (Supplementary Table 4). Furthermore, we estimated the base accuracy of the sequencing by calculating the quality value (QV) of the assembly as described for the gorilla genome assembly[39]. We conducted single-nucleotide polymorphism (SNP) calling based on the aligned reads using SAMtools software[40], and we counted the number of SNPs with a Phred-scaled greater than 30 and coverage of at least 3 (n). We also calculated the number of genome positions with read coverage greater than 3 (N). Finally, we determined the QV of the assembly using the following equation[41]:

$$QV = \mathrm{Log}_{10}(n/N) \qquad \textbf{(1)}$$

QV represents the limit of the base accuracy of the assembly. In this study, QV of the assembly was at least 42.38, which compared very favorably to the QVs of two published mammalian genomes (QV = 35 for gorilla and QV = 34.5 for goat)[39,41] that were also assembled with PacBio data.

Second, we estimated the completeness of gene annotations using the expression data. The 81,330 expressed sequence tags (ESTs) from GenBank were aligned to the SL1.0 assembly using the BLAST-like alignment tool (v0.36) with default parameters[42], and 95.32% of ESTs could be aligned to the assembly (Supplementary Table 7, Supplementary Table 8). A total of 108,367,024 reads from different tissues (roots, leaves, flowers, and stems) were generated and aligned to SL1.0, of which 97.6% mapped successfully (Supplementary Table 8). Based on a benchmark of 1,440 conserved plant genes, ~95.0% complete Benchmarking Universal Single-Copy Ortholog (BUSCO) genes could be detected in the assembly (Supplementary Table 9; Supplementary Fig. 7). Moreover, our assembly captured five long stretches of telomeric sequences (5-TTTAGGG-3) at both ends of five chromosomes, with repeat numbers ranging from 294 to 1,073 (Supplementary Table 11). These observations confirmed the high completeness of the assembly.

Furthermore, we also estimated the structural accuracy of the assembly. We determined that ~98.37% of the mapped Illumina reads of YL could be mapped in the correct orientation and correct estimated insert size (Supplementary Table 6). We conducted pairwise alignments between the SL1.0 and the Salinas (Lsa_v1) genome using Minimap2[43] software and visualized the result using Minidot software (https://github.com/thackl/minidot). The whole-genome alignment of YL and Lsa_v1 showed strong co-linearity and consistency (Supplementary Fig. 8)[13].

Genome assembly quality was assessed by the LTR Assembly Index (LAI)[44]. Assembling a plant genome is challenging due to the abundance of repetitive sequences, and long terminal repeat (LTR) retrotransposons (RTs) are the predominant form of interspersed repeats in these genomes. We evaluated the assembly of the repeat space with the LAI program[44], which assesses assembly

continuity using LTR-RTs. The LTR_retriever pipeline was first conducted to integrate the candidate LTR-RTs identified by LTR_FINDER (version 1.0.6)[37] and LTRharvest (version 1.5.9)[38]. Then, whole-genome LAI was calculated based on the LTR-RT library generated by LTR_retriever (version 2.9)[36]. The LAI of SL1.0 was up to 18.13, which was comparable to those of the rice (*Oryza sativa*), maize (*Zea mays*), and Arabidopsis (*Arabidopsis thaliana*) genomes[44] (Supplementary Fig. 9).

Supplementary Note 7. Plant materials and genome size estimation of *Sc. taccada*

We collected *Sc. taccada* plants from Hainan, a southern province of China. Two methods were employed to estimate the genome size of *Sc. taccada*: i) flow cytometry compared to reference plant genomes and ii) *k*-mer-based estimation. In the flow cytometry estimation, we compared the genome size of *Sc. taccada* to that of black cottonwood (*Populus trichocarpa*) (2n = 2x = 38) and tomato (*Solanum lycopersicum*) (2n = 2x = 24). The genome size of *Sc. taccada* was ~1 Gb (Supplementary Fig. 10). To estimate the *Sc. taccada* genome size by the *k*-mer method, genomic DNA was first extracted from leaves of seedlings using the phenol–chloroform method[45], and a library with insert sizes of 300 bp was constructed using Illumina TruSeq Nano DNA Library Prep Kits followed by sequencing via an Illumina HiSeq X Ten instrument. The resulting estimated genome size was ~1,045 Mb, which was similar to the result from the flow cytometry method, with a heterozygosity of 0.643% (Supplementary Fig. 10).

Supplementary Note 8. Genome and transcriptome sequencing of *Sc. taccada*

As with stem lettuce, paired-end short reads from the 300-bp DNA library were sequenced on an Illumina platform to ~105× coverage (or 115 Gb) (Supplementary Table 12). Similarly, ~102× coverage (~111 Gb) with an N50 length of 25.4 kb was obtained from PacBio reads via a PacBio RS II instrument (Pacific Biosciences) with the P6-C4 sequencing reagent. We also constructed a Hi-C library according to the method described for stem lettuce. We obtained ~102× coverage (~112 Gb) of Hi-C short reads (Supplementary Table 12). RNA-seq libraries were constructed from flower, leaf, and stem samples, resulting in ~26.4 Gb data, with 6.6 Gb on average for each RNA-seq sample (Supplementary Table 12).

Supplementary Note 9. Genome assembly and evaluation of *Sc. taccada*

First, we corrected the PacBio long reads using the reads correction module of the CANU pipeline[46], retaining ~36× coverage (~36 Gb) with the longest corrected PacBio reads. Second, we conducted a *de novo* assembly using WTDBG2 software[47] and the corrected PacBio reads in CCS mode. Together, we obtained a genome assembly, ST1.0, with a genome size of 1,159 Mb, with a contig N50 of ~9,636 kb (Supplementary Tables 13,14). We also clustered the superscaffolds and remaining contigs into pseudochromosomes using Hi-C sequencing data. The Hi-C sequencing data were first pre-processed and aligned to the assembled genome using the Juicer platform (version

1.5)[18]. The assembled scaffolds were then clustered into pseudochromosomes (superscaffolds) using the 3D *de novo* assembly pipeline[14] (Supplementary Fig. 11). We obtained the complete (circular) organellar genomes using a method similar to that used for stem lettuce (Supplementary Figs. 12 and 13). The total length of the chloroplast genome was 182,015 bp. A total of 86 protein-coding genes, 36 tRNAs and 8 rRNAs were annotated. The total length of the mitochondria genome was 314,162 bp with 43 protein-coding genes,19 tRNAs and 8 rRNAs annotated (Supplementary Figs. 12 and 13). The sequences of organellar genomes and their annotation have been uploaded into Github (https://github.com/maypoleflyn/lettuce_data). The completeness of the genome was estimated using BUSCO scores: The *Sc. taccada* genome contains ~94.2% complete BUSCOs (Supplementary Table 18; Supplementary Fig. 14).

Supplementary Note 10. Genome annotation of *Sc. taccada*

We collected samples from three tissues (roots, leaves, and flowers), performed RNA-seq, and conducted *de novo* assembly using Trinity software[23]. We obtained 147,271 transcripts from 45,901,662 RNA-seq reads, which were then used as an EST library for the Maker-P[22] genome annotation pipeline. We also collected the predicted proteins from seven Asteraceae species and all protein sequences encoded by BUSCO genes as the homologous protein library. After processing of the genome annotation by the Maker-P pipeline, 25,328 protein-coding genes were obtained (Supplementary Table 16).

We also conducted an annotation of noncoding RNAs using the Rfam (version 11)[32] library, which returned 4,219 noncoding RNAs (Supplementary Table 17).

We constructed a species-specific repeat sequence library by the same method used for *Sc. taccada*. For the LTR repeat sequences, we detected 5,487 intact LTR elements in the genome, and a redundant LTR library of 16,949 elements was clustered into 2,748 representative sequences. We annotated the repeat sequences using the species-specific repeat sequence library and RepeatMasker software. Nearly 80.69% (~66.32% RTs and ~3.51% DNA transposons) of the genome was annotated as repeat sequences (Supplementary Table 15).

Supplementary Note 11. Phylogenomic analysis

In addition to 7 sequenced Asteraceae genomes and the *Sc. taccada* assembly, 21 representatives of different phylogenetic branches of land plants were selected. These included another seven representative species in Asterids: golden kiwi (*Actinidia chinensis*), carrot (*Daucus carota*), potato (*Solanum tuberosum*), tomato (*S. lycopersicum*), Arabic coffee (*Coffea arabica*), olive (*Olea europaea*), and monkeyflower (*Mimulus guttatus*). Six species from the Rosids clade were also selected: wild strawberry (*Fragaria vesca*), Arabidopsis, field mustard (*Brassica rapa*), sweet orange (*Citrus sinensis*), black cottonwood (*P. trichocarpa*), and grapevine (*Vitis vinifera*). Finally, three monocot species and five ancient species were included: pineapple (*Ananas comosus*), rice (*O. sativa*), common duckmeat (*Spirodela polyrhiza*), gingko (*Ginkgo biloba*), Norway spruce (*Picea

abies), *Selaginella moellendorffii*, *Amborella trichopoda*, and *Liriodendron chinense*. Using these 29 species, accurate inference of the orthologous groups was achieved with OrthoFinder (v2.4.0)[48]. Low-copy number (LCN) genes were identified based on OrthoFinder results with the following requirements: strictly single-copy genes in *La. sativa*, *Sc. taccada*, *Se. moellendorffii*, *G. biloba*, and *V. vinifera*, and at least 5 of the other 24 selected species. We thus identified 389 orthogroups (OGs), including 9,784 orthologous LCN genes.

We adopted two independent methods to reconstruct the species tree of the selected species. Multiple alignments were conducted using MUSCLE (v3.8.1551)[49] software. Next, trimAL[50] was used to trim low-quality aligned regions with the option "-automated1". LCN gene trees were estimated from the remaining sites using RAxML (v.7.7.8)[51] using the JTT+G+I model for amino acid sequences. Phylogenetic reconstruction was performed stepwise with 9,784 carefully selected sets using the coalescence method implemented in ASTRAL (v5.5.1)[52]. Also, we concatenated multiple alignment sequences of the genes in the 389 OGs and reconstructed species trees using RAxML (v.7.7.8). The topology of species trees of the two methods was consistent (Supplementary Figs. 15 and 16).

To estimate the evolutionary timescale of species, we concatenated 389 LCN genes (185,822 sites) in each species and fixed the tree topology inferred from our coalescent-based analysis of 9,784 genes from 29 taxa. We performed a Bayesian phylogenomic dating analysis of the selected genes in MCMCtree, part of the PAML package[53], and an approximate likelihood calculation for the branch lengths. Molecular dating was performed using an auto-correlated model of among-lineage rate variation, the JC69 substitution model, and a uniform prior on the relative node times. Posterior distributions of node ages were estimated using Markov chain Monte Carlo sampling, with samples drawn every 200 steps over 10 million steps following a burn-in of 200,000 steps. We also implemented the penalized likelihood method under a variable substitution rate using r8s[54]. Our penalized likelihood dating analysis implemented three fossil calibrations (corresponding to crown groups of angiosperms, eudicots, and monocots) as minimum age constraints. We determined the best smoothing parameter value of the concatenated LCN genes by performing cross-validations of a range of smooth parameters from 0.01 to 10,000 (algorithm = TN; crossv = yes; cvstart = 0; cvinc = 0.5; cvnum = 15). Finally, we calibrated a relaxed molecular clock using pairwise divergence time on the TIMETREE[55] website (http://www.timetree.org/) and the estimated species divergence time from the r8s software.

Multiple models with three fossil calibrations corresponding to the crown groups of angiosperms, eudicots, and monocots were performed to determine their molecular dating. The lineage differentiation time of *Sc. taccada* and Asteraceae could be traced back to ~78–82 MYA, which was the late Cretaceous period (Fig. 2a), similar to the recently revealed pollen grain fossil record[5] and molecular analytical results based on dispersed genomic and transcriptomic data[2,10].

Supplementary Note 12. WGD analysis

Synteny comparisons were identified by MCscan[56] with default parameters to predict paralogs and

orthologs. Orthologous genes were identified between *Sc. taccada* and lettuce and between artichoke (*C. cardunculus* var. *scolymus*) and coffee (*C. arabica*), as well as paralogous genes among the *Sc. taccada*, lettuce, artichoke, coffee, sunflower (*H. annuus*), and *C. nankingense* genomes. The *Ks* distribution of the paralogs clearly illustrated different rounds of polyploidization events, especially the WGTs experienced by the Asteraceae species, including WGT-γ, WGT-1, and WGT-2 (Fig. 2b)[2,57]. *Sc. taccada* shared a prominent peak with coffee (Fig. 2b), suggesting that *Sc. taccada*, similar to coffee, experienced only the WGT-γ event, a more ancient WGT event from ~122–164 MYA[58]. The similar distribution profiles of *Ks* paralogs between *Sc. taccada* versus lettuce and between lettuce versus lettuce indicated that the WGT event occurred closely after the cladogenesis between Asteraceae and Goodeniaceae (Fig. 2b). WGT-1 was the only WGT event that happened in the ancestors of Asteraceae after WGT-γ. The intergenomic comparison and synteny analyses also supported this observation (Fig. 2b,c; Supplementary Fig. 17). In addition, these analyses suggest that the WGT-1 event could have occurred near the Asteraceae formation time.

Supplementary Note 13. Analysis of triplication-retained regions

To further evaluate the influence of the WGT-1 event on the evolutionary processes of genomes and the potential genetic basis for the formation of traits in Asteraceae species, the *Sc. taccada* genome was utilized as the reference and the triplication-retained regions (TRRs) after the WGT-1 event were investigated (Supplementary Fig. 18). Considering that colinearity analysis requires high-quality genomes, the following four Asteraceae species were kept: lettuce, sunflower, artichoke, and bitter vine (*Mikania micrantha*). A total of 2,116 TRR genes (an average of 529 per genome) were identified in all the 3,884 synteny blocks of the four species versus *Sc. taccada* (Supplementary Table 20), and an average of 1,304 genes were distributed in the TRRs for the surveyed species (Supplementary Table 20). Key homologous genes determining essential biological processes in the TRRs (e.g., flowering, cell wall biosynthesis/metabolism, and fatty acid biosynthesis) were detected (Supplementary Data 1–5). Further analysis of enriched genes in these TRRs revealed that four genes related to the cell wall, protein phosphorylation, fatty acid, and cell membrane were potentially selectively retained (Supplementary Fig. 17b; Supplementary Data 1). These functions are closely related to stress response and environmental adaptation; for example, pectin methylesterase (PME) is the first enzyme acting on pectin, a significant component of the plant cell wall, and has a vital role in stress responses[59]. The main sub-group gene, *PME-1*, was strongly selected in the TRRs (Supplementary Figs. 17d, 18-20). Gene families such as delta-9 acyl-lipid desaturase (*ADS*), *FRUITFULL* (*FUL*), and *MIKC-MADS* showed a similar selection pattern (Supplementary Figs. 17d and18). The proportion of gene regions in the TRRs was significantly higher than that of the whole genome (Supplementary Fig. 17c); correspondingly, the level of repetitive sequences was considerably lower (Supplementary Fig. 17c), indicating that these regions have been favorably selected. In addition, an enrichment and depletion analysis of the repeat element families in these TRRs suggested that the decrease in repeat elements was primarily caused by the deletion of LTR-RTs (Supplementary Fig. 21).

Supplementary Note 14. LTR analysis

The genome sizes of Asteraceae species are generally large and usually range in Gb size[60]. Detailed repetitive element annotations of the 29 representative genomes were used to obtain a better understanding of the genome architecture of Asteraceae. The contents of LTR-RTs were positively correlated with genome size ($R^2 = 0.74$, $P < 0.05$), suggesting that the LTR-RTs could be one of the drivers of genome size expansion, particularly for the species with large genomes (Supplementary Fig. 22a; Supplementary Data 6). The contents of LTR-RTs in the Asteraceae family were higher than those of species with the same genome size level (Supplementary Fig. 22a; Supplementary Data 6), even for species such as artichoke that have a smaller genome. Most of the analyzed Asteraceae species possess LTR-RTs comprising more than 50% of the genome (Supplementary Data 6). These observations indicate that the large proportion of LTR-RTs is a critical reason for the expansion of Asteraceae genome size.

Systematic comparisons between Asteraceae species and five other selected species were performed to uncover the dynamics and roles of LTR-RTs in genome evolution in more detail. The solo/intact ratio of LTR families in the Asteraceae species was predominantly lower than that in the other species, suggesting that a more dynamic mechanism exists to remove abundant LTR-RTs in Asteraceae (Supplementary Fig. 22b). The estimated insertion time of LTR-RTs (intact and truncated) indicated that recent and frequent bursts of LTR-RTs occurred across the evolution of different Asteraceae species (Supplementary Figs. 22c, d, and 23). Specific high-copy LTR-RT families dominate the genomes and expand in distinct modes and rates (Supplementary Fig. 24). In particular, the amplification of *Tekay* of *Ty3*/*Gypsy* comprised the vast majority (~96%) of *Ty3*/*Gypsy* full-length LTR-RTs in the lettuce genome. *Tekay* was present at a higher proportion than in other species, including the related species *Taraxacum kok-saghyz* Rodin, suggesting its rapid amplification in lettuce (Supplementary Figs. 25-28). Moreover, repeated bursts of the *Ivana* and *Tork* lineages predominantly originated from 8 of the top 12 families, resulting in 94% of the full-length *Ty1*/*Copia* LTR-RTs (Supplementary Figs. 22e, 27 and 28). The amplification of different lineages of *Ty3*/*Gypsy* and *Ty1*/*Copia* LTR-RTs was markedly differentiated among the Asteraceae species but related to phylogeny (Supplementary Figs. 27 and 28). For example, *A. annua* and *C. nankingense* harbored a higher proportion of *Athila* and *Reina* of *Ty3*/*Gypsy* LTR-RTs, while *Tekay* of *Ty3*/*Gypsy* LTR-RTs was in the majority in the genomes of sunflower (~79%) and *M. micrantha* (~89%) (Supplementary Fig. 28). Staton and Burke (2015) describe patterns of TE evolution in 14 species in the Asteraceae family using whole-genome shotgun sequencing and found that the TE-driven expansion of plant genomes can be facilitated by just a few TE families[61], which was also supported by our observations.

Active repetitive elements, including (retro)transposons, could theoretically influence the biological functions of genes by altering the gene structure, *cis*-acting elements, or epigenetic state[62]. Enrichment analysis was performed based on the functional domains of all genes across the surveyed 29 species. A total of 578 enriched InterPro entries were detected in the Asteraceae group ($P < 0.05$). A substantial number of domains associated with (retro)transposons were enriched in the proteomes of the Asteraceae species (Supplementary Figs 20f; Supplementary Data 7) (e.g., the reverse transcriptase domain [IPR000477], retrotransposon gag domain [IPR005162], and retrotransposon *Copia*-like domain [IPR029472] of retrotransposons, and the transposase-

associated domain [IPR029480] of transposons), suggesting that the domains associated with (retro)transposases were potentially introduced into numerous genes, possibly causing them to acquire new features or change their original function. For example, a zinc finger gene family acquired the reverse transcriptase domain (IPR000477), experienced rapid gene duplications, and finally formed an Asteraceae lineage-specific family (Supplementary Fig. 20g). Using the *Sc. taccada* genome as a reference, we determined that the host genes of (retro)transposons domains were possibly involved with several vital biological processes (Supplementary Data 8; Supplementary Fig. 29). Taking bromodomain-containing protein 4 (BDR4) as an example, which functions by binding to the acetylated lysine residues present within histone tails, controls gene transcription, and plays an essential role in plant development[63], we observed that a homologous gene of *BDR4* captured a domain from plant transposases, the Ptta/En/Spm families, and resulted in the birth of a unique branch of BDR4 in Asteraceae (Supplementary Fig. 22h, i; Supplementary Data 8).

After carefully screening the selected Asteraceae genomes, we found that transcription factor binding sites (TFBSs) introduced by repetitive elements affect 2.23–3.89% of all genes across these species (Supplementary Data 9-10; Supplementary Table 21; Supplementary Fig. 30). The introduced TFBSs could potentially influence gene expression; for example, a *Copia* LTR-RT introduced the TFBS of *SUPPRESSOR OF OVEREXPRESSION OF CO1* (*SOC1*) and enhanced the expression of *LsNRG*s (*Nitrate Regulatory Gene*) in lettuce (Supplementary Fig. 22j, k). A high occurrence of introduced TFBSs, such as with *SOC1* and *DOF ZINC FINGER PROTEIN4.7* (*DOF4.7*), exhibited a high degree of consistency across the Asteraceae species and was closely related to critical biological processes, such as flowering and cell morphogenesis (Supplementary Table 21; Supplementary Fig. 31), despite the functional diversification and diversity of the host genes (Supplementary Figs. 32–35).

Supplementary Note 15. Orthologous gene analysis

We performed an accurate inference of OGs using OrthoFinder software. All 1,082,770 genes were divided into 28,435 OGs, corresponding to 80.6% of all genes. All seven species in the Asteraceae family shared 8,331 OGs (Supplementary Fig. 36). Based on the phylogenetic tree and orthologous groups, we conducted a computational analysis of gene family evolution using CAFÉ software[65]. We identified 2,868 (2,356 expansions and 512 contractions) significantly changed orthologous groups in the ancestral node of the Asteraceae family and 108 rapidly evolving families. In the ancestral node of the Asteraceae family and Goodeniaceae, we identified 1,869 significantly changed orthologous groups (872 expansions and 997 contractions) and 47 rapidly changing families (Supplementary Data 3). The rapidly evolving gene families are involved in many biological processes, particularly reproduction, response to stimuli, and immune and nutrient reservoirs (Supplementary Fig. 37).

Supplementary Note 16. Functional domain enrichment analysis of gene families

We performed an enrichment analysis using a one-tailed Fisher's test based on the functional domains encoded by all genes across the 29 species. We obtained 578 enriched InterPro entries in the Asteraceae group ($P < 0.05$), of which 107 exhibited enrichment in at least two Asteraceae species ($P < 0.05$). We explored the InterPro entries and identified transposon/retrotransposon-related domains, zinc finger domains, histone domains, and critical functional domains present in proteins encoded by crucial genes in the metabolism of fatty acid biosynthesis (Supplementary Data 11).

1) Among the enriched 107 entries, 18 entries were related to RT or transposon domains (Supplementary Data 11; Supplementary Fig. 22f) such as reverse transcriptase, transcriptase, integrase zinc-binding, and retrotransposon gag domains. We propose that these genes were derived from RTs/transposons or from an insertion of a RT/transposon in the gene structure. We attribute the high percentage of genes harboring RTs/transposons to the high level of repeat sequences in Asteraceae.

2) We also identified several zinc finger domains, including zinc finger, LIM-type, CCHC-type, BED-type, MIZ-type, ZPR1-type, zinc finger-XS domain, LSD1-type, PMZ-type, TTF-type, SWIM-type, and GRF-type domains (Supplementary Data 11). The zinc finger structure is a universal transcription factor structure that recognizes specific nucleotide sequences. However, the exact functions of most proteins containing a zinc finger structure and whether they have other features are unclear. We propose that the existence of enriched proteins could play an essential role in transcriptional regulation or serve a more sophisticated regulatory role.

3) We identified critical functional domains present in proteins encoded by crucial genes in the metabolism of fatty acid biosynthesis, including Acyl-CoA desaturase (IPR015876), β-ketoacyl synthase (IPR014031, IPR014030), and fatty acid desaturase (IPR005804, IPR021863) (Supplementary Data 11). We validated the corresponding genes based on sequence similarity and structural domain characteristics. We found that the number of fatty acid desaturase genes in the Asteraceae group was significantly higher than that of other species ($P < 0.01$) (Supplementary Data 11).

Supplementary Note 17. Lineage-specific genes of the Asteraceae family

Lineage-specific genes such as orphan genes that represent genetic novelty arise within a lineage and commonly confer unique traits[66]. There are 114 OGs with four notable classifications particularly present in the Asteraceae family. An essential lineage-specific clade included transcription factor genes, such as bHLH, MYB, Znf, SQUAMOSA PROMOTER-BINDING PROTEIN-LIKE (SPL), and MADS-box, which we hypothesize participate in stress responses, flowering, and development (Supplementary Fig. 38; Supplementary Data 12; Supplementary Figs. 39–43). For example, the frequent divergent clades of *R2R3-MYB* genes are potentially involved in flower development and responses to biotic and abiotic stress (Supplementary Fig. 38)[67]. A substantial fraction (~10%) of lineage-specific clades consisted of genes that exhibited the highest sequence identity with the receptor-like protein kinase *FERONIA* gene family (Supplementary Table

22; Supplementary Fig. 43). The *FERONIA*-like families without a malectin-like domain greatly expanded, diverged, and were specific to Asteraceae (Supplementary Fig. 38; Supplementary Data 12, Supplementary Table 22). miRNA and degradome sequencing revealed that a newly identified and Asteraceae-specific miRNA targets and regulates the lineage-specific *FERONIA*-like genes in lettuce, indicating the possible dramatic co-evolution of miRNAs and *FERONIA* genes (Supplementary Fig. 38). A strengthened unique branch, including inulin biosynthesis genes, was uncovered and may potentially explain why inulin-type fructans, instead of starches, are the primary reserve carbohydrates in Asteraceae (Supplementary Fig. 38c; Supplementary Fig. 47 and 48). Another clade exhibited a high degree of similarity with strictosidine synthase, and raucaffricine-O-beta-D-glucosidase in Indian snakeroot (*Rauwolfia serpentina*) was detected (Supplementary Figs. 38d, 45 and 46), which is a member of the glycoside hydrolase family 1 and plays a vital role during alkaloid biogenesis[68]. Several key genes involved in cell wall biosynthesis and metabolism, including pectate lyase, pectinesterase (Supplementary Fig. 38e; Supplementary Table 23), filament-like plant protein, wall-associated receptor kinase, protein trichome birefringence-like, β-fructofuranosidase, and COBRA-like protein 4, were identified as specific clades, where the PME enriched in the TRRs is also included (Supplementary Data 12). Finally, a mixed group, including a large proportion (35/114) of specific clades, was classified as defense/stress-related (Supplementary Data 13).

Supplementary Note 18. Lineage-specific lost genes in the Asteraceae family

Besides the lineage-specific gain clades described above, we identified six clades lost in the Asteraceae family (Supplementary Table 24): thiopurine S-methyltransferase (TPMT), peptidase family C1 propeptide, ethylbenzene dehydrogenase, sulfite exporter TauE/SafE, protein phosphatase 2C, and nitrogen regulatory protein PII. We confirmed that three of these six domains (nitrogen regulatory protein PII, ethylbenzene dehydrogenase, and peptidase family C1 propeptide) were lost in all surveyed Asteraceae family members (Supplementary Table 37).

Supplementary Note 19. Confirmation of PII gene loss in the Asteraceae family

We identified a lineage-specific lost gene (*PII*) in the Asteraceae family by comparing the genomes of the 29 selected species (Supplementary Table 24). PII is a highly conserved regulatory protein found in organisms across the three domains of life. In cyanobacteria and plants, PII relieves the feedback inhibition of the rate-limiting step in arginine biosynthesis catalyzed by N-acetyl glutamate kinase (NAGK)[69]. To confirm the *PII* gene loss in the Asteraceae family, we extracted the *PII* gene family of all sequenced species from the OneKP database (https://db.cngb.org/onekp/) and investigated the *PII* gene status in all these species. In parallel, we downloaded the transcriptomes of all species and confirmed the existence of the *PII* gene using the BLASTN program. There were eight families and 39 species from Asterales (31 from Compositae, 2 from Campanulaceae, and 1 from each of Phellinaceae, Stylidiaceae, Argophyllaceae, Goodeniaceae, Menyanthaceae, and Alseuosmiaceae) (Supplementary Table 25). We did not find the *PII* gene in any of the 39 species of the Asteraceae family or its Goodeniaceae relative (Fig. 3a). We inferred that *PII* genes might be

lost in the ancestor of the Goodeniaceae and Compositae family (Supplementary Table 25, Supplementary Data 14).

We traced the history of *PII* gene loss using colinearity analysis. We selected the well-assembled *D. carota* genome as the reference and conducted pairwise colinearity analysis using Mcscan software[56]. There was good colinearity around *PII* genes between *D. carota* and *Sc. taccada*, although there was evidence of chromosomal rearrangement. As for the three chromosome-scale reference genomes of the Asteraceae family (*La. sativa*, *H. annuus*, and *C. cardunculus*), there was a micro-inversion (involving 20–30 genes and encompassing less than 1 Mb in the *D. carota* genome) between *D. carota* and others; the *PII* gene was located on the border of the rearrangement region (Fig. 3b–d). Chromosomal inversions are usually portrayed as simple two-breakpoint rearrangements changing gene order but not gene number or structure. However, increasing evidence suggests that inversion breakpoints may often have a complex structure and entail gene duplications with potential functional consequences.

Supplementary Note 20. Biological influence of the loss of PII in the Asteraceae family

PII is a chloroplast-localized N sensor that activates the *N*-acetyl-L-glutamate kinase (NAGK) complex to promote N assimilation[70]. The PII proteins act as reporters of the C metabolic state of the cell by interdependently binding ATP/ADP and 2-oxoglutarate (2-OG)[71]. At the same time, the levels of cellular glutamine in plants are additionally sensed via PII signaling[69]. The glutamine-sensing mechanism based on the canonical PII signaling machinery is common in the entire plant kingdom except Brassicaceae[69]. To be specific, the plant-specific C-terminal extension of PII (Q loop) forms a low-affinity glutamine-binding site and the binding motif is highly conserved in plants except Brassicaceae[69]. Besides, PII forms a complex with the biotin carboxyl carrier protein (BCCP) subunit of acetyl-CoA carboxylase (ACCase, which catalyses the first step in fatty acid biosynthesis) to inhibit ACCase activity[72]. The functional studies of *At-PII* are the most abundant in Arabidopsis[72–75]. For example, there are overexpression and knock-out (mutant) studies of *At-PII*, respectively[72–75].

Firstly, we conducted the multiple sequences alignment using the PII proteins from *Polytomella parva*, *Chlamydomonas reinhardtii*, *Physcomitrella patens*, *Oryza sativa*, *Arabidopsis thaliana*, *Solanum lycopersicum* and *Dancus carota*. The multiple sequence alignment indicated that there exist the intact plant-specific C-terminal extension of PII (Q loop) in the PII sequences of *Solanum lycopersicum* and *Dancus carota* (Supplementary Fig. 49). Thus, we deducted that the ancestor of Asteraceae should have a canonical PII protein sequence that similar to that of carrot/tomato. Secondly, to evaluate the biological influence of PII absence in Asteraceae, we generated transgenic lettuce plants expressing a PII gene from Arabidopsis (*A. thaliana*), carrot (*Da. Carota*) and tomato (*Solanum lycopersicum*), respectively (Supplementary Fig. 50). Thirdly, to demonstrate the biological function of these *PIIs*, we conducted the i*n vivo* assays (Bimolecular Fluorescence Complementation, BiFC) and *in vitro* tests (Pull-down), which strongly supported the interactions between AtPII, DcPII, SlPII and NAGK in lettuce (Fig. 3e, 3f; Supplementary Figs. 51, 52).

Therefore, we think that the PIIs transformed into lettuce can play their biological function. Finally, we measured key physiological and biochemical indicators in PII signaling machinery including amino acid, ACCase activity, and nitrate nitrogen content. We observed that in the *DcPII*-OE and *SlPII*-OE lines, the total content of free amino acids, especially glutamic acid, glutamine, and arginine, was significantly higher than that of empty vector/wild-type control (Supplementary Fig. 52). In contrast, the total content of free amino acids (and glutamic acid, glutamine, and arginine) was decreased significantly in the overexpressing *At-PII* lines (Supplementary Fig. 51). The nitrate nitrogen content and ACCase activity were significantly lower in all the transgenic plants (Supplementary Figs. 51 and 52).

Supplementary Note 21. In-depth discussion and interpretation of PII-related experiments

To investigate the biological influences of the PII loss in Asteraceae, we selected two typical PIIs in plants to conduct the functional study: 1) Canonical PIIs with intact Q loops (Gln-dependent PII-NAGK activation). The canonical PII signaling machinery is a widespread glutamine-sensing mechanism in the plant kingdom[69]. Based on the phylogeny and sequence alignment, the ancestor of Asteraceae was inferred to have the canonical PII. 2) PII with a Brassicaceae lineage-specific deletion in the Q loops (Gln-independent PII-NAGK activation). As the model of higher plants, the functional studies of At-PII are the most abundant in Arabidopsis[72–75]. For example, there are overexpression and knock-out (mutant) studies of At-PII, respectively[72–75]. Therefore, our transgenic studies can be compared with these studies side-by-side.

The subcellular localization experiments and BiFC assay indicated the plastid-localization of PII, which was consistent with the previous study. A series of *in vivo*/*vitro* experiments validated the interactions between PIIs (from Arabidopsis, carrot, and tomato, respectively) and NAGK (from lettuce), suggesting the conservation of the PII-NAGK activation during evolution. Consistently, the PII-NAGK proteins from *Synechococcus elongatus* and *A. thaliana* functionally complement each other in vitro, highlighting the functional conservation[76].

Another target of PII proteins that is conserved between bacteria (including cyanobacteria) and plants is the BCCP subunit of ACCase, which controls the rate-limiting step of fatty acid biosynthesis[72,77]. PII protein forms complexes with the BCCP subunit of ACCase, thus reducing ACCase activity due to a decrease in the Kcat[72]. In our study, we observed that the ACCase activity decrease significantly in all three transgenic lines, which was consistent with previous studies[72].

In cyanobacteria, PII mediates the ammonium- and dark-induced inhibition of nitrate uptake by interacting with the NrtC and NrtD subunits of the nitrate/nitrite transporter NrtABCD[78]. In plants, the PII mutants of *A. thaliana* showed higher nitrite uptake and sensitivity implying that the PII-mediated regulation of nitrite uptake is potentially similar to that by cyanobacteria[79,80]. This indicates that PII is needed to prevent overexcess of nitrite uptake. When overexpressing the PIIs in lettuce, the nitrate nitrogen content in all the transgenic lines was decreased, implying the inhibitory effect of PII on nitrogen uptake.

The binding of PII enhances NAGK activity in the presence of the feedback inhibitor arginine, an effect that can be antagonized by the PII-effector molecule 2-OG[81,82]. The arginine, ornithine, and citrulline concentrations were reduced in an *A. thaliana* PII mutant[74]. The PII mutants displayed a slight increase in carbohydrate (starch and sugars) levels in response to N starvation and a slight decrease in the levels of ammonium and amino acids (mainly Gln) in response to ammonium resupply[79]. Here, in the overexpressing Dc-PII and SI-PII lines, we observed that the arginine glutamine and glutamic acid increased, which can be explained by the increased NAGK activity. In contrast, we got an opposite observation in overexpressing At-PII lines. Although the specific reasons deserve to be explored, differences in the Q loop region (glutamine-binding site) of PII proteins were highly suspected to have an effect. Overall, the strong changes in the metabolism of the PII-expressing transgenic plants indicated that exogenous PII could disturb the original N-C balance in lettuce.

Given that the loss of PII in Asteraceae might occur in the ancestor of Goodeniaceae and Asteraceae (~80 MYA), the unique N-C balance system in Asteraceae evolved for a long history, potentially resulting in complicated changes when compared to other plants with PII. More studies to fully investigate the physiological and metabolic adaptation machinery based on the unique N-C balance system in Asteraceae are further needed.

Supplementary Note 22. Nitrogen uptake and metabolism in Asteraceae

The *nitrate transporter 2* (*NRT2*) family is involved in high-affinity nitrate transport, and the *nitrate transporter 3/nitrate assimilation-related* (*NRT3/NAR*) genes act as a dual transporter with *NRT2*[83]. The investigation of key genes in nitrogen uptake and metabolism indicated that both *NRT2* and *NRT3* families in Asteraceae were significantly larger than in other selected species, including *Sc. taccada* ($P < 0.01$) (Figs. 4a, f; Supplementary Figs. 53-56), which is consistent with the rapid expansion of orthologous gene families associated with the nitrogen reservoir in the crown node of Asteraceae (Supplementary Fig. 35). A colinearity analysis between stem lettuce and *Sc. taccada* showed that the expansion of these two gene families was possibly initiated by the WGT-1 event and largely increased by tandem duplications (Fig. 4c,h).

Specifically, the NRT2 proteins from Asteraceae were distributed in four main clades in the phylogenetic tree based on all encoded proteins from the 29 investigated species (Fig. 4d; Supplementary Fig. 53). The proteins in clade IV were highly homologous to Arabidopsis NRT2.5 (At1g12940), which is involved in the constitutive high-affinity transport system under long-term nitrogen starvation conditions[84]. There was no apparent gene amplification of this clade in Asteraceae. By investigating the gene expression of members of this clade, we observed that these genes were rarely expressed in roots (Fig. 4e), which is similar to the expression pattern in Arabidopsis. A similar situation was observed within clade III: There was no expansion of the gene number, and no genes were primarily expressed in roots, while the homologous Arabidopsis gene *NRT2.7* (At5g14570) is involved in high-affinity nitrate transport and controls the nitrate contents in seeds (Supplementary Figs. 53 and 54)[85]. The genes of Asteraceae were primarily distributed in

clades I and II, and nearly all these genes were expressed in roots. Clades I and II harbored key members of high-affinity nitrate transporters in Arabidopsis, including NRT2.1 (At1g08090), NRT2.2 (At1g08100), NRT2.3 (At5g60780), NRT2.4 (At5g60770), and NRT2.6 (At3g45060), and NRT2.1 or NRT2.3 was involved in nitrate transport and acted as a dual transporter with NTR3.1. We observed that the gene copy numbers of many members in clades I and II increased from tandem duplications (Fig. 4h), and their primary expression in roots suggested that these genes may function in roots and play an essential role in nitrogen uptake. NRT3 members in Asteraceae were distributed in three clades (I, II, and III). Genes in clades II and III contained more duplicates, and nearly all of the genes in the two clades were most highly expressed in roots (Fig. 4i, j). We calculated the *Ka*/*Ks* value of orthologous gene pairs (*Sc. taccada* versus lettuce) and paralogous gene pairs (lettuce versus lettuce) of the NRT2 clade II (and the NRT3 clade III) members and observed that all duplicated genes were subjected to purifying selection during evolution (Fig. 4b, g).

Supplementary Note 23. Central carbon metabolism and fatty acid metabolism in Asteraceae

PII plays a vital role in regulating the nitrogen–carbon balance. We investigated the copy number of central carbon metabolism genes in Asteraceae. As one of the most critical metabolism pathways, fatty acid metabolism provides the basic carbon skeletons for amino acid and secondary metabolism. The essential functional domains encoded by crucial genes in the metabolism of fatty acid biosynthesis were enriched in Asteraceae: *KAS*s, *ADS*s, and *FAD*s (Supplementary Figs. 57 and 58).

This observation suggested that the genes associated with fatty acid metabolism in Asteraceae were enriched in TRRs (Supplementary Fig. 17), rapidly expanded (Supplementary Fig. 37), and present in large numbers in the InterPro entries (Supplementary Fig. 57). The key genes in fatty acid biosynthesis were identified in detail, and we found that three such families had significantly expanded in Asteraceae compared to the other selected species ($P < 0.01$) (Fig. 5a; Supplementary Figs. 57 and 58).

First, 366 ketoacyl synthases (KASI, II, and lII), which can catalyze all the condensation reactions of fatty acid biosynthesis by adding an acyl acceptor of two carbons from malonyl-ACP (Supplementary Fig. 59), were identified in different species[86], and the *KAS* gene copy number in Asteraceae was high (Fig. 5; Supplementary Figs. 57–60). Second, the number of fatty acid desaturase (*FAD*) genes in the Asteraceae group was significantly greater than that in the other species ($P < 0.01$) (Fig. 5a; Supplementary Figs. 58 and 62). As the neighbor taxa, only four members of *FAD* genes in *Sc. taccada* were detected, significantly fewer than that in Asteraceae (at least 14 gene copies) (Fig. 5a, c). The constructed phylogenetic tree based on 540 FAD proteins identified across the 29 species showed two clusters (clusters I and II) containing most FAD proteins in Asteraceae, suggesting the expansion of genes in specific types of FADs (Fig. 5c; Supplementary Fig. 62). Third, delta-9 acyl-lipid desaturase proteins (or delta-9 desaturase-like proteins), which are involved in the delta-9 desaturation of fatty acids and play an essential role in the biosynthesis of polyunsaturated fatty acids[87], were also encoded by significantly more gene copies in Asteraceae

than in the other species (Fig. 5a, b; Supplementary Fig. 63). In addition, expression pattern examined by qRT-PCR suggests these expanded genes are expressed and functional (Supplementary Figs. 64 and 65). The reason for gene expansion in these three families was traced: In all cases, the WGT-1 event might have led to the initial expansion, and gene tandem duplications were the primary reason (Fig. 5).

The plastid pyruvate dehydrogenase complex generates acetyl-coenzyme A that is used as a building block for fatty acid production. Fatty acid chains extend by sequential condensation of two-carbon units catalyzed by enzymes of the fatty acid synthase complex. During each cycle, four reactions take place: condensation, reduction, dehydration, and reduction. Acyl carrier protein is a cofactor in all reactions. Biosynthesis of a C16 fatty acid requires that the cycle be repeated seven times. During the first round of the cycle, the condensation reaction is catalyzed by ketoacyl-ACP synthase (KAS) III. For the next six rounds of the cycle, the condensation reaction is catalyzed by isoform I of KAS. Finally, KAS II is used during the conversion of 16:0 to 18:0.

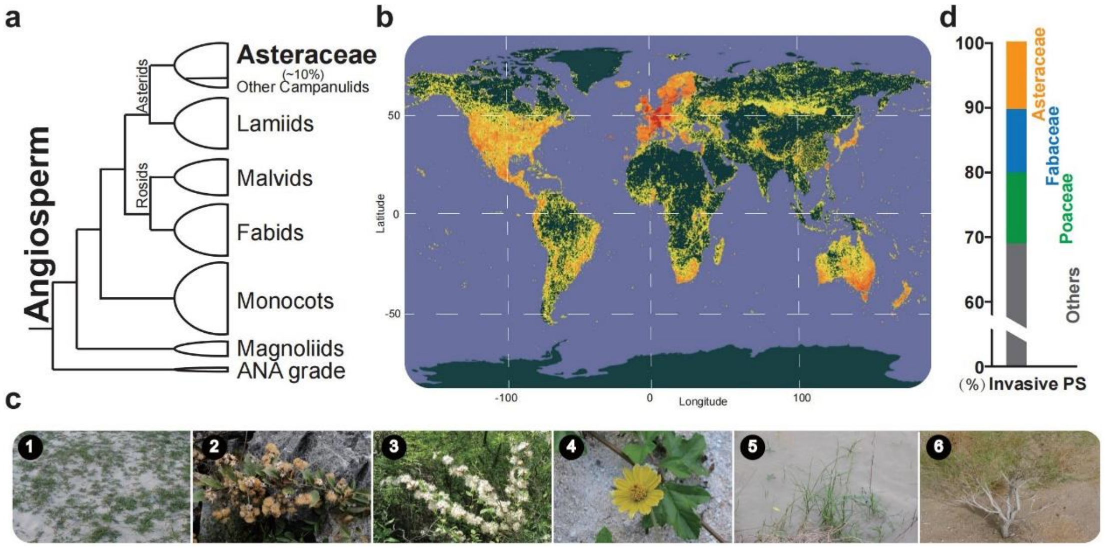

Supplementary Fig. 1. Species and habitat diversity of Asteraceae. a, Species diversity of Asteraceae according to the APG IV system of flowering plant classification. The breadth of the half ellipses indicates the species number corresponding to each phylum (data resource: https://www.ncbi.nlm.-nih.gov/taxonomy/). **b**, Geographical distribution of the Asteraceae. The original data are from Global Biodiversity Information Facility (https://www.gbif.org/). **c**, Representative Asteraceae plants living in different habitats. 1, *Launaea sarmentosa* in a tropical beach; 2, *Vernonia chingiana* in a tropical lime mountain; 3, *Myripnois dioica* in a temperate broadleaf forest; 4, *Sphagneticola calendulacea* in a tropical sand beach; 5, *Sheareria nana* in a subtropical riverbank; 6, *Artemisia desertorum* in a temperate desert. **d**, Ratio of worldwide invasive plant species (PS). The data resource is Global Invasive Species Database (http://www.iucn-gisd.org/gisd/). Source data are provided as a Source Data file.

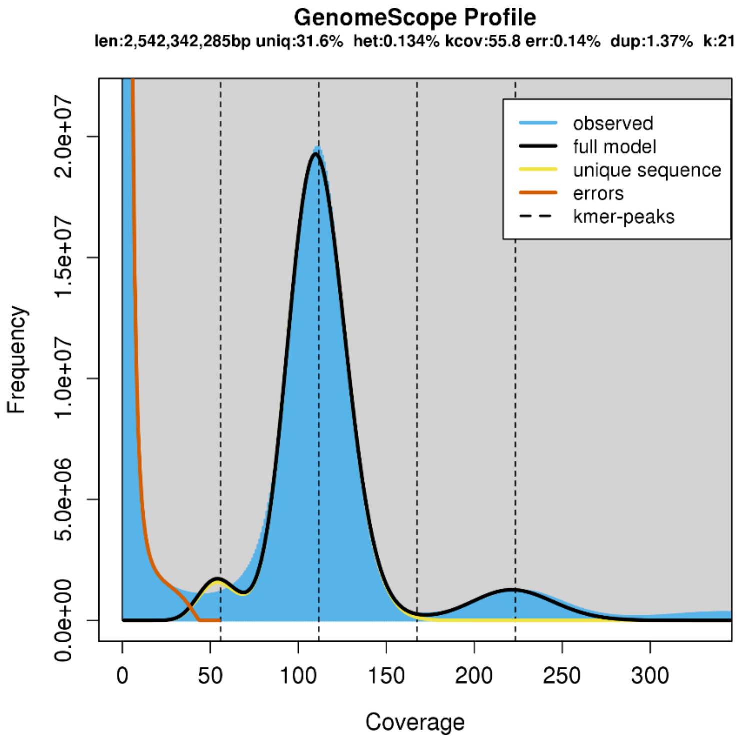

Supplementary Fig. 2. Genome size estimation based on *k*-mer statistics for *Lactuca sativa* var. *angustana*.

GenomeScope was employed to estimate heterozygosity and size under the 21-mer distribution. Peaks at ~55, 110, and 225 represent heterozygous, homozygous, and repeated *k*-mers, respectively.

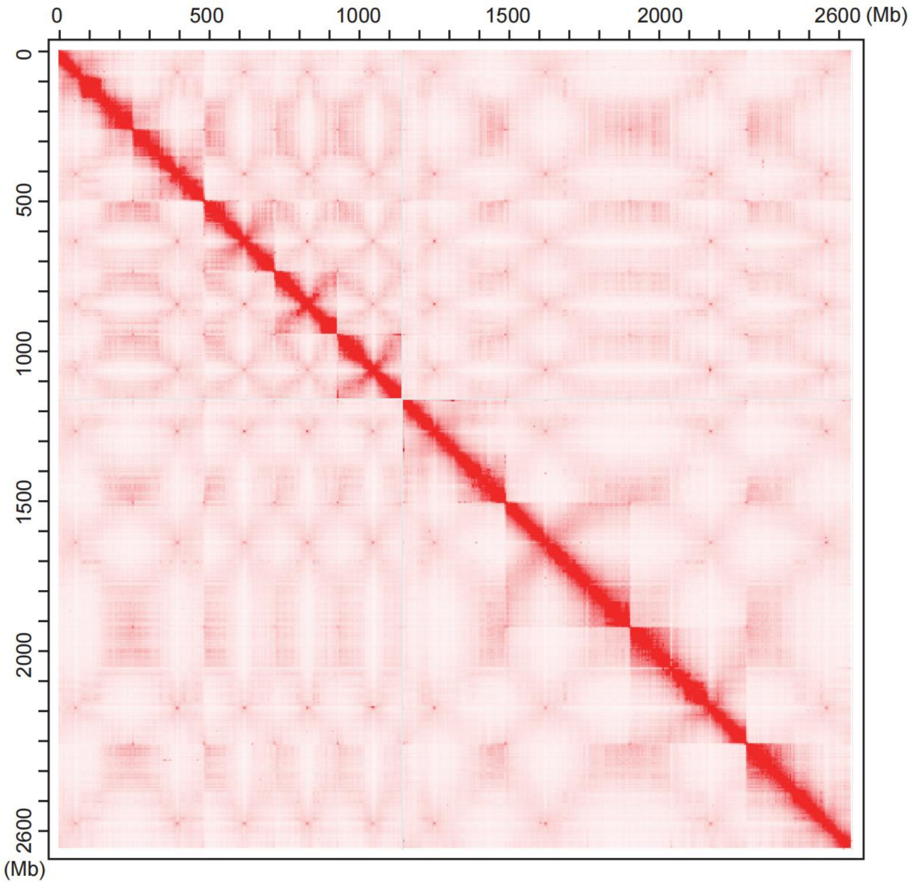

Supplementary Fig. 3. Hi-C interactions among nine pseudochromosomes with a 100-kb resolution (*Lactuca sativa* var. *angustana*).

Strong interactions are indicated in dark red; the nine blocks indicate nine pseudochromosomes.

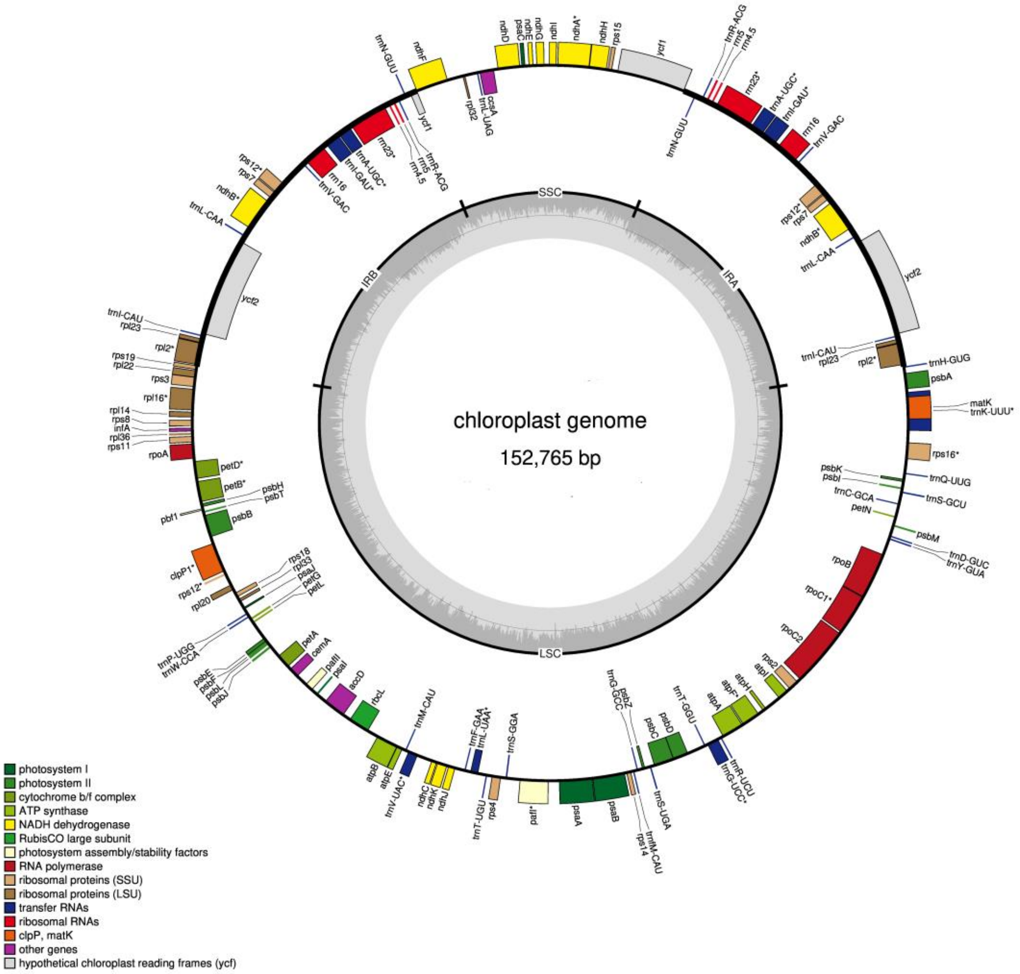

Supplementary Fig. 4. Gene map of the chloroplast genome (*Lactuca sativa* var. *angustana*).

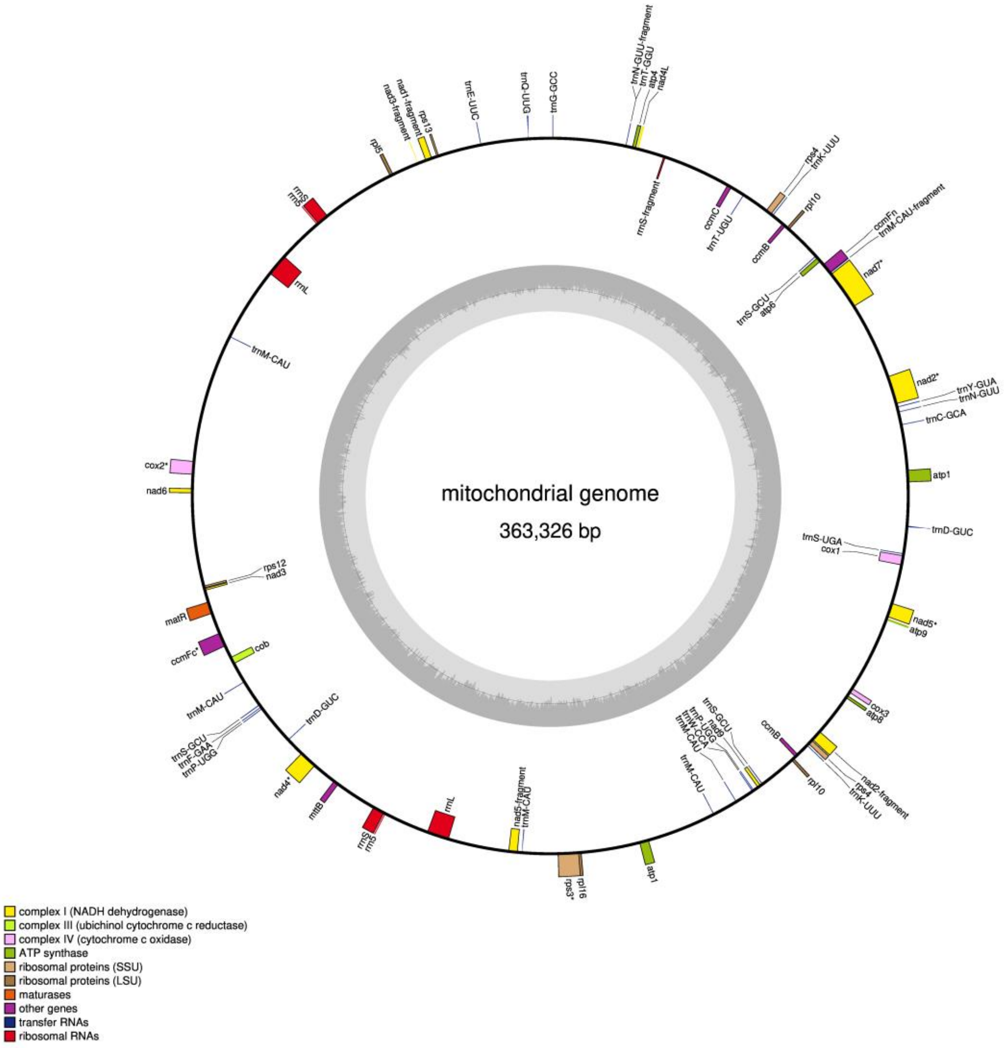

Supplementary Fig. 5. Gene map of the mitochondrion genome (*Lactuca sativa* var. *angustana*).

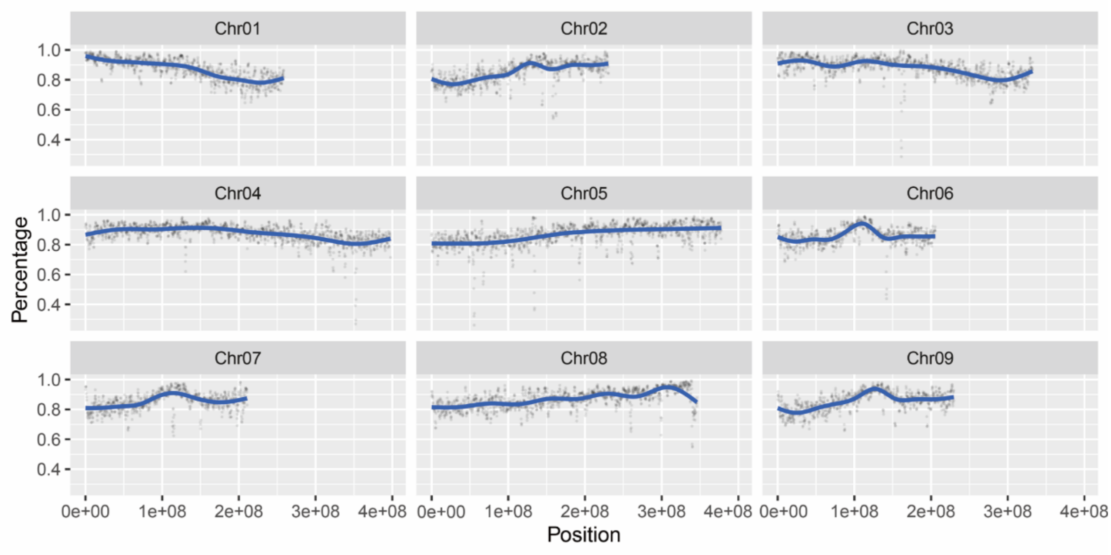

Supplementary Fig. 6. Distribution of repeat sequences across the nine pseudochromosomes of stem lettuce (*Lactuca sativa* var. *angustana*).

The y-axes indicate the percentage of repeat sequence in 1-Mb windows with a 100-kb sliding step; the x-axes show the length of each chromosome.

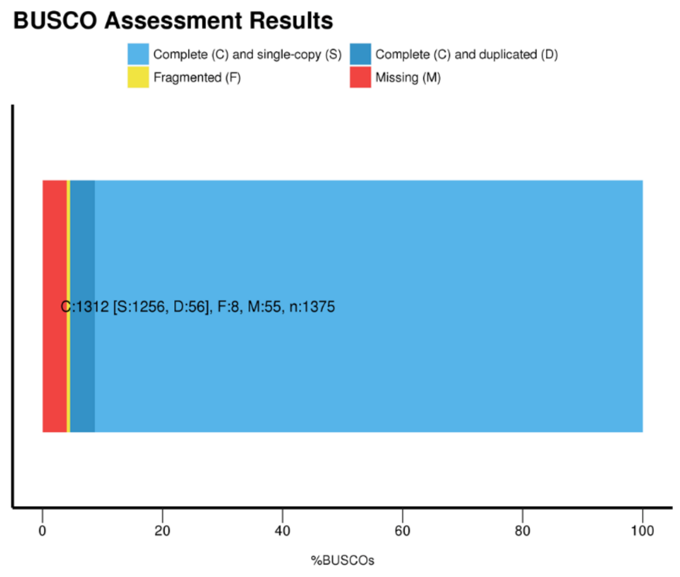

Supplementary Fig. 7. Genome assessment by Benchmarking Universal Single-Copy Ortholog (BUSCO) evaluation (*Lactuca sativa* var. *angustana*).

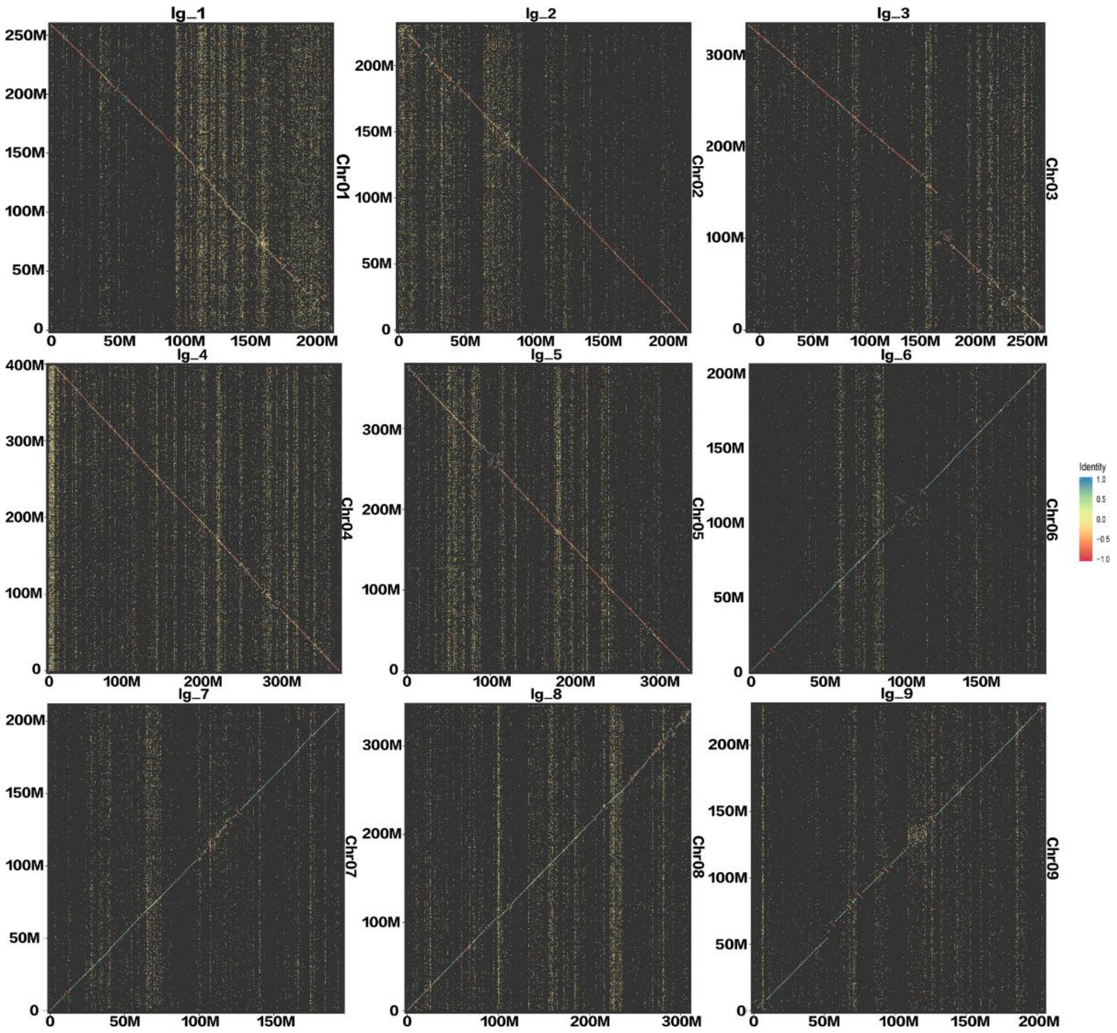

Supplementary Fig. 8. Dot plot of the pairwise alignment of stem lettuce (*Lactuca sativa* var. *angustana*) and crisp lettuce (*Lactuca sativa* var. *crispa*) genome assemblies.

The x-axes represent chromosomes from the assembly of crisp lettuce, and the y-axes indicate chromosomes from the assembly of stem lettuce.

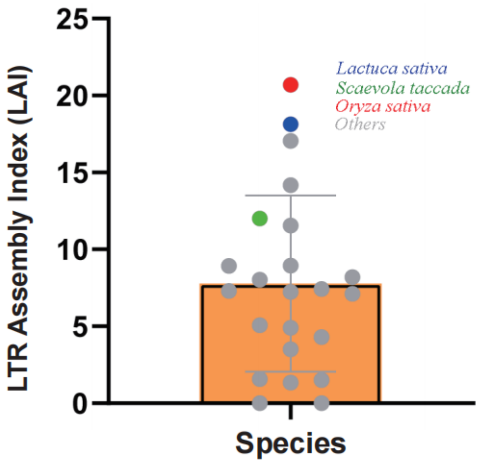

Supplementary Fig. 9. LTR Assembly Index comparison among selected model plant species. Other species include *Helianthus annuus*, *Cynara cardunculus*, *Chrysanthemum nankingense*, *Artemisia annua*, *Daucus carota*, *Actinidia chinensis*, *Coffea arabica*, *Mimulus guttatus*, *Olea europaea*, *Solanum lycopersicum*, *Capsicum annuum*, *Fragaria vesca*, *Vitis vinifera*, *Solanum tuberosum*, *Gossypium hirsutum*, *Hordeum vulgare*, *Taraxacum kok-saghyz* Rodin, *Mikania micrantha*, *Ananas comosus*, *Amborella trichopoda*, *Selaginella moellendorffii*, *Spirodela polyrhiza*, *Brassica rapa*, *Populus trichocarpa*, and *Citrus sinensis*. In the box plot, bounds of box: 25th and 75th percentiles; whiskers: 1.5 * IQR (IQR: the interquartile range between the 25th and 75th percentile). Source data are provided as a Source Data file.

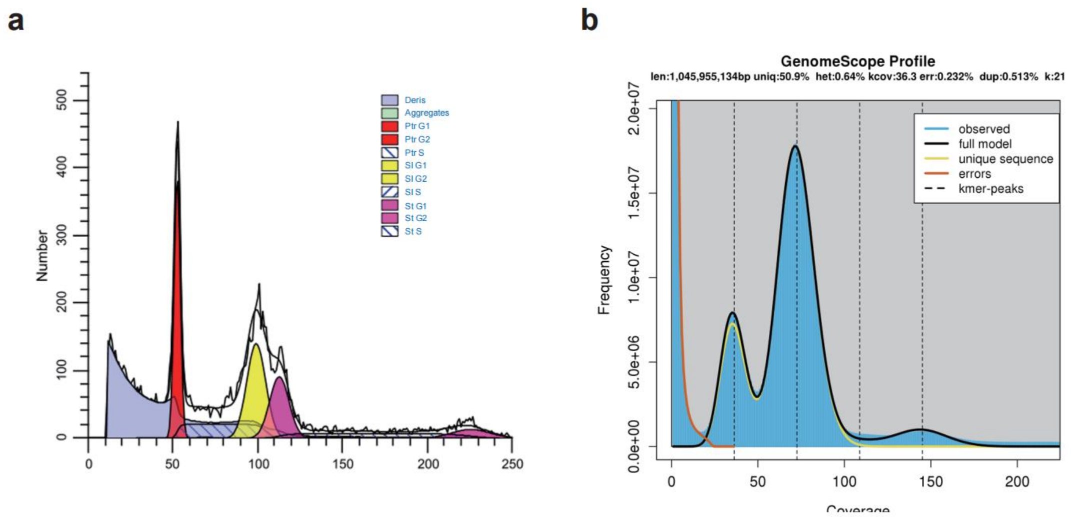

Supplementary Fig. 10. Genome size estimation based on flow cytometry studies and *k*-mer statistics (*Scaevola taccada*).

a, *Populus trichocarpa* and *Solanum lycopersicum* were employed as internal references. The estimated genome size of *Sc. taccada* is around 1,070 Mb. *P. trichocarpa*: Ptr G1/G2/S; *S. lycopersicum*: Sl G1/G2/S; *Sc. taccada*: St G1/G2/S. **b**, Genome size estimation based on *k*-mer statistics. As described in Data S1A, GenomeScope was also employed to perform the estimation, and peaks at ~37, 70, and 145 represent heterozygous, homozygous, and repeated *k*-mers, respectively.

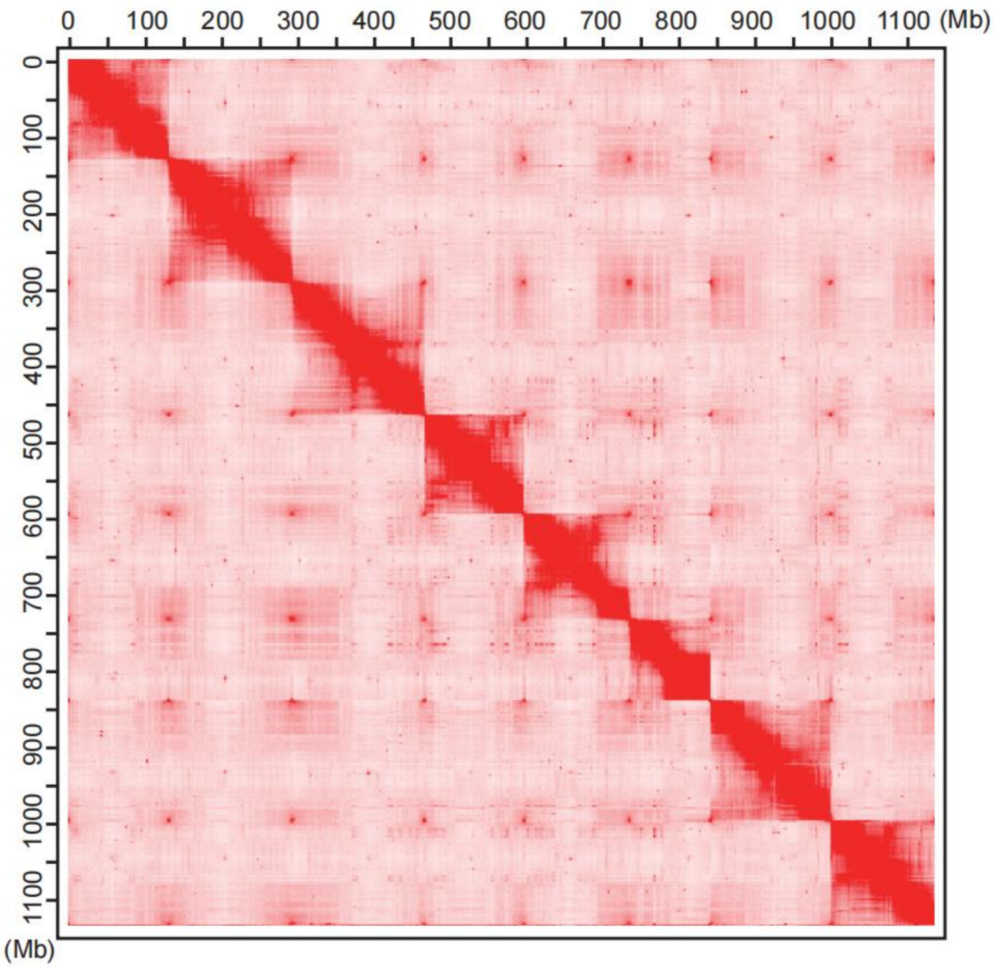

Supplementary Fig. 11. Hi-C interaction map of the *Scaevola taccada* pseudochromosomes. Strong interactions are indicated in dark red; eight blocks indicate eight pseudochromosomes.

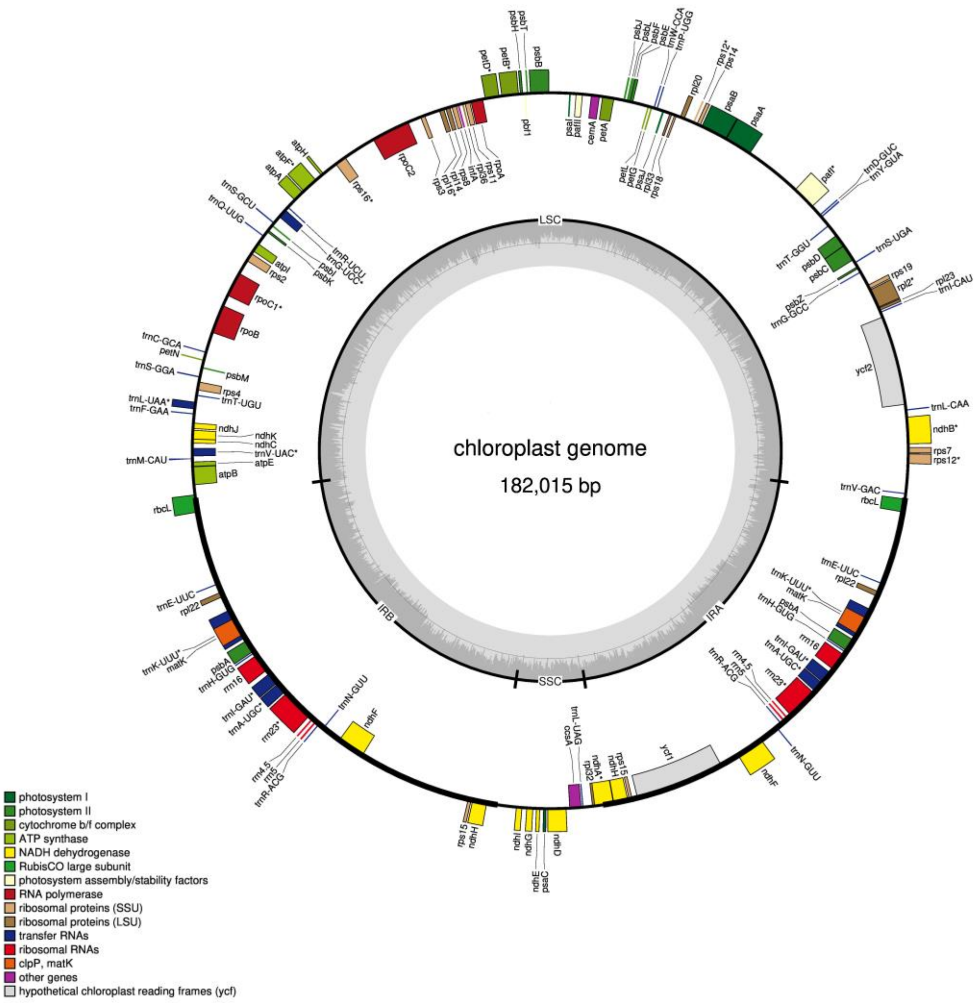

Supplementary Fig. 12. Gene map of the chloroplast genome (*Scaevola taccada*).

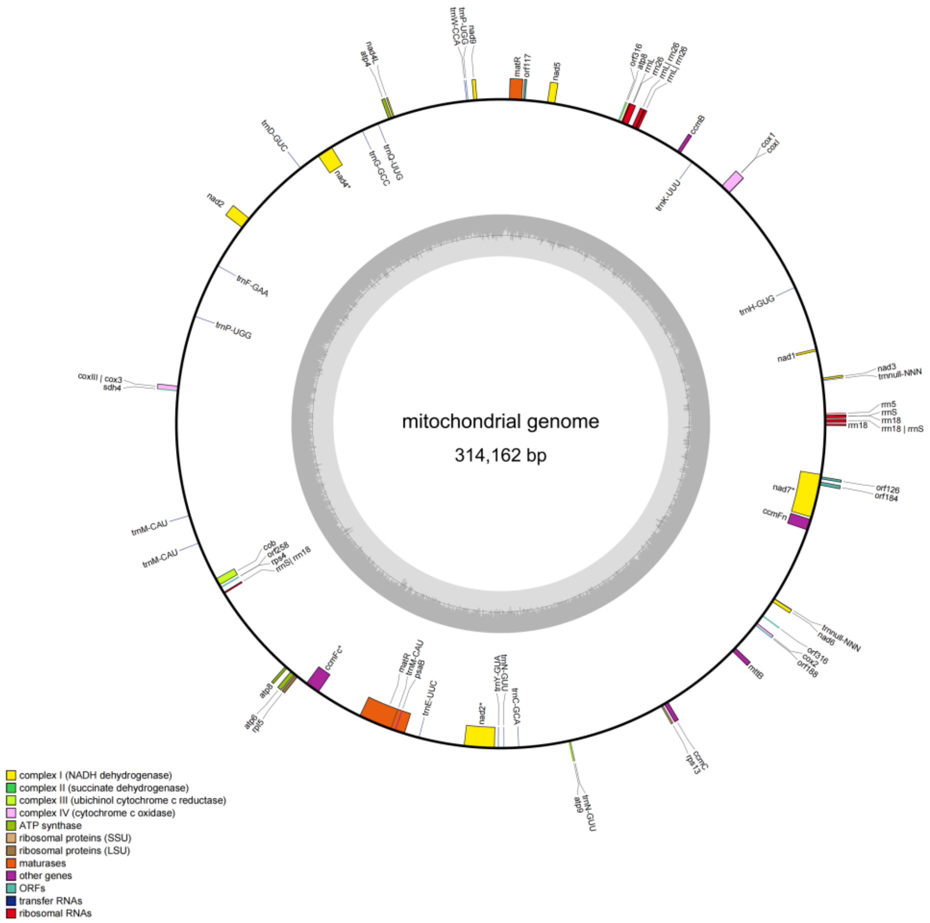

Supplementary Fig. 13. Gene map of the mitochondrion genome (*Scaevola taccada*).

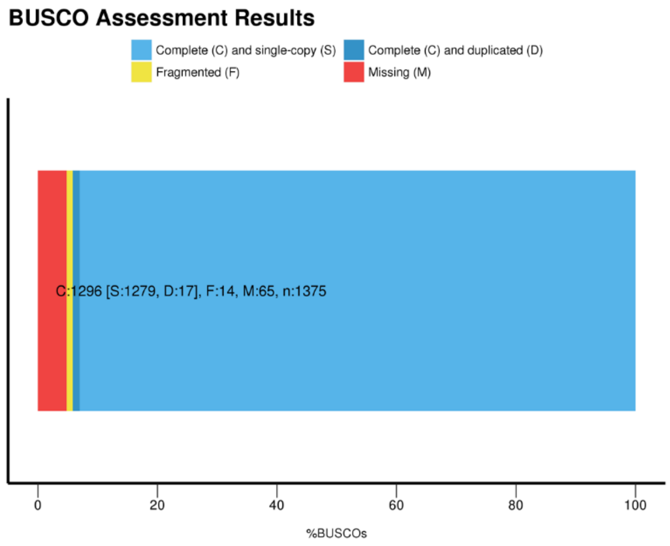

Supplementary Fig. 14. Genome assessment by Benchmarking Universal Single-Copy Ortholog (BUSCO) evaluation (*Scaevola taccada*). Source data are provided as a Source Data file.

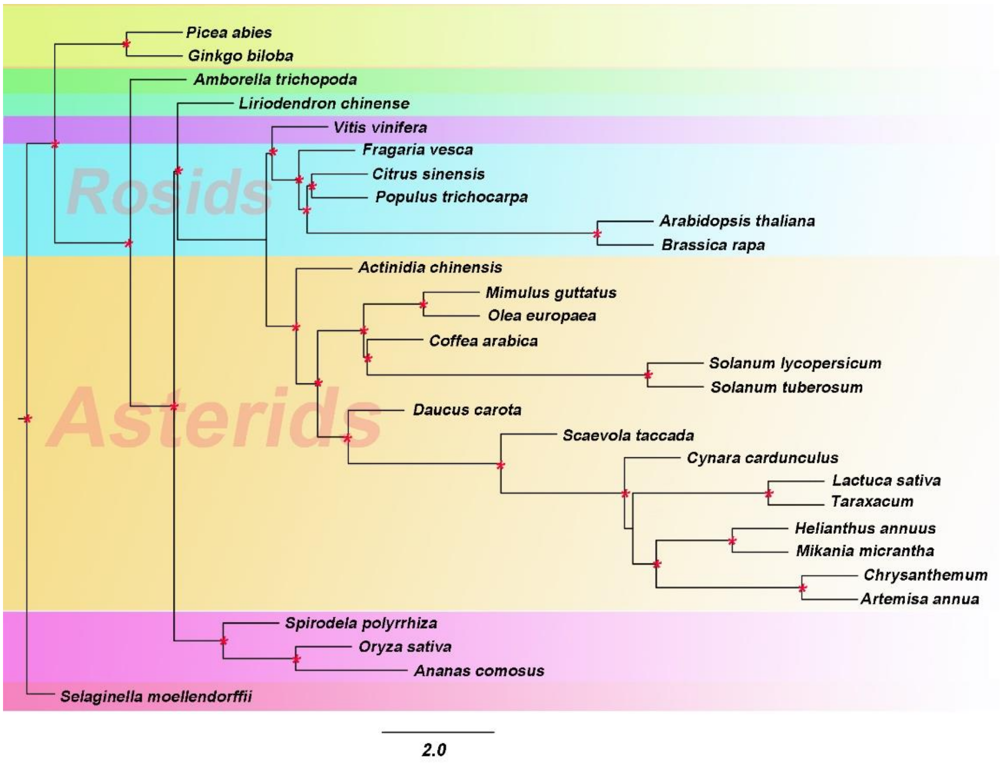

Supplementary Fig. 15. Phylogenetic tree of the 29 selected species inferred from the coalescent-based analysis.

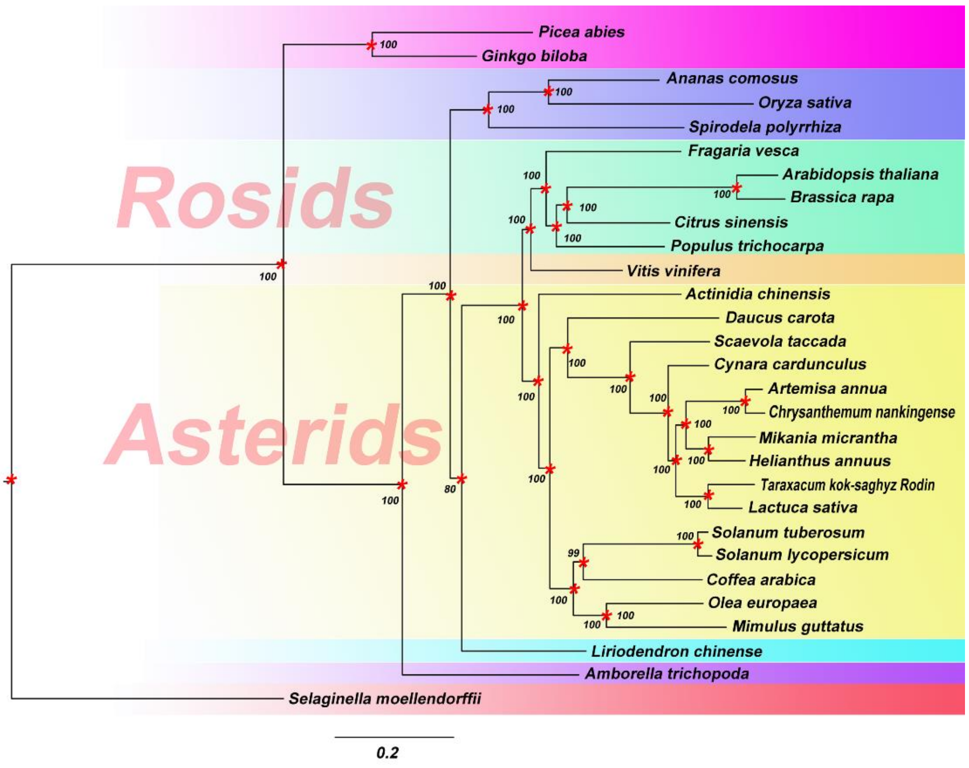

Supplementary Fig. 16. Phylogenetic tree of the 29 selected species reconstructed using the concatenated protein sequence of low-copy-number genes.

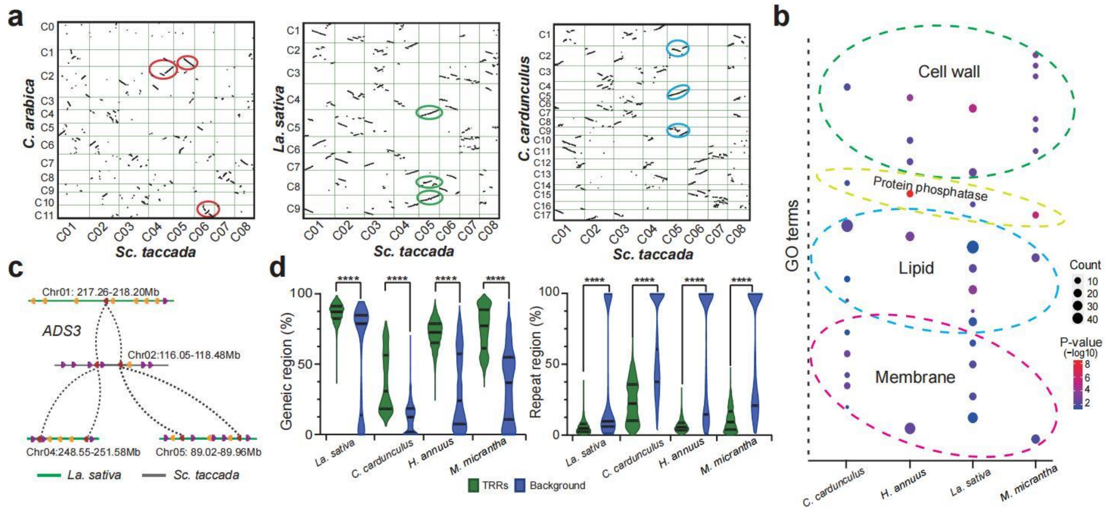

Supplementary Fig. 17. Whole-genome triplication-related genomic features in Asteraceae. a, Whole-genome syntelog visualization of *Sc. taccada* versus coffee (*C. arabica*) and two Asteraceae species, lettuce (*La. sativa*) and artichoke (*C. cardunculus*), respectively. Red circles indicate a 1:1 relationship, while green and blue circles show a 1:3 relationship. **b,** Enriched gene ontology terms of the genes in the triplication retained regions (TRRs) of four representative Asteraceae species. **c,** Microsynteny visualization of the *ADS3* syntelog pairs in the TRRs of lettuce and *Sc. taccada*. **d,** Percentages of genetic/repeat regions in the TRRs and the whole genome in four selected Asteraceae species. A sliding window of 1 Mbp was used to calculate each data point. ****, P <0.00001 as determined by two-tailed Student's t-test. Source data are provided as a Source Data file.

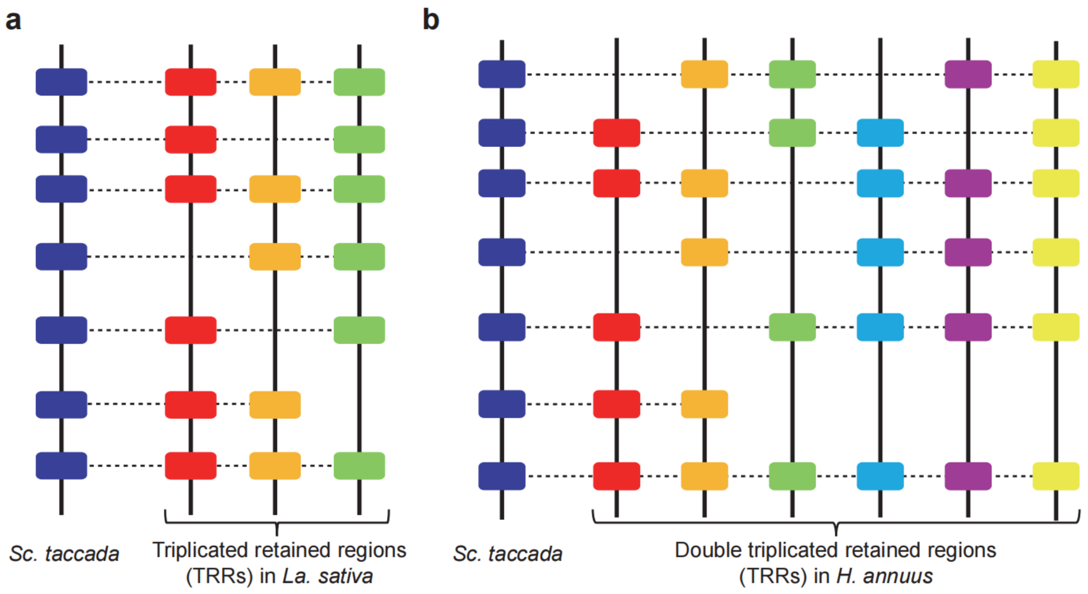

Supplementary Fig. 18. Schematic diagram of triplication-retained region (TRRs).

a, Definition of TRRs for genomes only with the shared whole-genome triplication event (WGT-1) by the Asteraceae species such as *Lactuca sativa* var. *angustana* and *Cynara cardunculus* var. *scolymus*. **b**, Definition of TRRs for genomes with another whole-genome duplication after the WGT-1 event, such as *Helianthus annuus*.

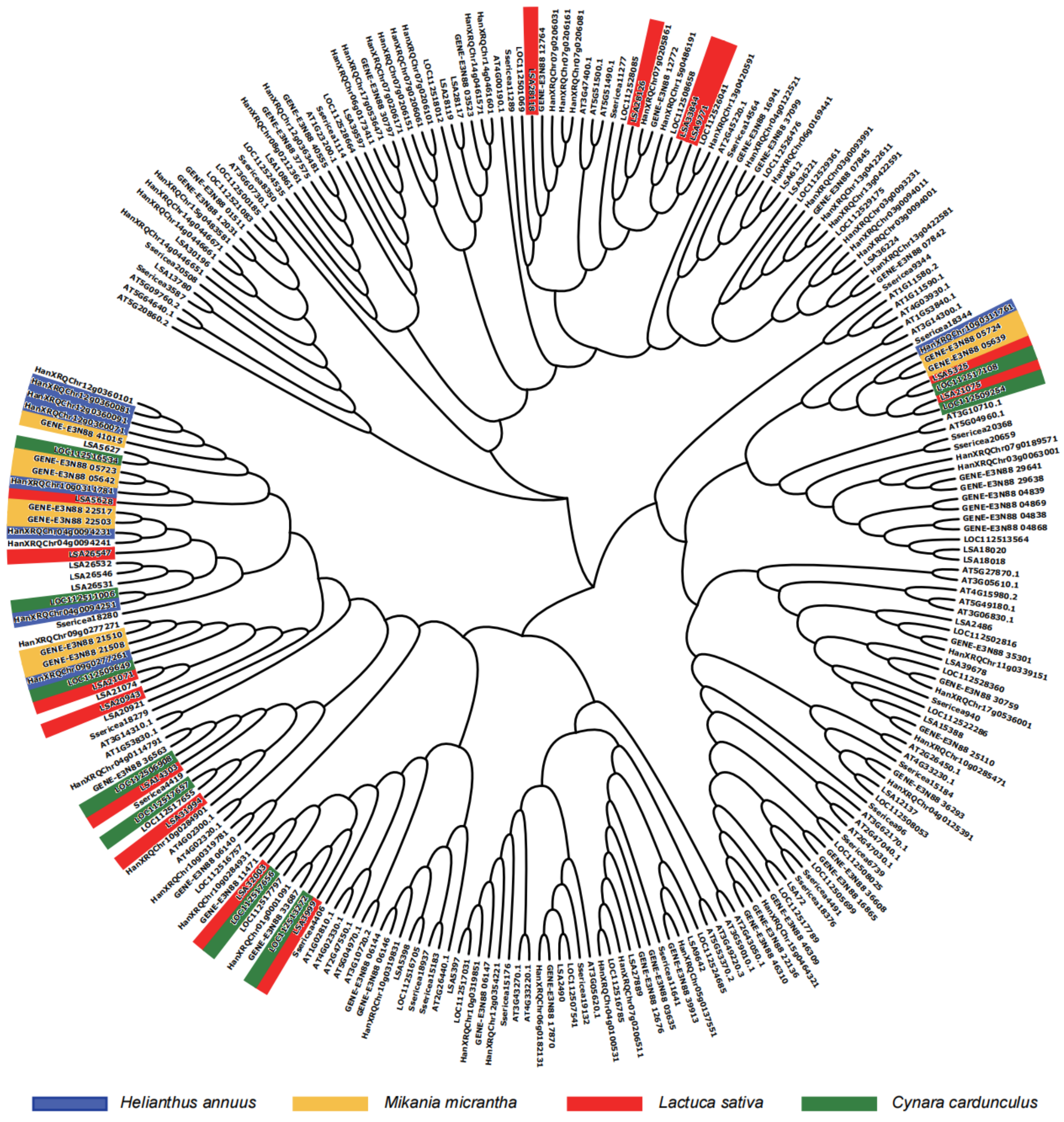

Supplementary Fig. 19. Phylogenetic tree of pectinesterases in the four Asteraceae species with high-quality genomes.

Genes encoding pectinesterases were identified in the four Asteraceae species *Lactuca sativa* var. *angustana*, *Cynara cardunculus* var. *scolymus*, *Helianthus annuus*, and *Mikania micrantha.* Genes in the triplication-retained regions are marked with different colors.

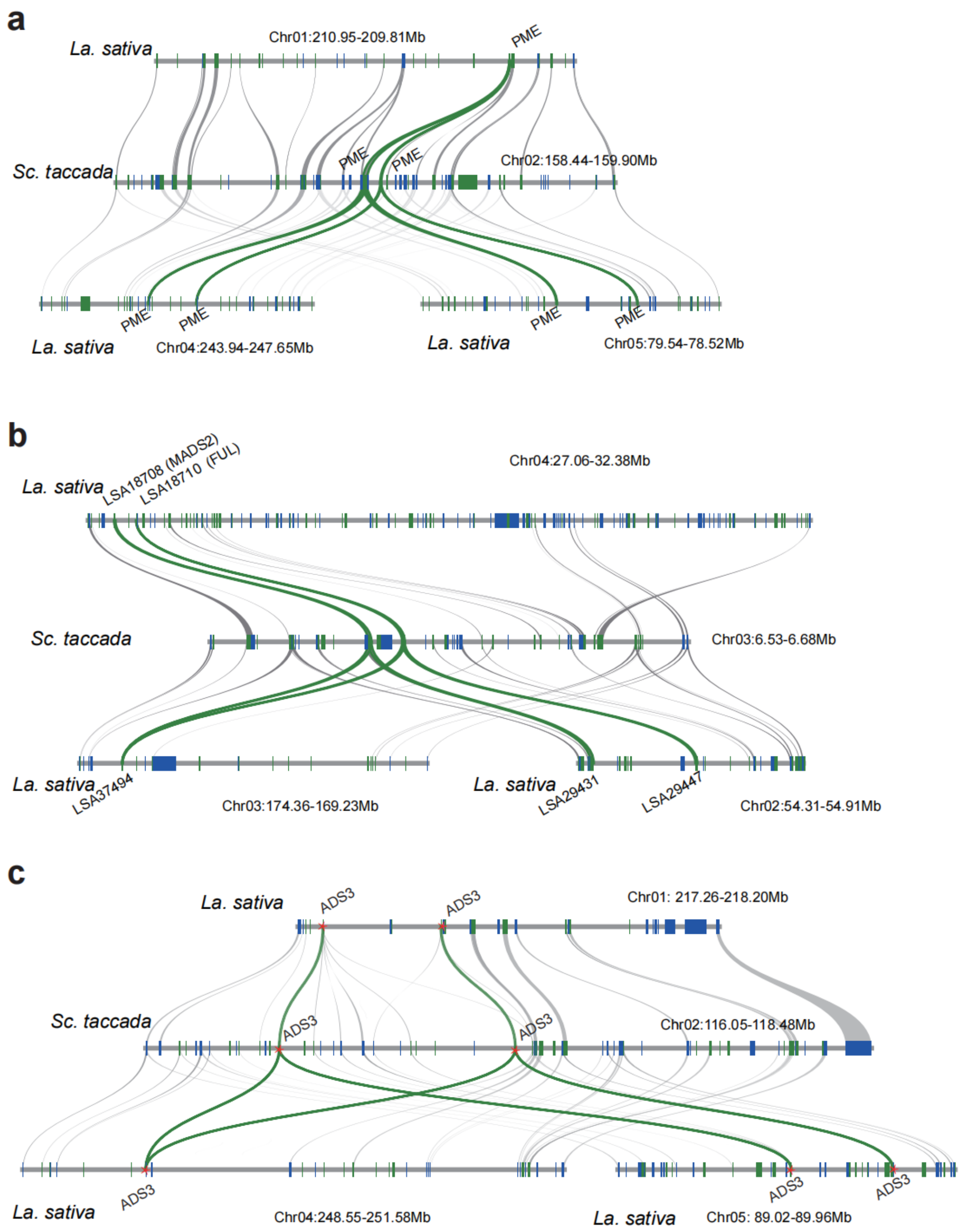

Supplementary Fig. 20. Three typical examples of triplication-retained regions (TRRs) between *Lactuca sativa* var. *angustana* and *Scaevola taccada*.

Micro-synteny visualization of (**a**) pectinesterase (*PME*), (**b**) MIKC-type MADS-box, and (**c**) palmitoyl-monogalactosyldiacylglycerol delta-7 desaturase (*ADS3*) genes in TRRs between *La. sativa* and *Sc. taccada*.

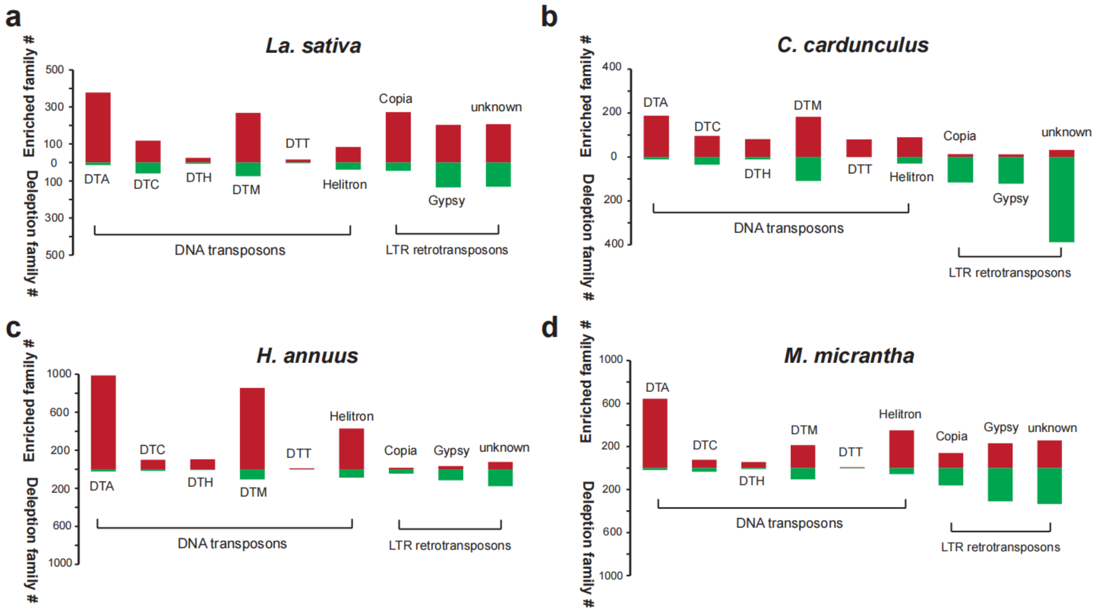

Supplementary Fig. 21. Enrichment and depletion analyses of repeat element families in the triplication-retained regions of four selected species in Asteraceae.

a, *Lactuca sativa* var. *angustana*. **b**, *Cynara cardunculus* var. *scolymus*. **c**, *Helianthus annuus*. **d**, *Mikania micrantha*. A one-sided Fisher's test was adopted to conducted the Enrichment and depletion analyses of repeat element families and adjustments were made for multiple comparisons using Benjamini–Hochberg Method. Source data are provided as a Source Data file.

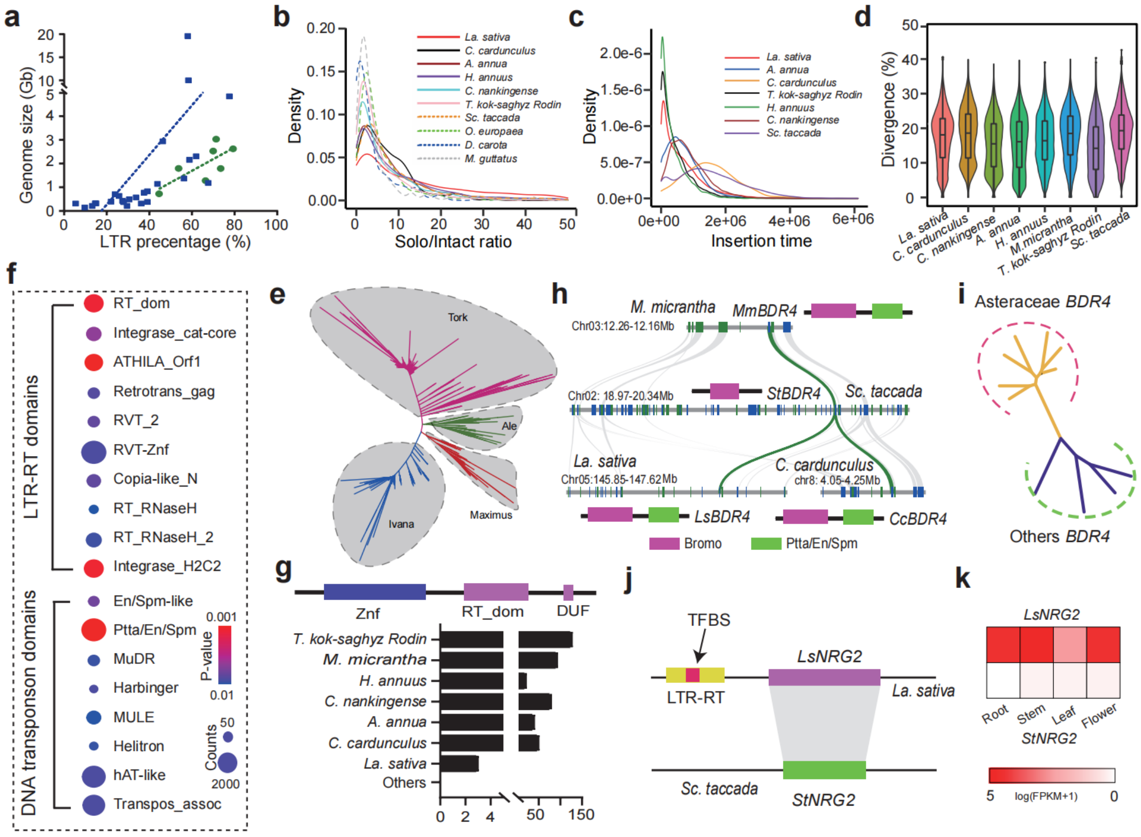

Supplementary Fig. 22. Diversification and dynamics of (retro)transposons from Asteraceae genomes.

a, Correlation between genome size and long terminal repeat (LTR) retrotransposons (R^2 = 0.74). Green and blue dots represent Asteraceae species and others, respectively, while green and blue lines are their corresponding best linear fits. **b**, Density distribution of solo/intact ratio of LTR retrotransposons in the representative Asteraceae and phylogenetic relatives. Solid lines represent Asteraceae genomes, while dashed lines represent others. **c**, Density distribution of insertion time of LTR retrotransposons in the Asteraceae species and *Scaevola taccada*. **d**, Sequence divergence distribution patterns of transposable element (TE) hits presented as a violin plot. The most recent LTR retrotransposon sequences of LTR retrotransposon families were selected as representative sequences to detect additional TE hits in the genomes. In violin plots, central line: median values; bounds of box: 25th and 75th percentiles; whiskers: 1.5 * IQR (IQR: the interquartile range between the 25th and 75th percentile). The number (n) of data points for each violin plot is shown below. **e**, Phylogenetic analysis of the LTR retrotransposon sequences (*Ty1*/*Copia*) in the *Lactuca sativa* genome. The maximum-likelihood and unrooted phylogenetic trees were constructed based on 1,564 *Ty1*/*Copia* aligned sequences that corresponded to the reverse transcriptase domain without premature termination codon. **f**, Enriched (retro)transposon-related InterPro entries in the Asteraceae species. An enrichment analysis was performed based on the functional domains of all the genes across the 29 surveyed species. *P* values were derived from a one-sided hypergeometric test with Bonferroni correction. **g**, A typical high-copy lineage-specific gene family with retrotransposon-related domains in Asteraceae. **h**, Micro-synteny visualization of *BDR4* syntelogs harboring transposon-related domains in Asteraceae species but not in *Sc. taccada*. **i**, Phylogenetic

tree of the transposon-related *BDR4* syntelogs in the Asteraceae species, *Sc. taccada*, *Vitis vinifera*, *Coffea arabica*, and *Arabidopsis thaliana*. **j, k,** An example of transcription factor binding site (TFBS) gain by the insertion of LTR retrotransposons. The TFBS in *LsNRG2* of *La. sativa* was possibly introduced by a LTR retrotransposon after speciation from *Sc. taccada* (**j**) and caused significantly different expression profiles among species (**k**). Source data are provided as a Source Data file.

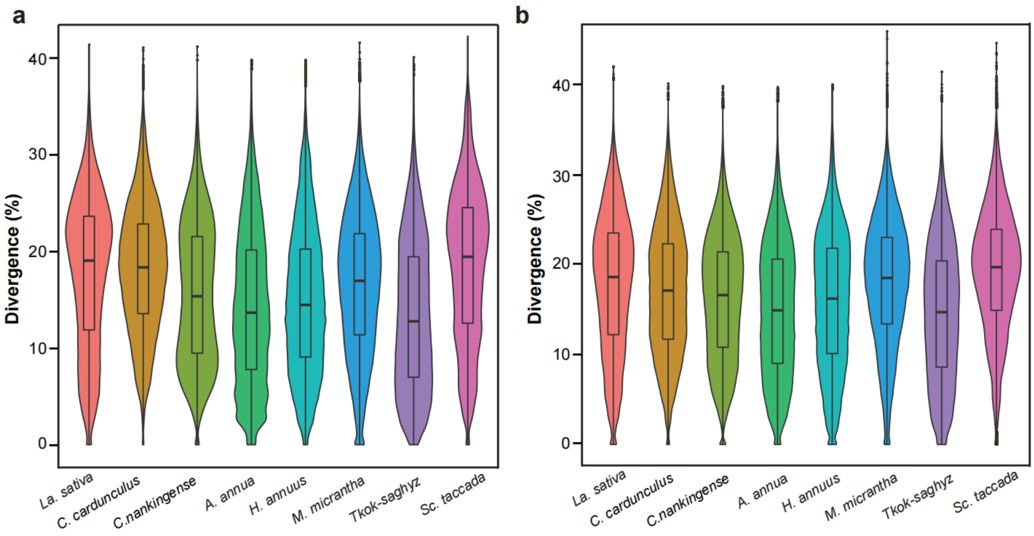

Supplementary Fig. 23. Divergence of different types of long terminal repeat retrotransposons (LTR-RTs) in the Asteraceae family and *Scaevola taccada*.

a, Violin plots of the sequence divergence of *Gypsy*-type LTR-RTs in the Asteraceae family and *Sc. taccada*. **b**, Violin plots of the sequence divergence of unclassified-type LTR-RTs in the Asteraceae family and *Sc. taccada*. In violin plots, central line: median values; bounds of box: 25th and 75th percentiles; whiskers: 1.5 * IQR (IQR: the interquartile range between the 25th and 75th percentile). Source data are provided as a Source Data file.

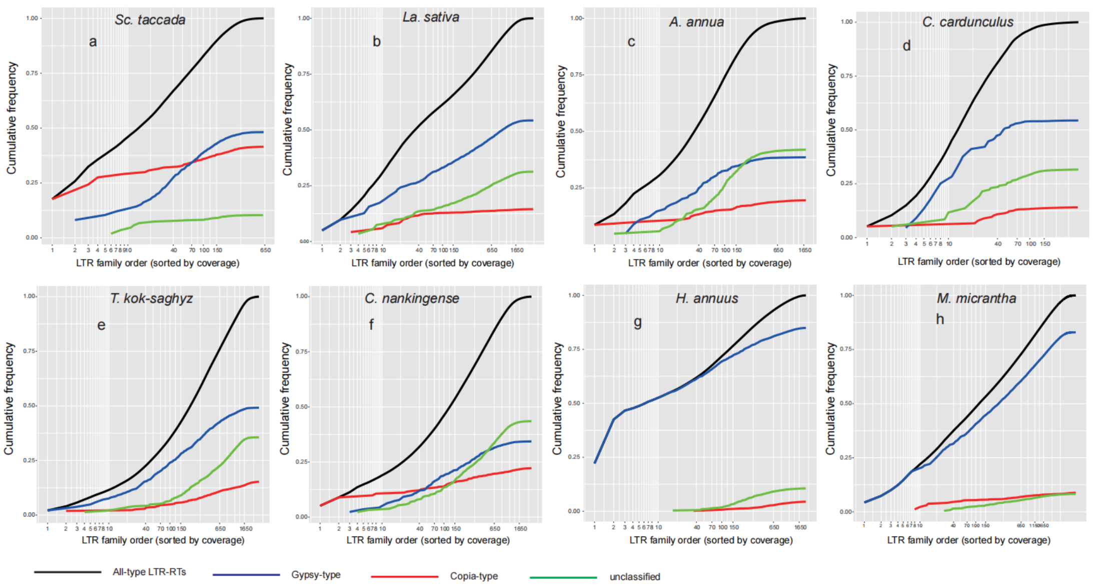

Supplementary Fig. 24. Long terminal repeat retrotransposon coverage based on family order in *Scaevola taccada* and the seven Asteraceae species.

a, *Scaevola taccada*. **b**, *Lactuca sativa* var. *angustana*. **c**, *Artemisia annua*. **d**, *Cynara cardunculus* var. *scolymus*. **e**, *Taraxacum kok-saghyz Rodin*. **f**, *Chrysanthemum nankingense*. **g**, *Helianthus annuus*. **h**, *Mikania micrantha*. Source data are provided as a Source Data file.

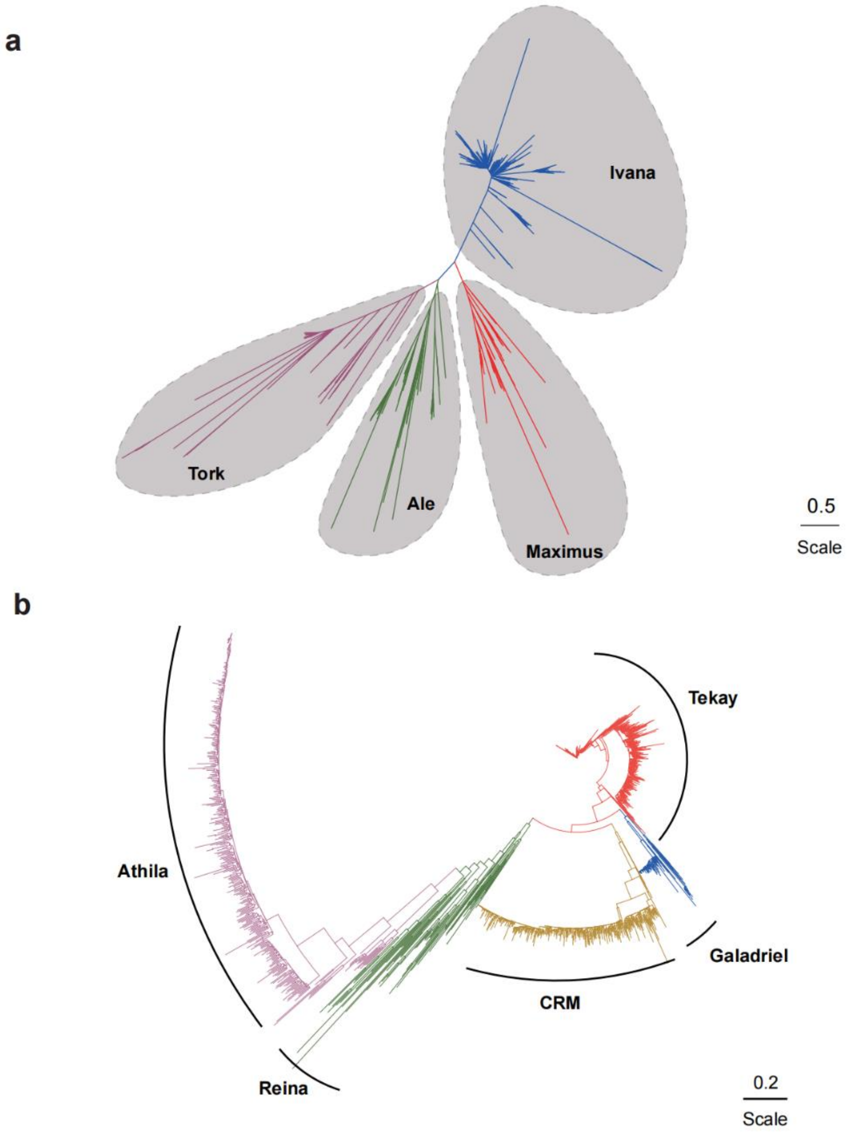

Supplementary Fig. 25. Phylogenetic analysis of the long terminal repeat (LTR) retrotransposon (RT) sequences in the *Scaevola taccada* genome.

The maximum-likelihood and unrooted phylogenetic trees were constructed on the basis of 873 *Ty1*/*Copia* (**a**) and 2,009 *Ty3*/*Gypsy* (**b**) aligned sequences corresponding to the RT domains without premature termination codon. LTR family names and the proportion of each are indicated.

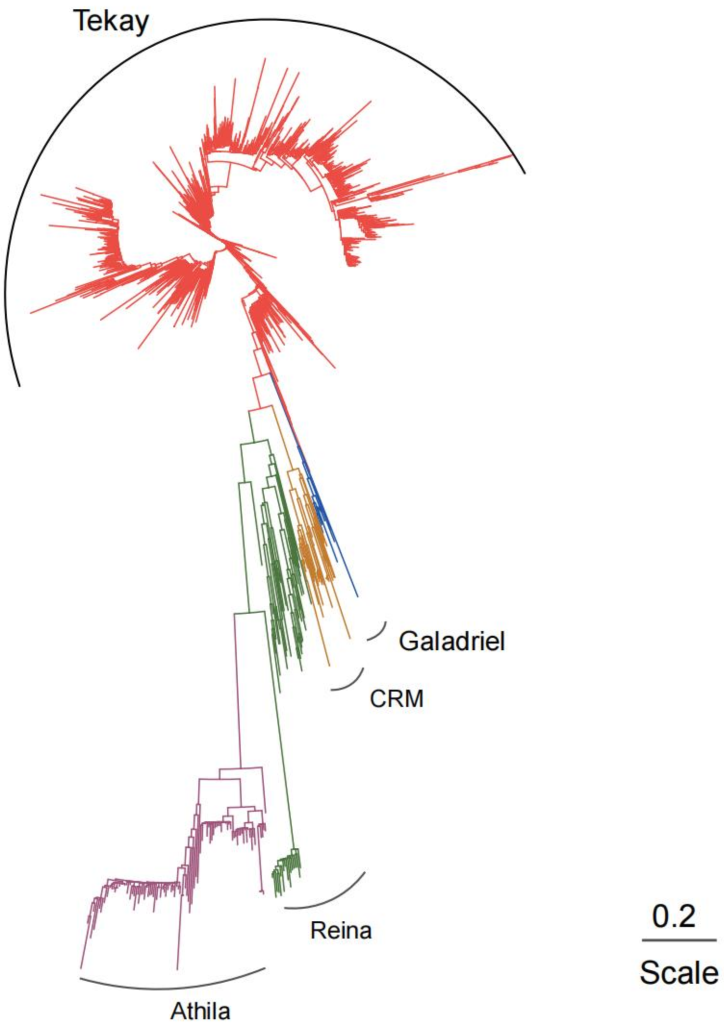

Supplementary Fig. 26. Phylogenetic analysis of the long terminal repeat (LTR) retrotransposon (RT) sequences (*Ty3*/*Gypsy*) in the *Lactuca sativa* var. *angustana* genome. The maximum-likelihood and unrooted phylogenetic trees were constructed on the basis of 2,301 *Ty3*/*Gypsy* aligned sequences corresponding to the RT domains without a premature termination codon. For better visualization, the 10,093 intact *Ty3*/*Gypsy* LTR-RTs were clustered using CD-HIT-EST with the parameters '-c 0.8 -aL 0.8 -T 0 -M 0 -n 5'. LTR family names and the proportion of each are indicated.

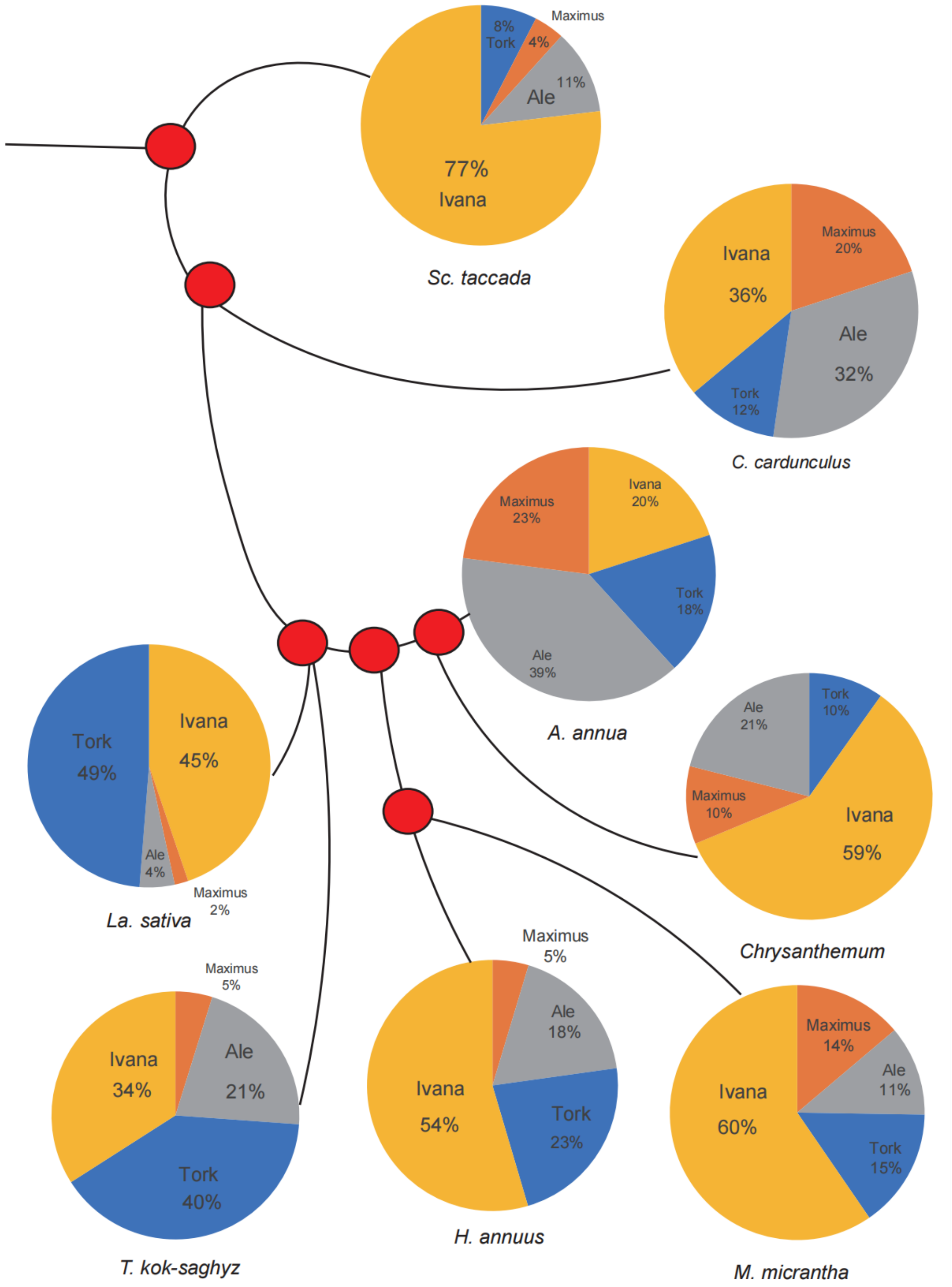

Supplementary Fig. 27. Distribution of *Copia* families in seven Asteraceae species and *Scaevola taccada*.

Red circles indicate the phylogenic relationship among these species. The seven Asteraceae species are *Lactuca sativa* var. *angustana*, *Cynara cardunculus* var. *scolymus*, *Helianthus annuus*, *Chrysanthemum nankingense*, *Artemisia annua*, *Taraxacum kok-saghyz* Rodin, and *Mikania micrantha*.

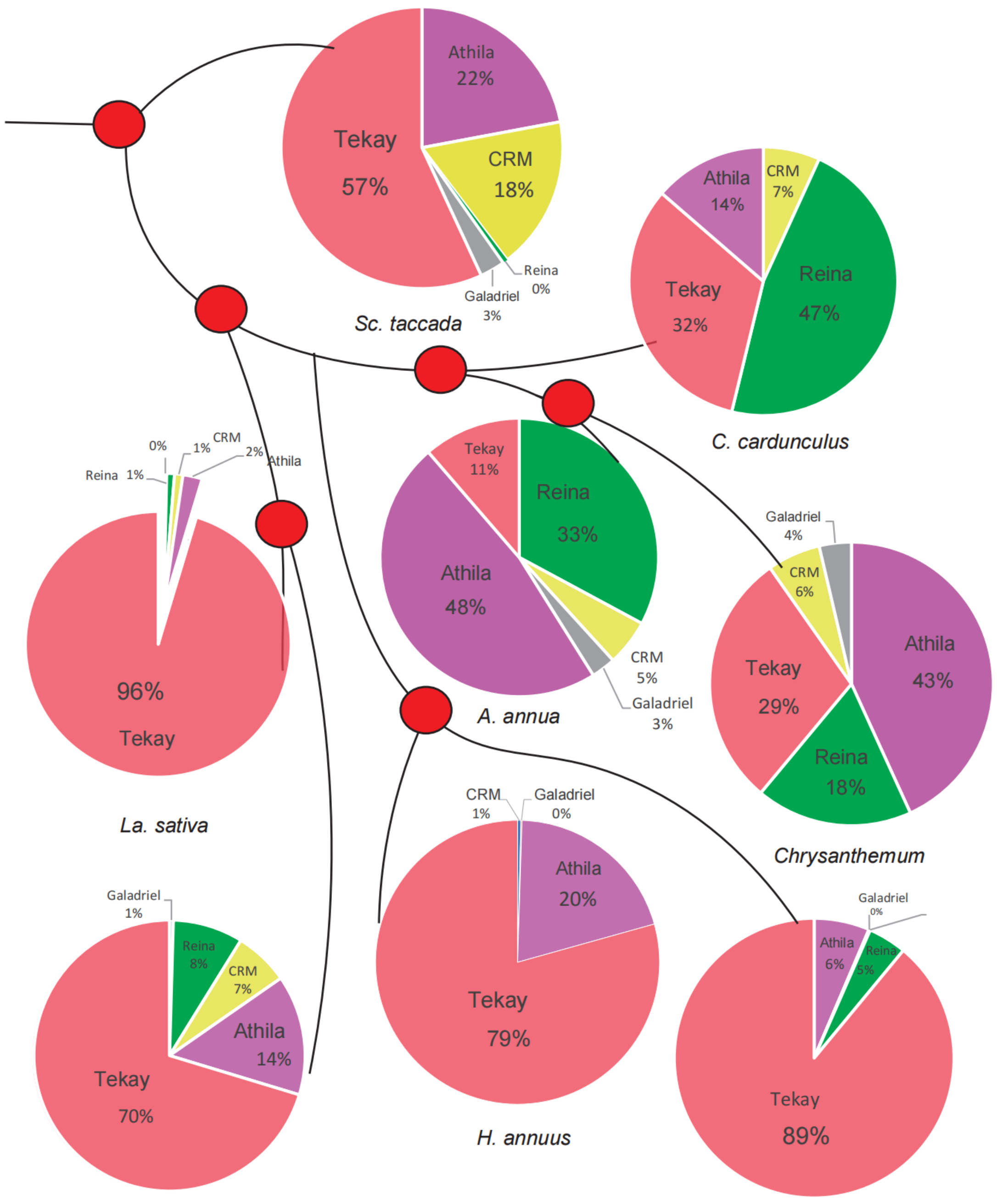

Supplementary Fig. 28. Distribution of *Gypsy* families in seven selected Asteraceae species and *Scaevola taccada*.

Red circles indicate the phylogenic relationship among these species. The seven Asteraceae species are *Lactuca sativa* var. *angustana*, *Cynara cardunculus* var. *scolymus*, *Helianthus annuus*, *Chrysanthemum nankingense*, *Artemisia annua*, *Taraxacum kok-saghyz* Rodin, and *Mikania micrantha*.

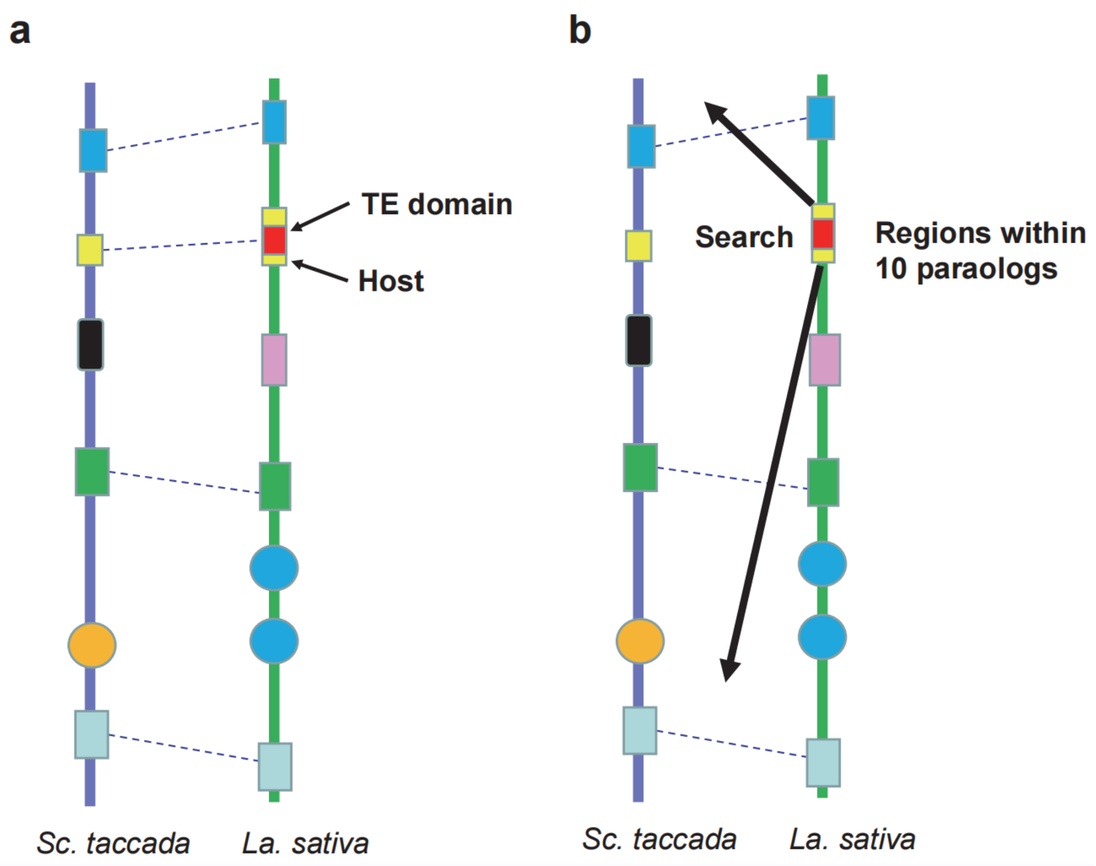

Supplementary Fig. 29. Schematic diagram of identification of (retro)transposon element host genes after speciation.

a, Pairwise synteny was generated first by comparing the *Lactuca sativa* var. *angustana* genome to the *Scaevola taccada* genome. Functional domain analysis was performed between the pairwise paralogs, and the repetitive element domains identified in *La. sativa* but not in *Sc. taccada* were considered to be introduced by insertion of a repetitive element. **b**, Pairwise synteny was generated first by comparing the *La. sativa* genome to the *Sc. taccada* genome. We searched candidate homologous genes with the (retro)transposon element host genes in *La. sativa* within nearby genomic regions. The hit gene without a repetitive element domain was considered to be the homolog of the host gene. TE, transposable element.

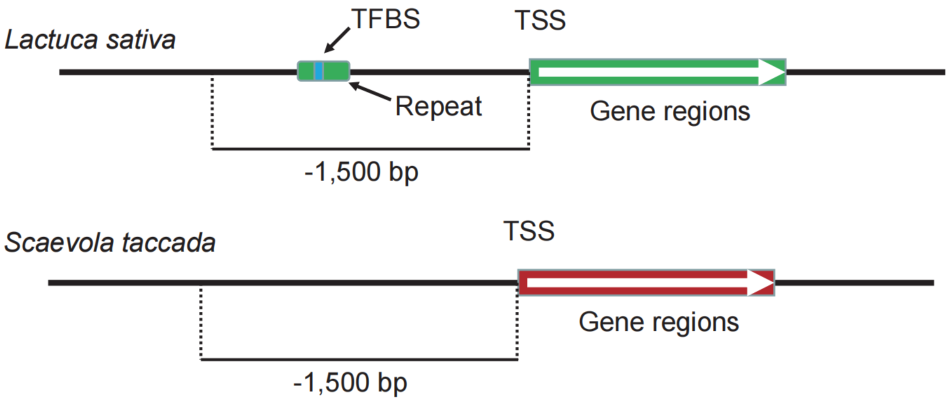

Supplementary Fig. 30. Schematic diagram for the identification of transcription factor binding sites (TFBSs) possibly introduced by repeat sequences after speciation.
TSS, transcription start site.

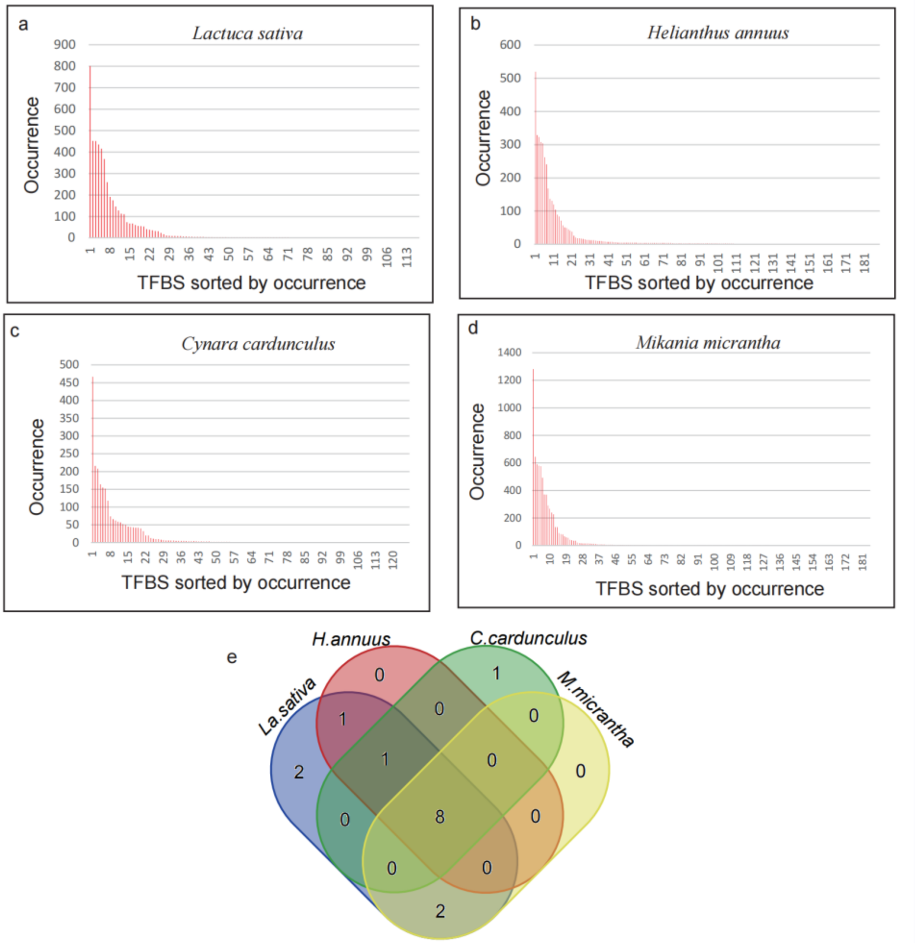

Supplementary Fig. 31. Occurrence of transcription factor binding sites (TFBSs) introduced by repeat sequences in the Asteraceae family.

a, *Lactuca sativa* var. *angustana*. **b**, *Helianthus annuus*. **c**, *Cynara cardunculus* var. *scolymus*. **d**, *Mikania micrantha*. **e**, Venn diagram of the TFBSs with top 10 occurrence in each species. Source data are provided as a Source Data file.

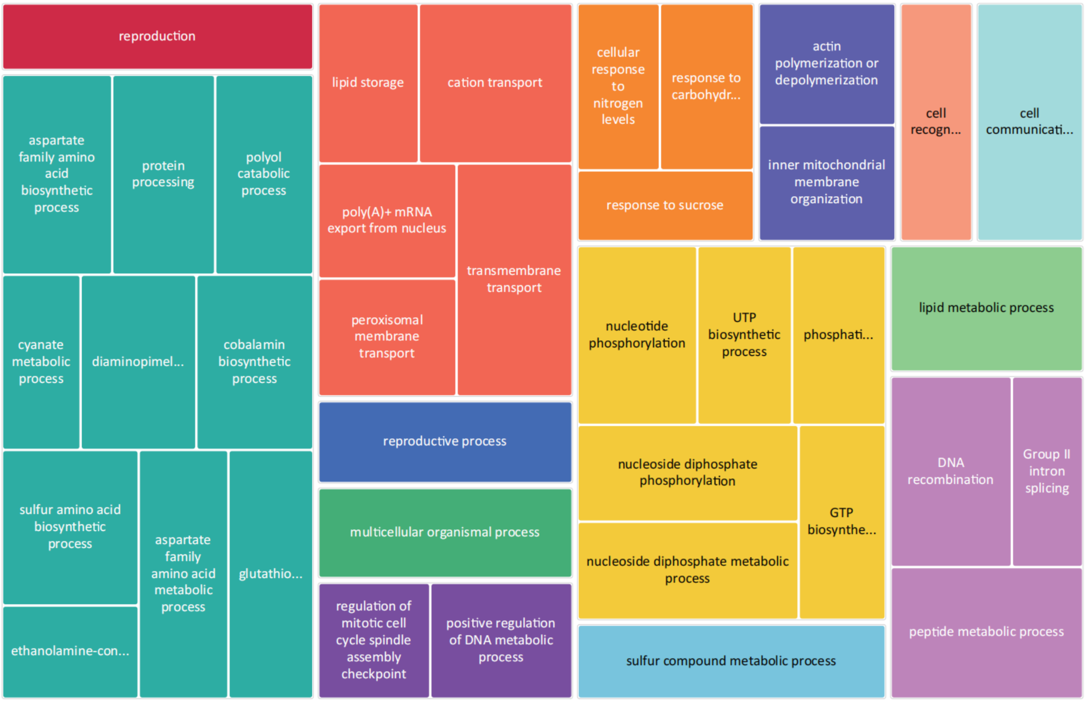

Supplementary Fig. 32. Enriched Gene Ontology terms of the genes possibly affected by the transcription factor binding sites introduced by repeat sequences in the *Lactuca sativa* var. *angustana* genome.

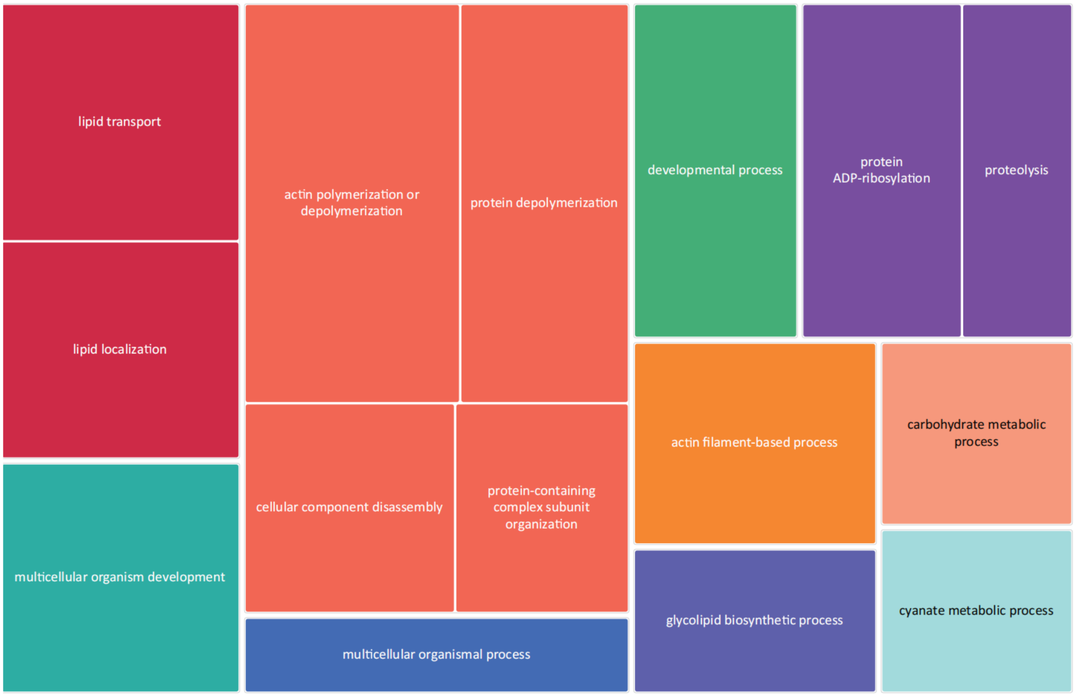

Supplementary Fig. 33. Enriched Gene Ontology terms of the genes possibly affected by the transcription factor binding sites introduced by repeat sequences in the *Cynara cardunculus* var. *scolymus* genome.

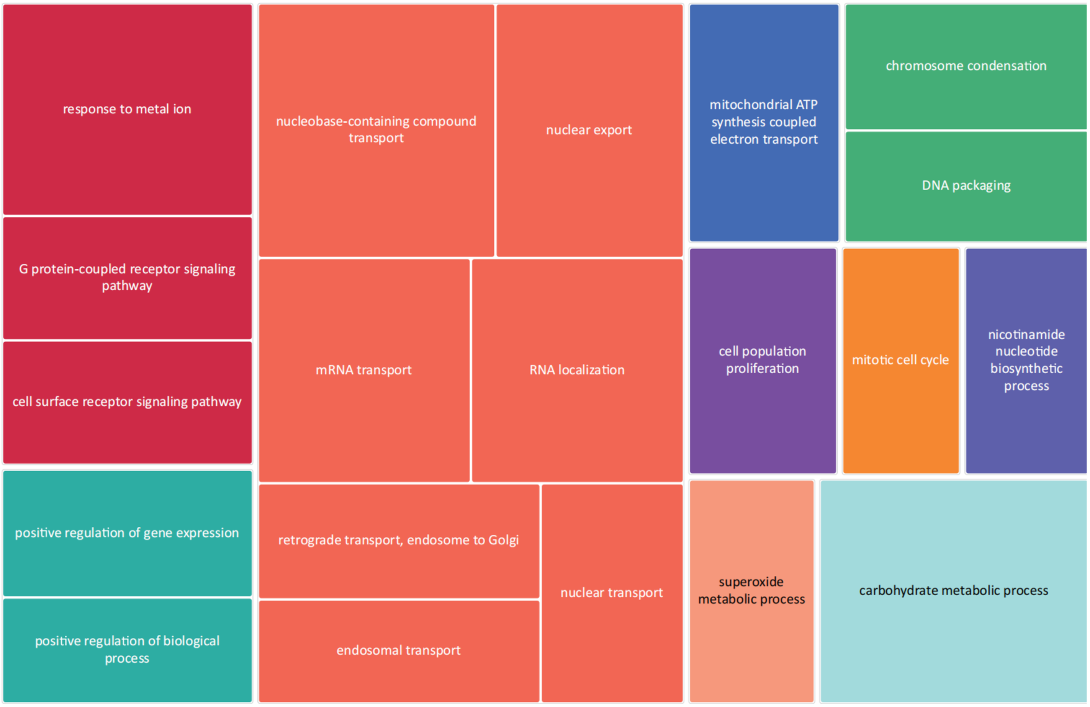

Supplementary Fig. 34. Enriched Gene Ontology terms of the genes possibly affected by the transcription factor binding sites introduced by repeat sequences in the *Helianthus annuus* genome.

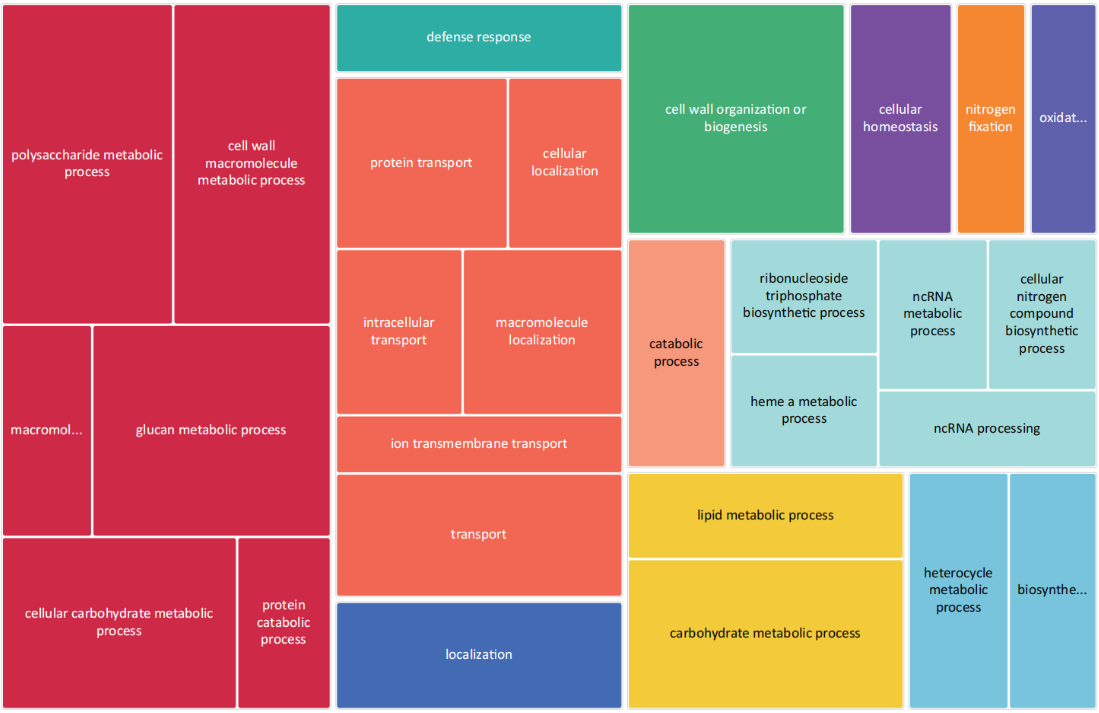

Supplementary Fig. 35. Enriched Gene Ontology terms of the genes possibly affected by the transcription factor binding sites introduced by repeat sequences in the *Mikania micrantha* genome.

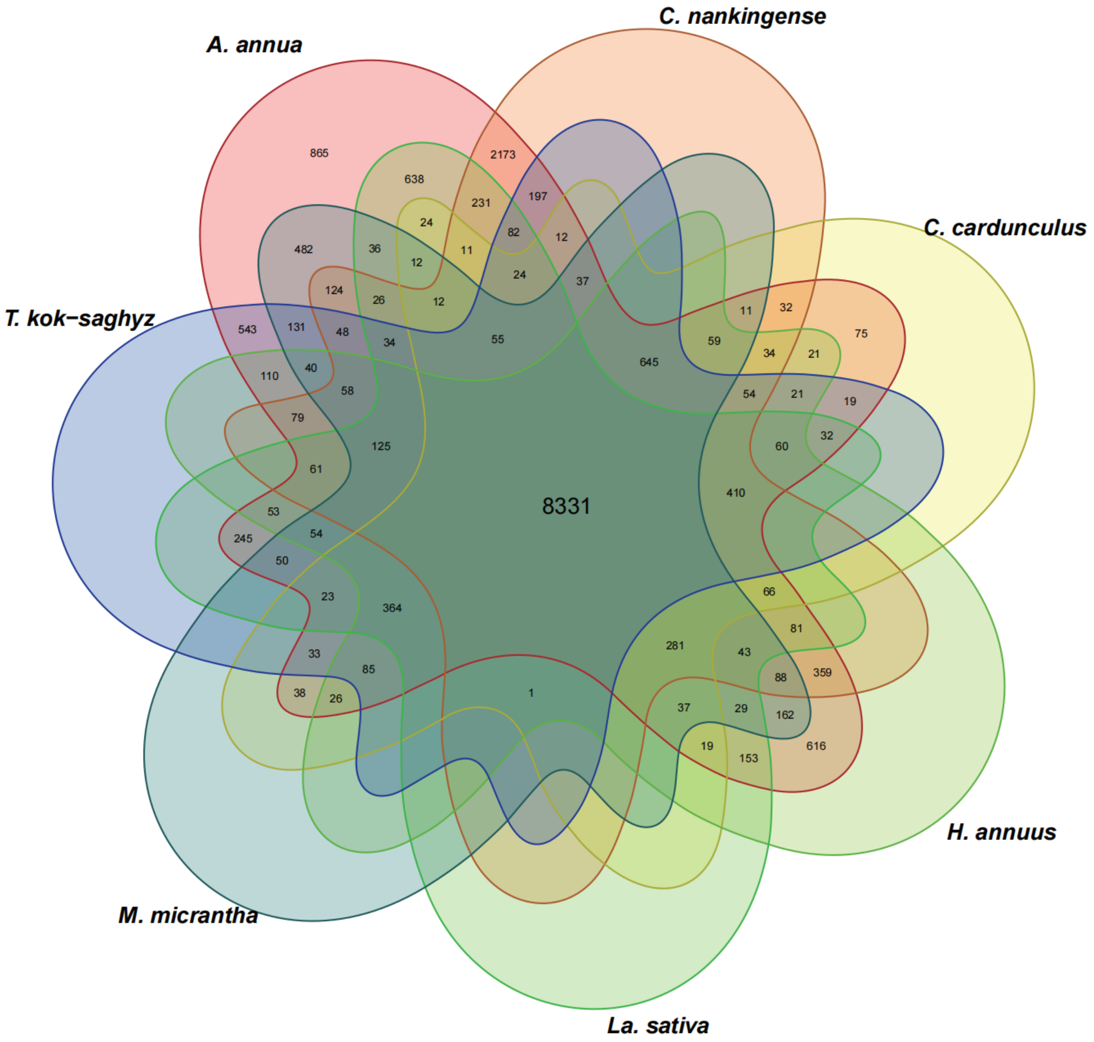

Supplementary Fig. 36. Venn diagram of orthologous groups across the seven Asteraceae species *Lactuca sativa* var. *angustana*, *Cynara cardunculus* var. *scolymus*, *Helianthus annuus*, *Chrysanthemum nankingense*, *Artemisia annua*, *Taraxacum kok-saghyz* Rodin, and *Mikania micrantha*.

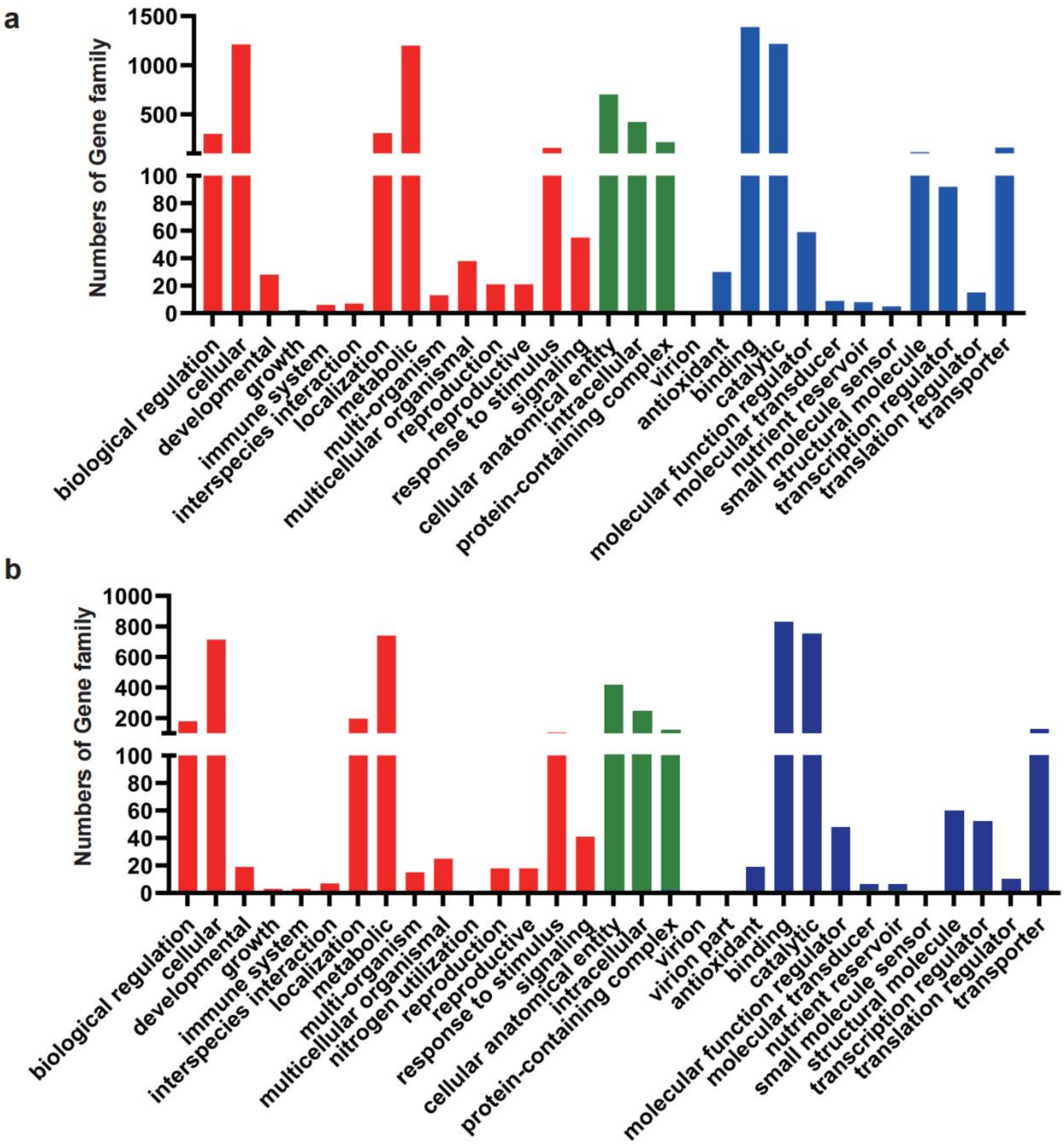

Supplementary Fig. 37. Gene Ontology (GO) classification of significantly expanded gene families at the crown node of the Asteraceae species (a) or at the crown node of Asteraceae and *Scaevola taccada* (b).

GO terms in molecular function (blue), biological process (red), and cellular component (green) categories are indicated in different colors.

Source data are provided as a Source Data file.

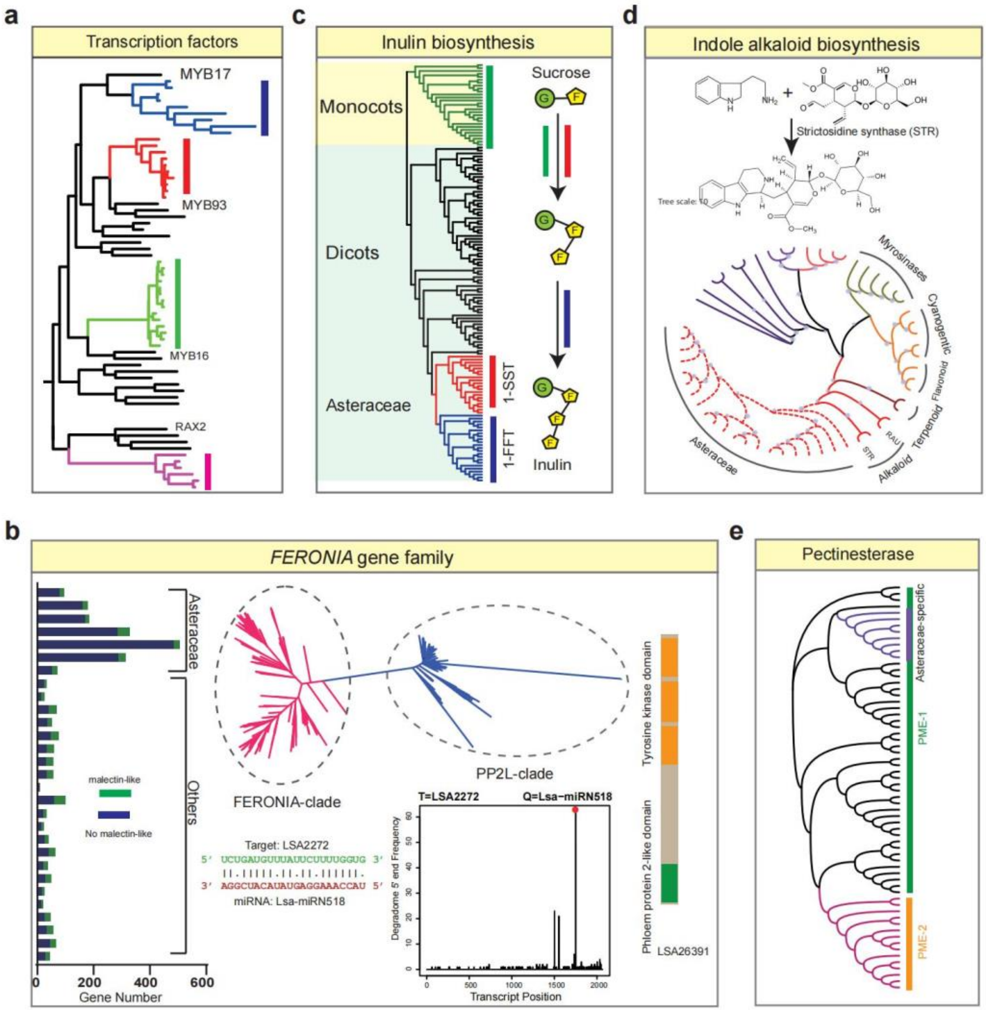

Supplementary Fig. 38. Evolution of representative gene families related to the characteristics of the Asteraceae. a, Divergence of the *R2R3-MYB* transcription factor gene family in Asteraceae. Asteraceae-specific clades are marked with coloured lines. **b**, The *FERONIA* family in Asteraceae. The bar chart illustrates the number of genes for each *FERONIA* type (with or without the typical malectin-like domain) in all 29 investigated species. The phylogenetic tree includes the typical *FERONIA* genes and Asteraceae-specific clades with the phloem protein 2-like (PP2L) domain. The unique miRNA-target pair (Asteraceae-specific miRN518 and *LSA2272,* a representative *FERONIA* gene lacking the sequence encoding the malectin-like domain) are illustrated by sequence alignment. This regulatory pair was supported by a parallel analysis of RNA ends (PARE)-seq experiment. **c**, The simplified phylogenetic tree of Glycosyl hydrolase family 32 (GH32) protein in different species and schematic diagram of inulin biosynthesis. For the phylogenetic analysis, we used genes from the 29 investigated species in this study and genes encoding sucrose:sucrose 1-fructosyltransferase (1-SST), fructan:fructan 1-fructosyltransferase (1-FFT), and fructan 1-exohydrolases (FEHs) previously identified in chicory (*Cichorium intybus*) and barley (*Hordeum vulgare*). The unique 1-SST and 1-FFT clades in Asteraceae species are shown in red and blue, respectively. **d**, Schematic diagram of the role of strictosidine synthase (STR) in indole alkaloid biosynthesis and the phylogenetic tree of identified genes of the *Beta-glucosidase gene family 1* in different species. The Asteraceae-specific clade is illustrated by dotted lines. **e**, The simplified phylogenetic tree of typical genes encoding pectinesterases highlighting Asteraceae-specific clades (purple lines). Source data are provided as a Source Data file.

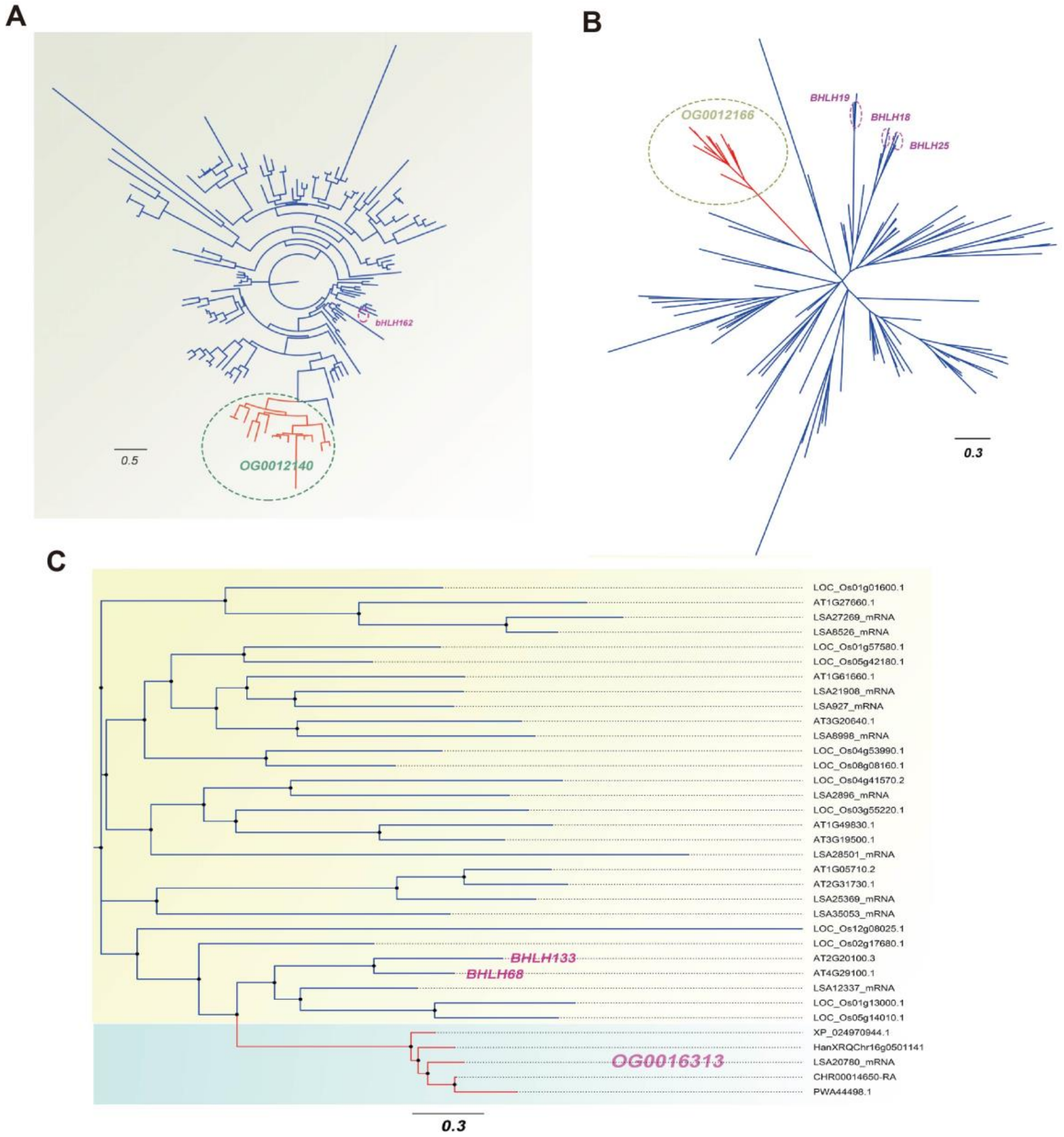

Supplementary Fig. 39. Phylogenetic tree of Asteraceae lineage-specific *bHLH* gene families. Lineage-specific gene families, harboring 35 genes in the Asteraceae family, were identified. A phylogenetic analysis using genes of each Asteraceae lineage-specific family and its closest related family was conducted. The transcription factor genes (**a**) *bHLH162*, (**b**) *bHLH19/18/25*, and (**c**) *bHLH68/133* were identified as the genes most similar to those in the three lineage-specific orthologous groups.

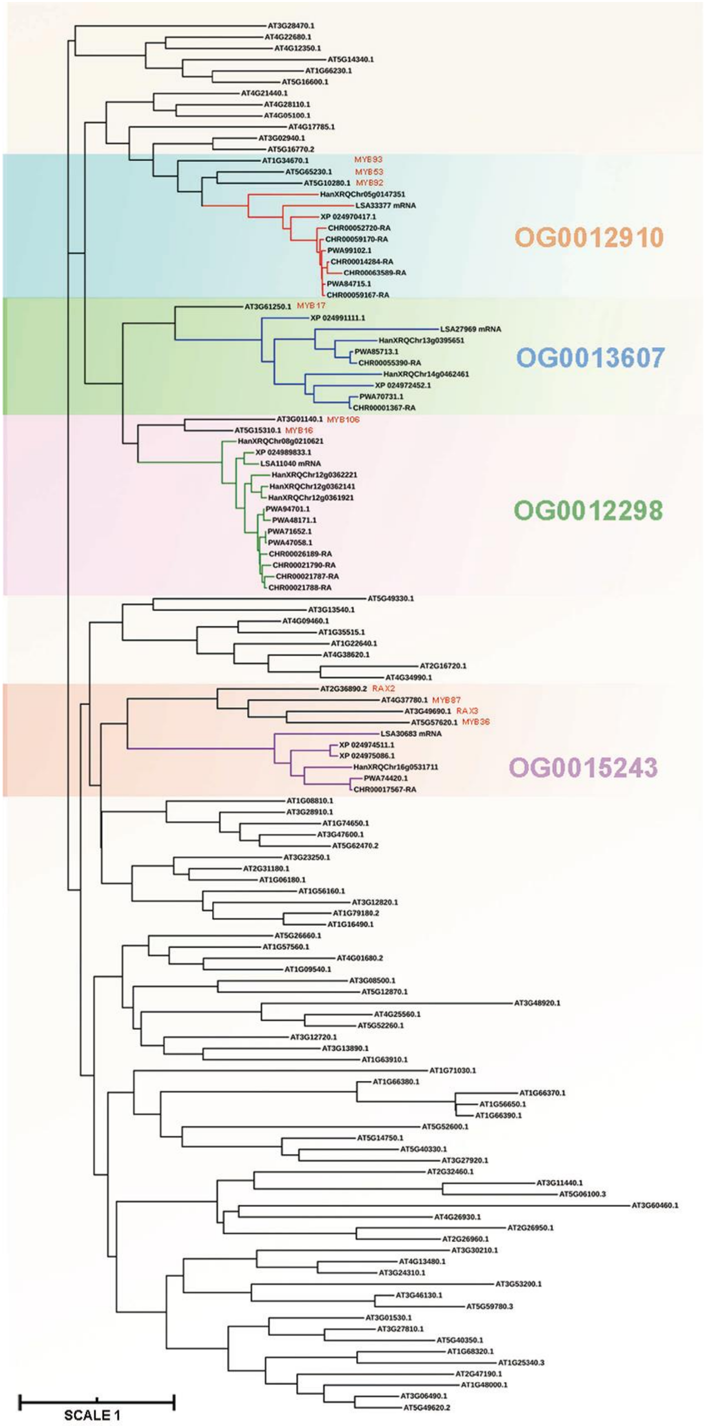

Supplementary Fig. 40. Phylogenetic tree of lineage-specific *R2R3-MYB* gene families in Asteraceae species.

A phylogenetic analysis using genes of each Asteraceae lineage-specific family and all *R2R3-MYB* genes of *Arabidopsis thaliana* was conducted to investigate homologous genes and potential function of specific clades.

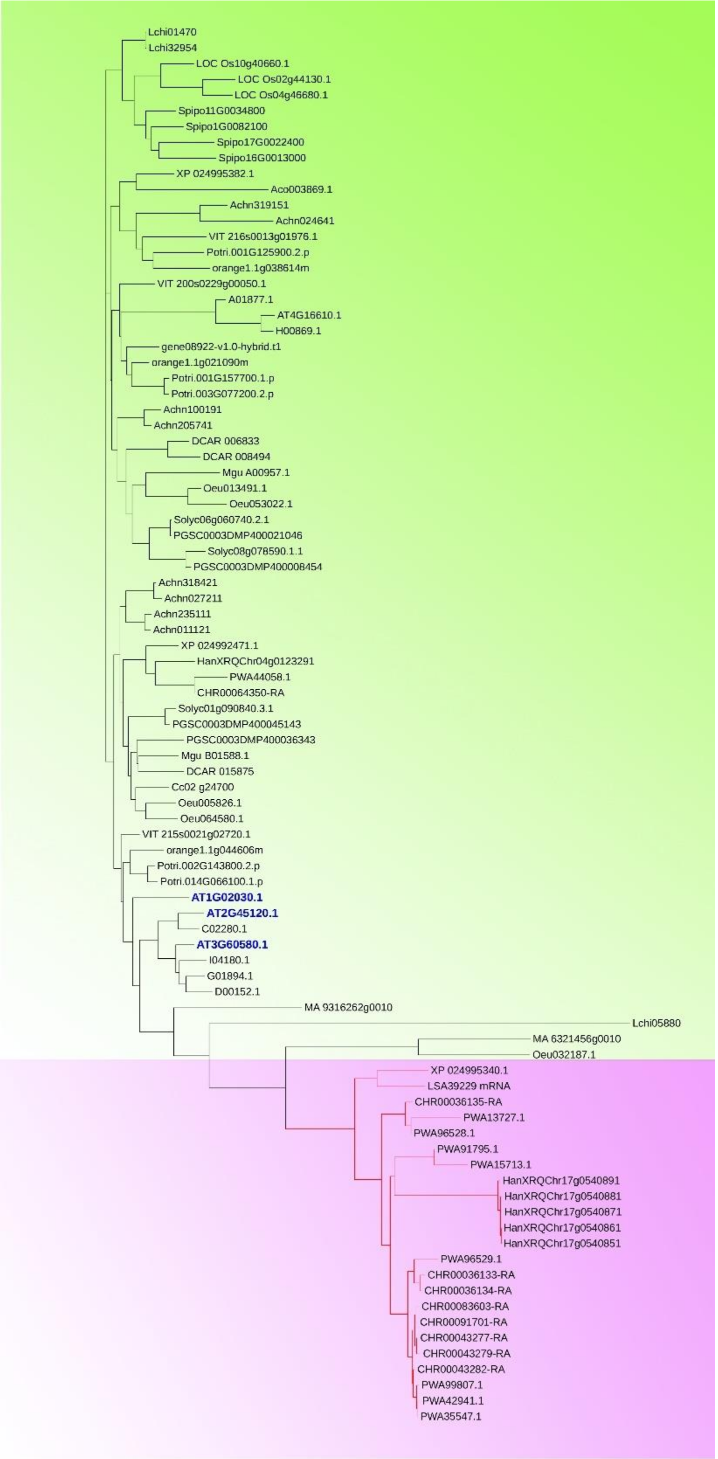

Supplementary Fig. 41. Phylogenetic tree lineage-specific zinc finger gene family of the Asteraceae species.

Clades with red color are specific to the Asteraceae lineage. Genes in blue font are from *Arabidopsis thaliana*.

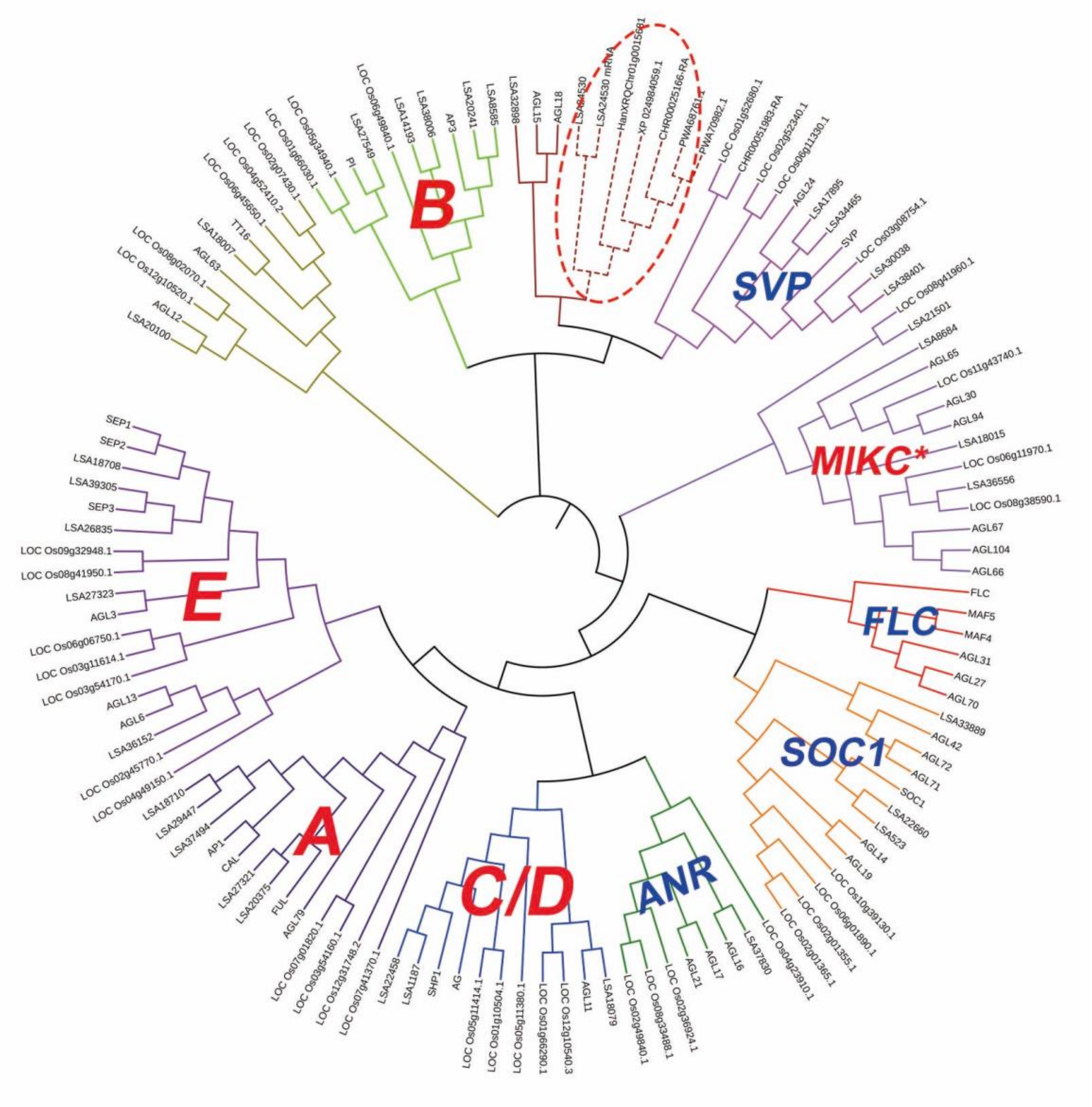

Supplementary Fig. 42. Phylogenetic tree of the Asteraceae lineage-specific MIKC-type MADS-box gene family.

Genes from *Arabidopsis thaliana*, *Oryza sativa* (gene names with LOC characters), and *Lactuca sativa* (gene names with LSA characters) are included, and those in the dashed oval are the Asteraceae-specific clades. Main clades are divided based on the gene function in Arabidopsis and are marked with different colors.

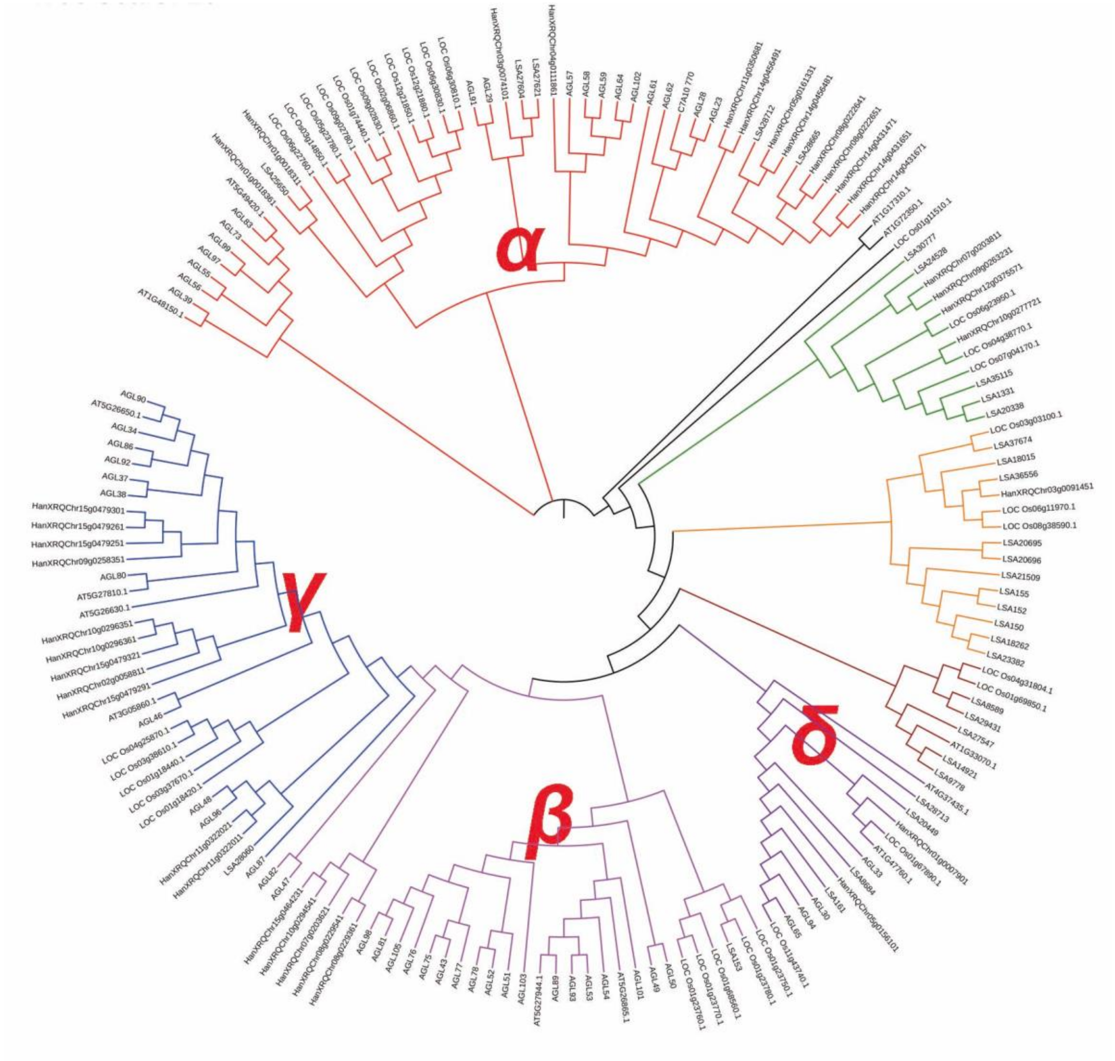

Supplementary Fig. 43. Phylogenetic tree of type I MADS-box genes in *Arabidopsis thaliana*, *Oryza sativa*, *Lactuca sativa,* and *Helianthus annuus.*

Genes from Arabidopsis, *O. sativa* (gene names with LOC characters), *La. sativa* (gene names with LSA characters), and *H. annuus* (gene names with Han characters) are included. Main clades are divided based on the gene function in Arabidopsis and gene functional domain analysis.

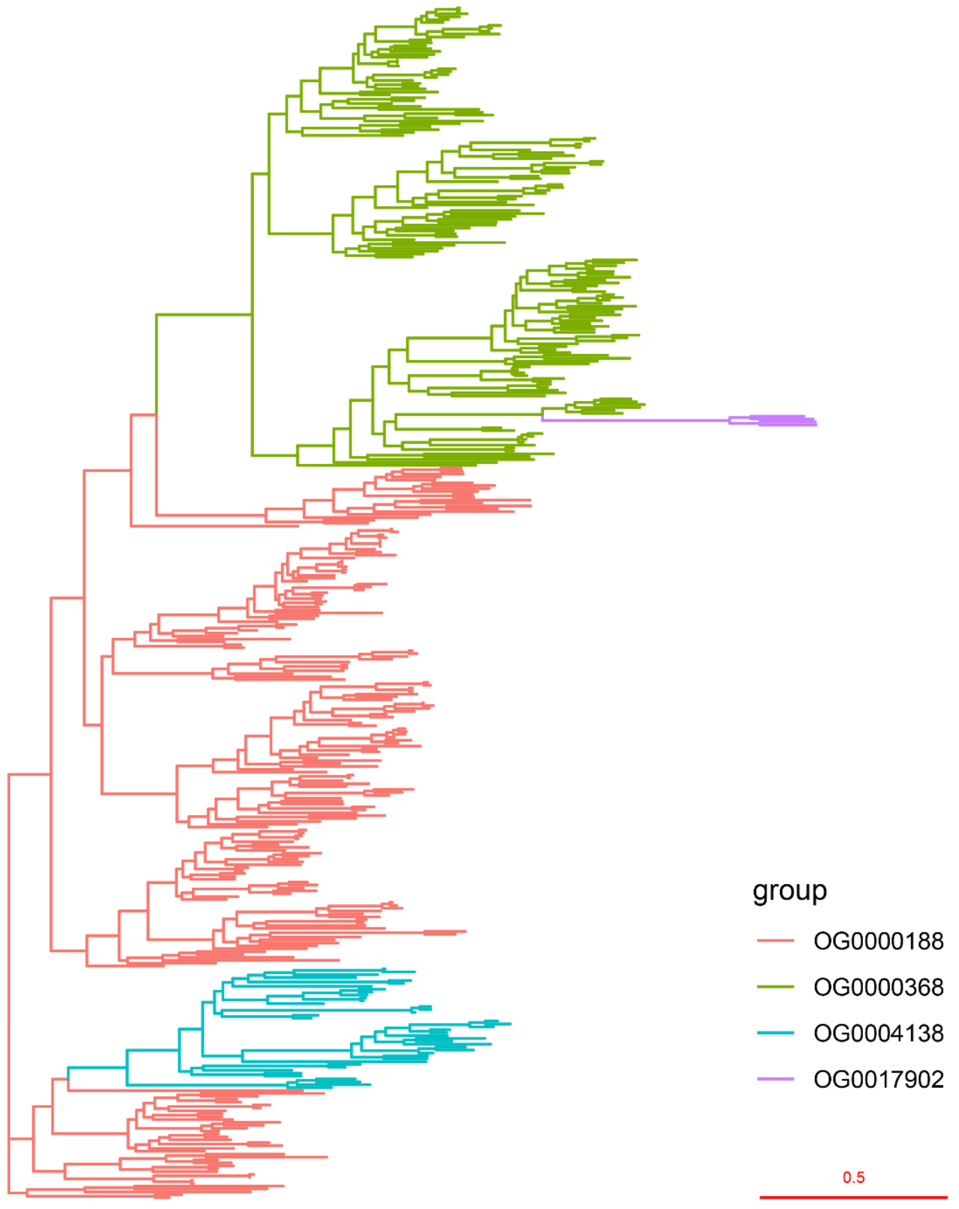

Supplementary Fig. 44. Phylogenetic tree of the FERONIA proteins from the 29 investigated species.

FERONIA and 16 closely related proteins form a distinct clade within *Arabidopsis thaliana* and are distributed in the four orthologous groups. The phylogenetic analysis was conducted with all genes in the four orthologous groups.

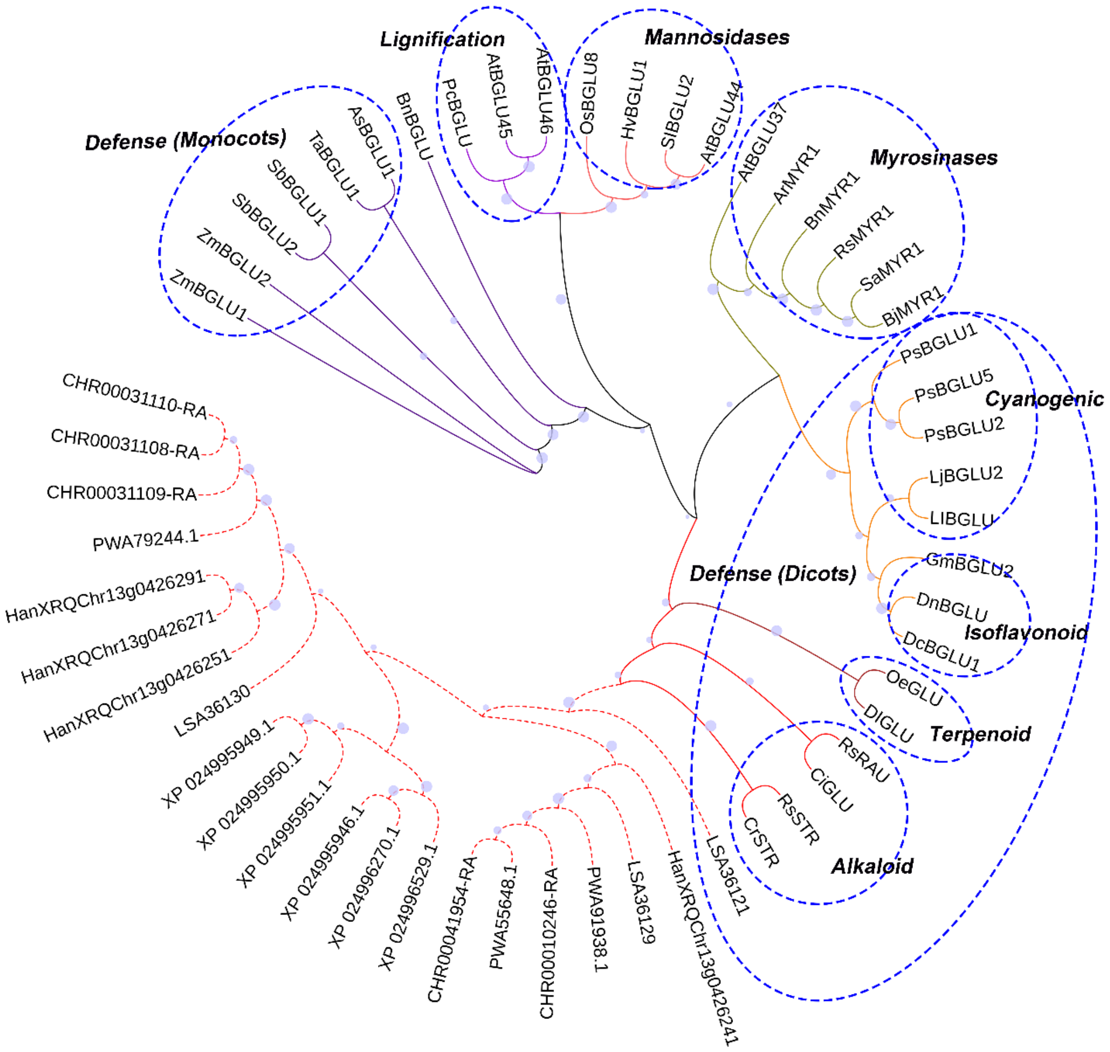

Supplementary Fig. 45. Phylogenetic tree of genes of the β-glucosidase gene family 1.
Phylogenetic tree of plant GH1 β-glucosidases retrieved from the CAZy database (http://www.cazy.org/). The enzymes are clustered according to their function or to the group of their specific substrates as indicated. Clades with dotted lines represent the Asteraceae-specific clades.

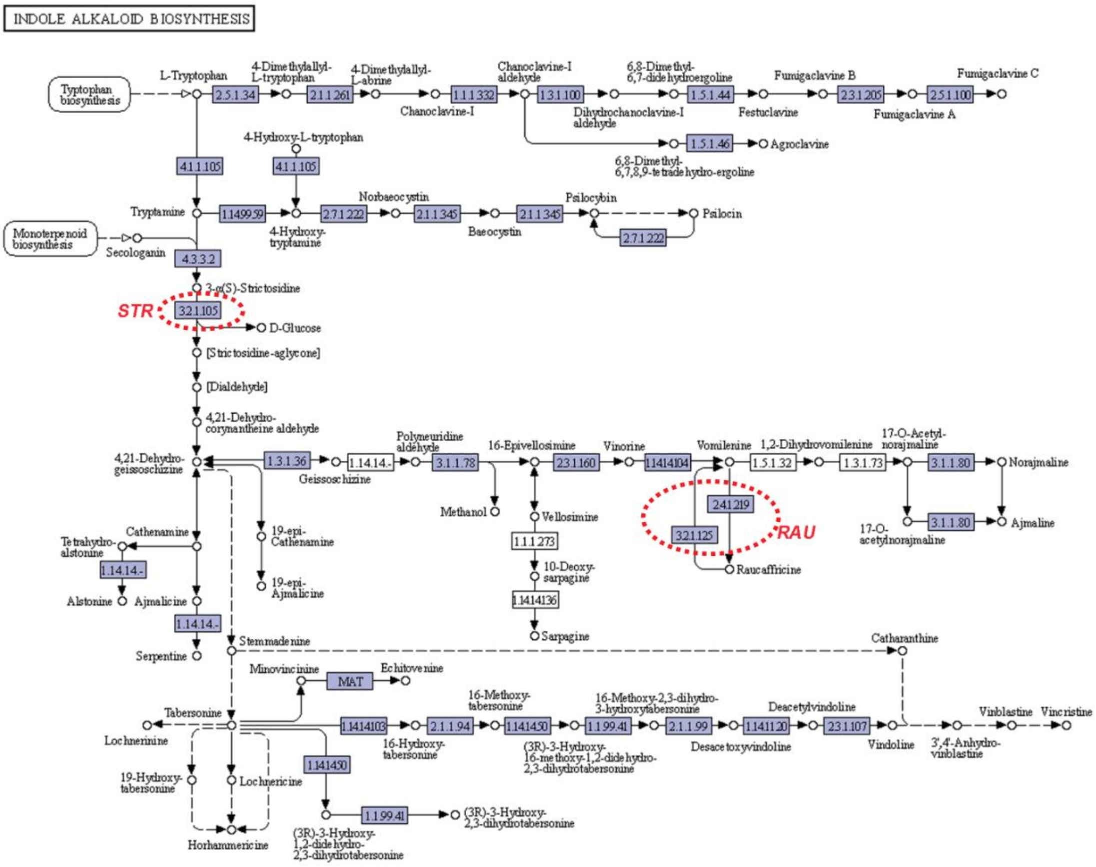

Supplementary Fig. 46. Metabolic diagram of indole alkaloid biosynthesis.

The original diagram was downloaded from the Kyoto Encyclopedia of Genes and Genomes (https://www.kegg.jp/) database. The metabolic positions of *STR* and *RAU* are marked.

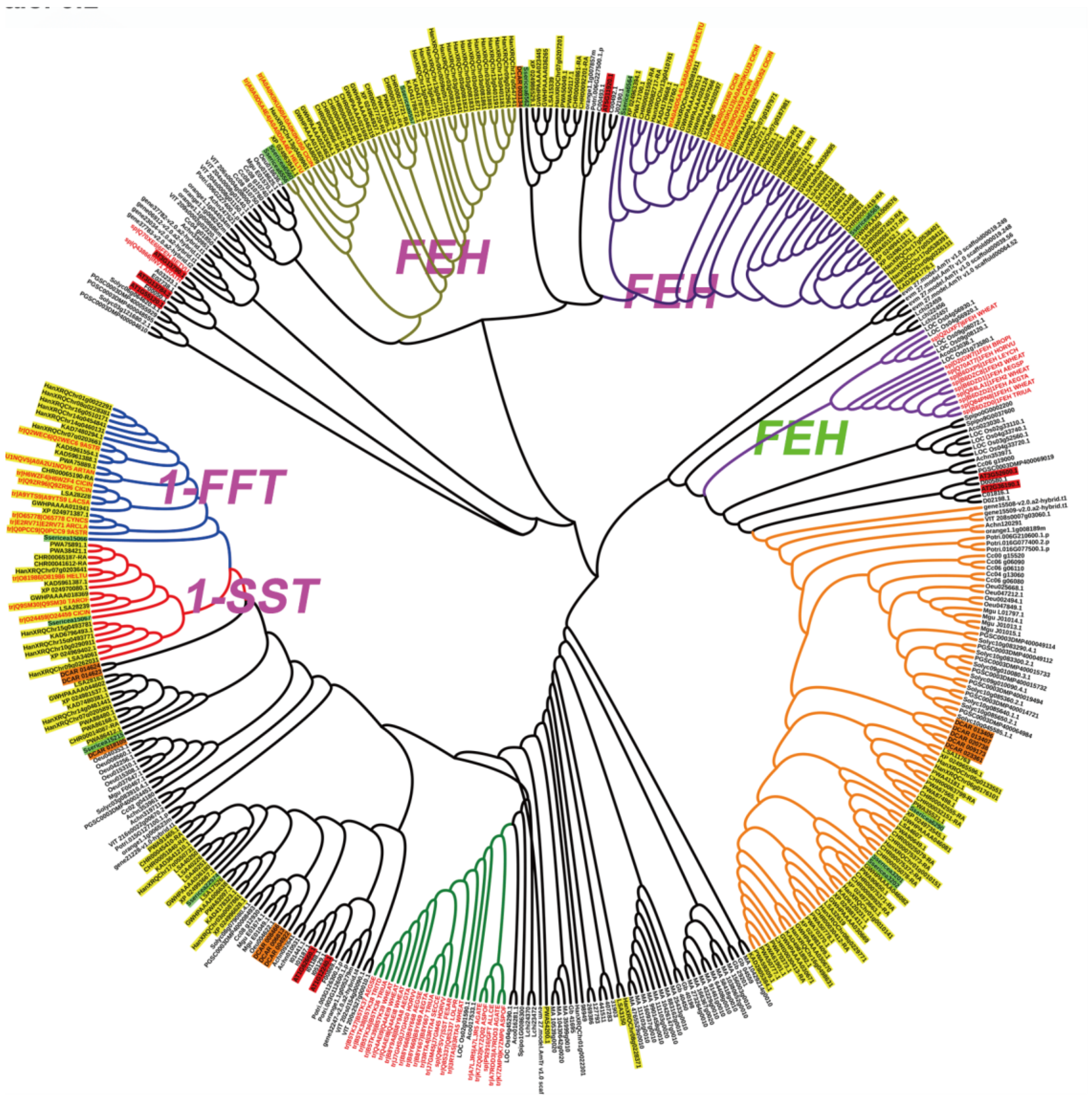

Supplementary Fig. 47. Phylogenetic tree of glycosyl hydrolase family 32 genes in different species.

Proteins from Asteraceae and *Arabidopsis thaliana* are shown with yellow and red backgrounds, respectively; blue clade: fructan:fructan 1-fructosyltransferase (1-FFT) proteins in Asteraceae; red clade: sucrose:sucrose 1-fructosyltransferase (1-SST) proteins in Asteraceae; green clade: SST/FFT proteins in monocots; brown clade: fructan exohydrolase I (FEH-I) proteins in Asteraceae; deep purple clade: fructan exohydrolase II (FEH-II) in Asteraceae; purple clade: FEH proteins in monocots.

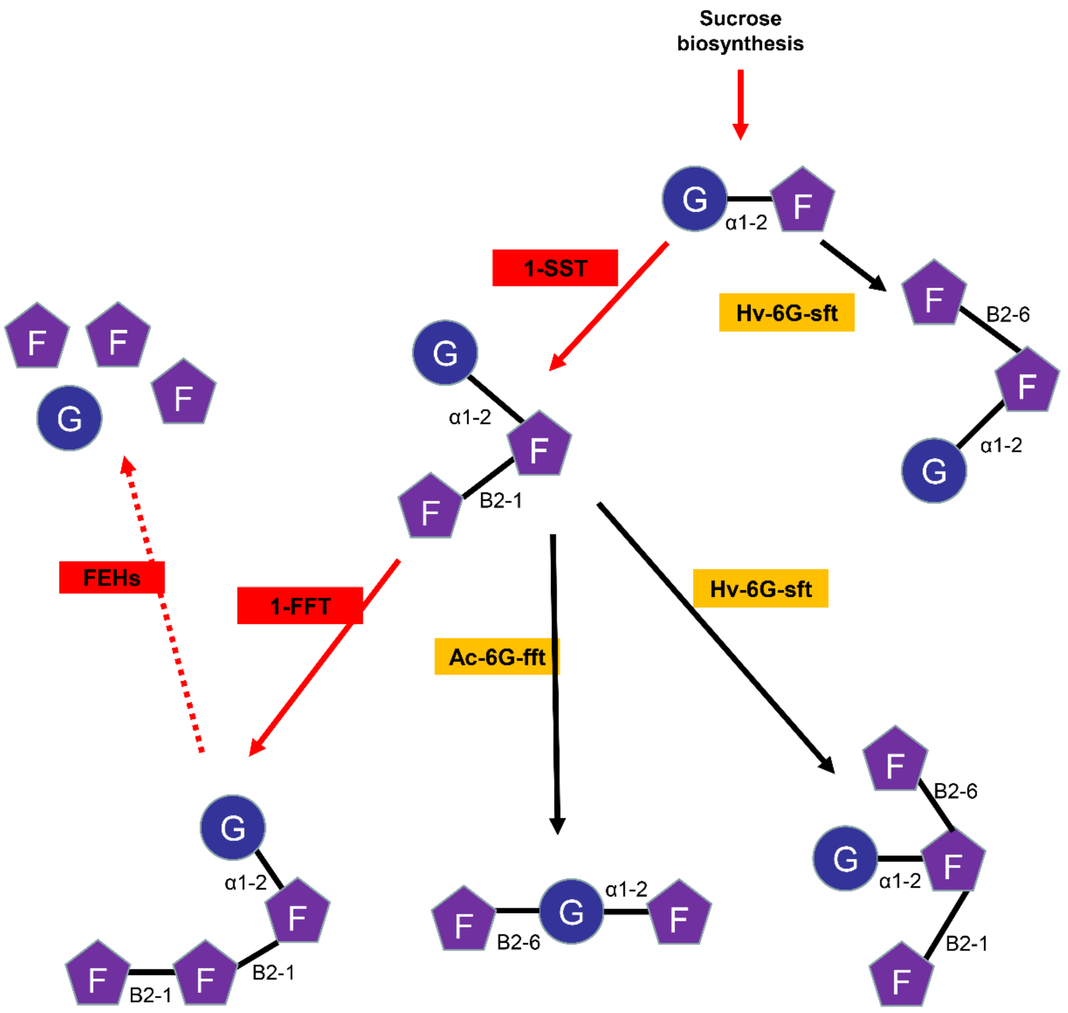

Supplementary Fig. 48. Schematic diagram of fructan biosynthesis and degradation in plants. 1-SST: sucrose:sucrose 1-fructosyltransferase; 1-FFT: fructan:fructan 1-fructosyltransferase; 6G-sft: sucrose:fructan 6-fructosyltransferase; 6G-fft: fructan:fructan 6G-fructosyltransferase.

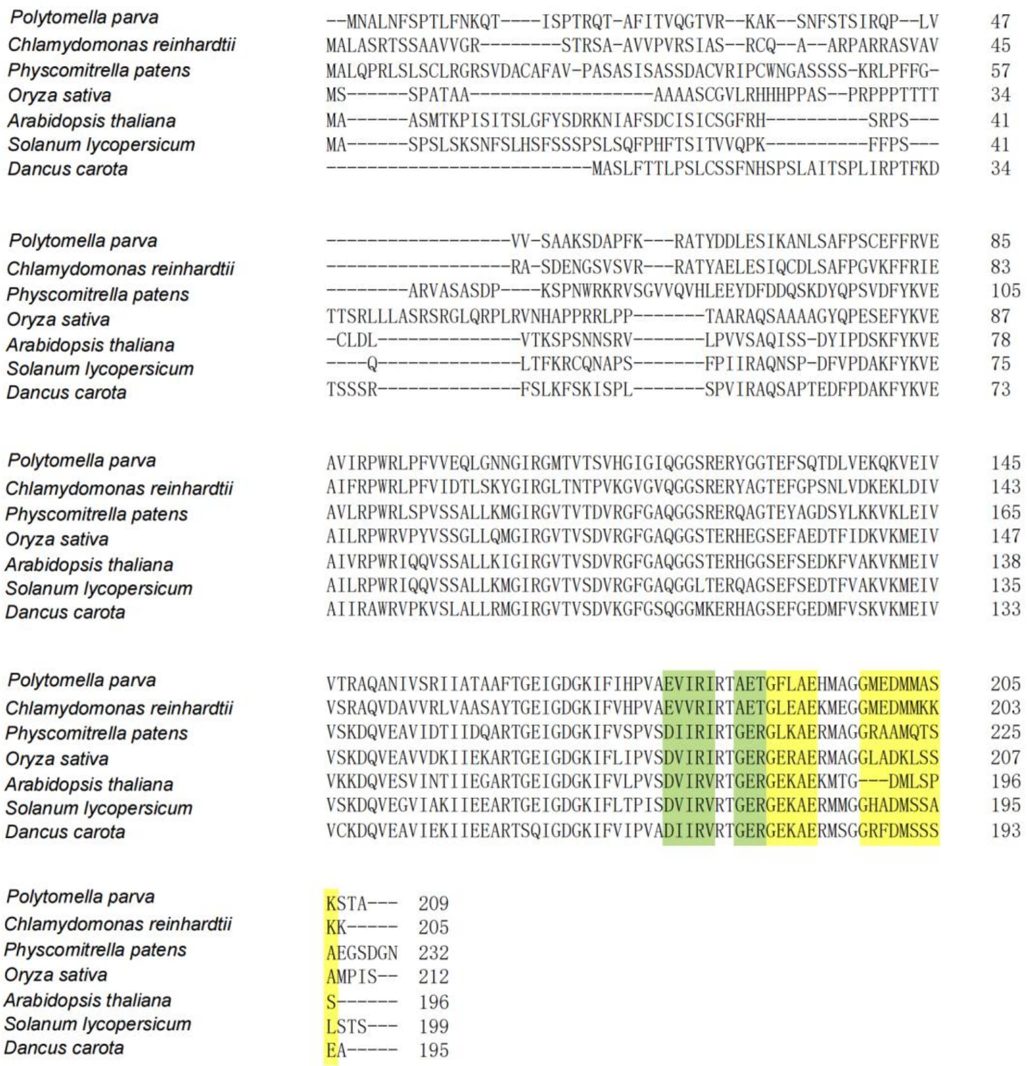

Supplementary Fig. 49. Sequence alignment of PII of plants and green algae, highlighting the Q loop. Color labeling shows secondary structure assignment (strands, green; helices, yellow) according to the AtPII-AtNAGK complex structures.

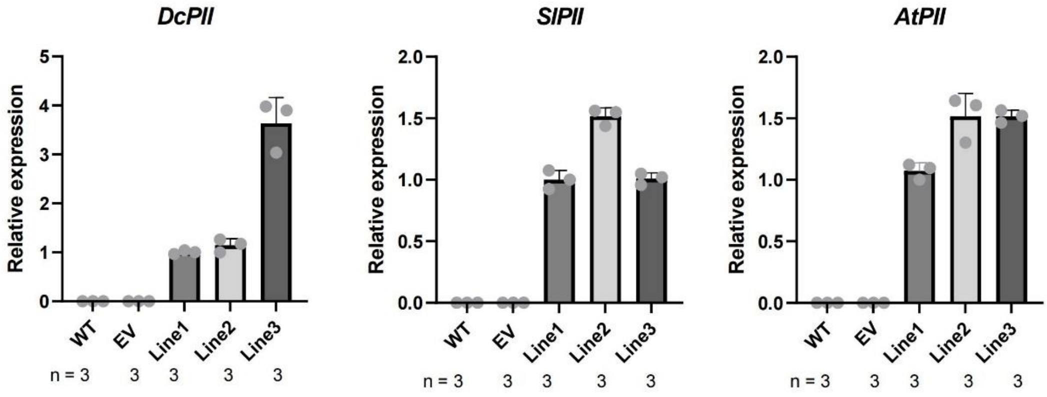

Supplementary Fig. 50. Real-Time qRT-PCR of the *PIIs* in wide-type *PII*-expressing lettuce plants.

WT, wild-type lettuce plants; EV, empty vector control; Line 1~3, L1, L2, and L3 represent three independent transgenic lines; The Data are presented as mean ± SD. The number (n) of independent arrays for each sample was shown below the x-axes. Source data are provided as a Source Data file.

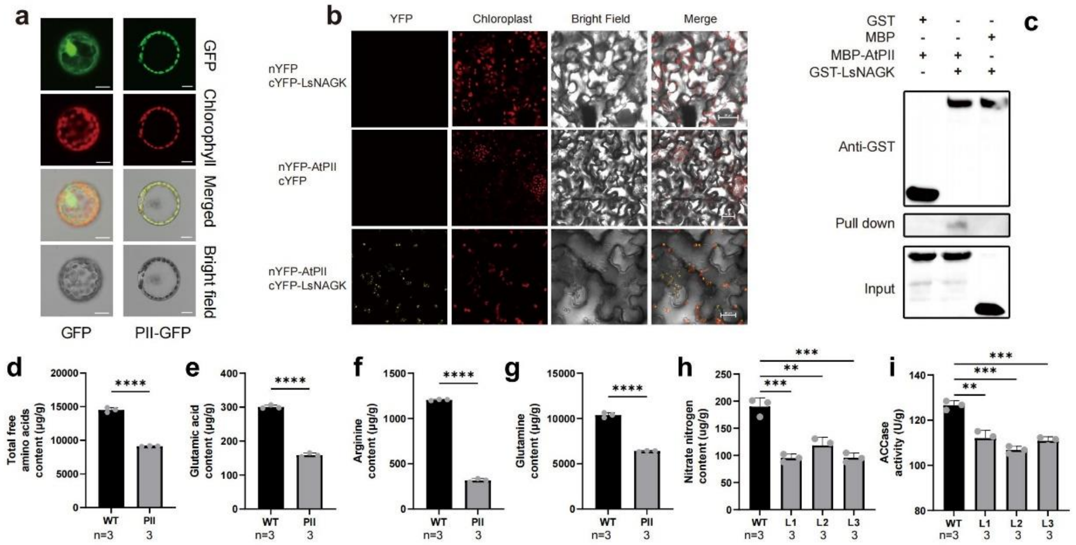

Supplementary Fig. 51. Functional study of expressing *AtPII* in lettuce.

a, Validation of chloroplast localization of PII in lettuce plants heterologously expressing Arabidopsis PII. We used confocal microscopy to visualize the localization of the PII-GFP (green fluorescent protein) fusion protein compared to that of free GFP in mesophyll protoplasts prepared from transgenic lettuce. Scale bars, 10 μm. **b**, Bimolecular fluorescence complementation (BiFC) analysis showing the interaction between LsNAGK and PIIs *in vivo*. **c**, GST pull-down assays showing the interaction between LsNAGK and PIIs in vitro. The proteins were detected by Western blot analysis with anti-MBP and anti-GST antibodies. **d-g**, Quantification of total free amino acids (d), glutamic acid (e), arginine (f), glutamine (g), in wide-type PII-expressing lettuce plants. WT: wide-type; PII: different lines of PII-expressing lettuce. **h-i**, Quantification of nitrate nitrogen (h), and ACCase enzyme activity (k) in wild-type and PII-expressing lettuce. L1, L2, and L3 represent three independent transgenic lines. Three independent assays (n=3) were conducted and data were presented as the mean ± SD. Statistical significance was determined using a two-sided Student's *t*-test. ***P*<0.01, ****P*<0.001, *****P*<0.0001. Three times each experiment was repeated independently with similar results. Source data are provided as a Source Data file.

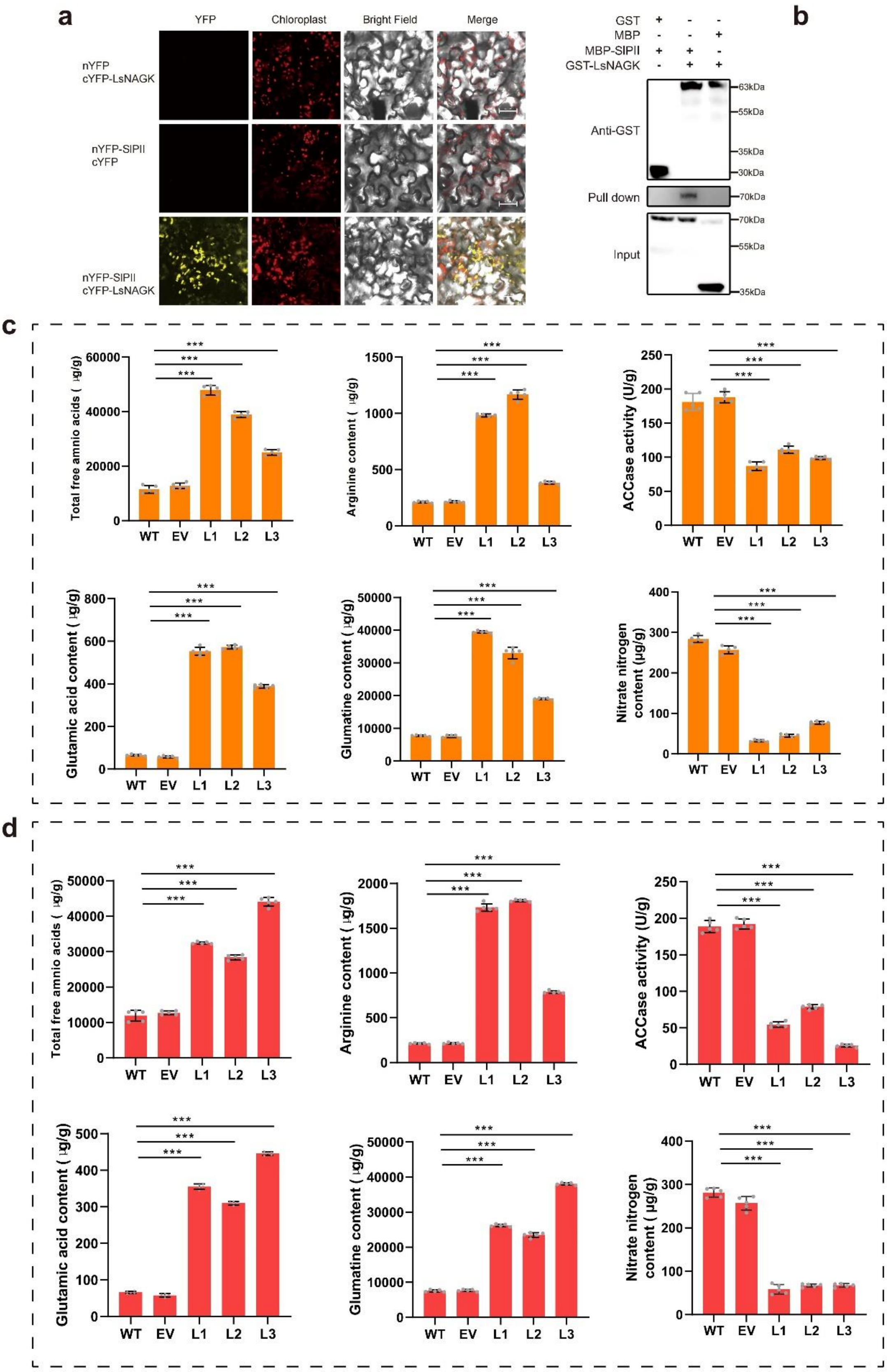

Supplementary Fig. 52. Functional study of expressing *DcPII and SlPII* in lettuce.

a, Bimolecular fluorescence complementation (BiFC) analysis showing the interaction between LsNAGK and PII (SIPII) *in vivo*. Three times each experiment was repeated independently with similar results. **b**, GST pull-down assays showing the interaction between LsNAGK and PII (SIPII) *in vitro*. The proteins were detected by Western blot analysis with anti-MBP and anti-GST antibodies. Three times each experiment was repeated independently with similar results. **c**, Quantification of total free amino acids, glutamic acid, arginine, glutamine, nitrogen, and ACCase enzyme activity in wide-type and DcPII-expressing lettuce plants. **d,** Quantification of total free amino acids, glutamic acid, arginine, glutamine, nitrogen, and ACCase enzyme activity in wild-type and SIPII-expressing lettuce plants. L1, L2, and L3 represent three independent transgenic lines. WT, wild-type; EV, empty vector control; Five independent assays (n=5) were conducted and data were presented as the mean ± SD. Statistical significance was determined using a two-sided Student's *t*-test. $^{**}P<0.01$, $^{***}P<0.001$, $^{****}P<0.0001$. Source data are provided as a Source Data file.

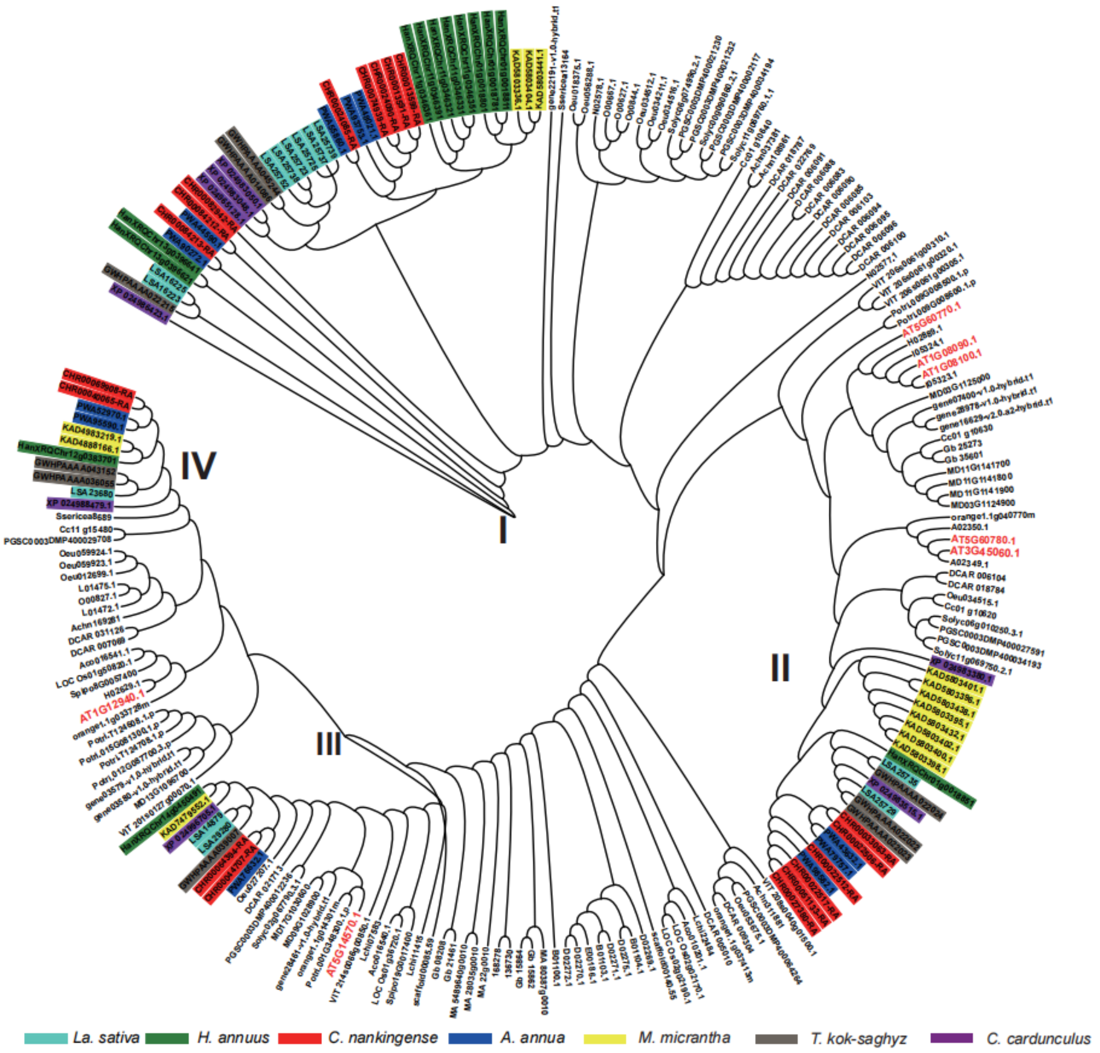

Supplementary Fig. 53. Phylogenetic tree of the NRT2 protein family in investigated species. Genes from seven Asteraceae species (*Lactuca sativa* var. *angustana*, *Cynara cardunculus* var. *scolymus*, *Helianthus annuus*, *Chrysanthemum nankingense*, *Artemisia annua*, *Taraxacum kok-saghyz* Rodin, and *Mikania micrantha*) are shown with different colors. Genes from *Arabidopsis thaliana* are shown in red text.

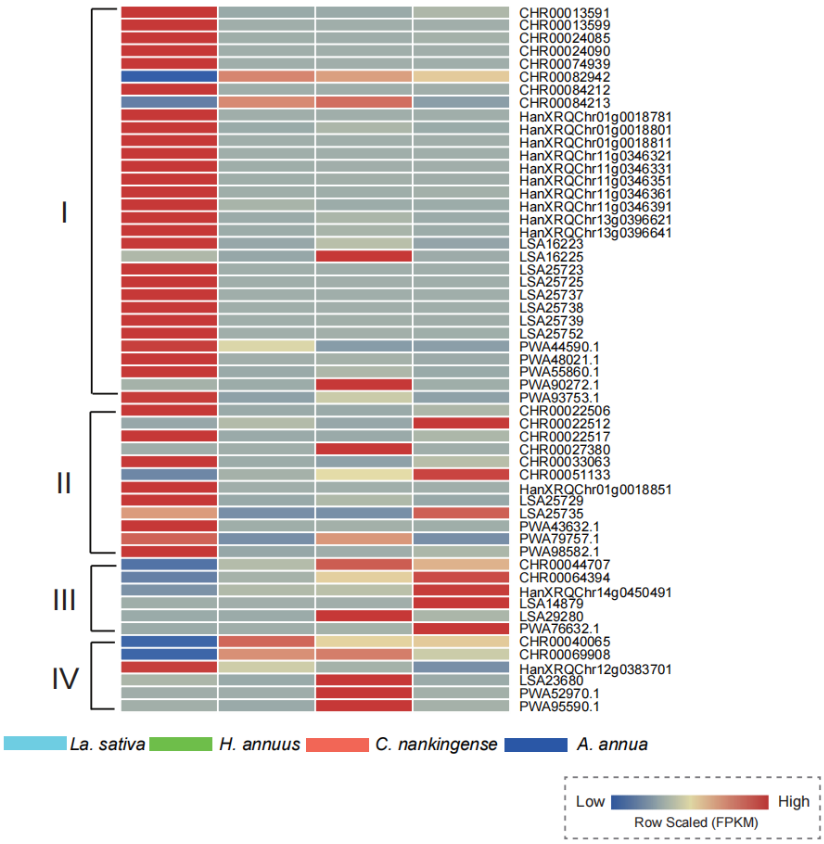

Supplementary Fig. 54. Heatmap representation of gene expression levels of *NRT2* members in different tissues from four Asteraceae species: *Lactuca sativa* var. *angustana*, *Cynara cardunculus* var. *scolymus*, *Helianthus annuus*, and *Chrysanthemum nankingense*.

Source data are provided as a Source Data file.

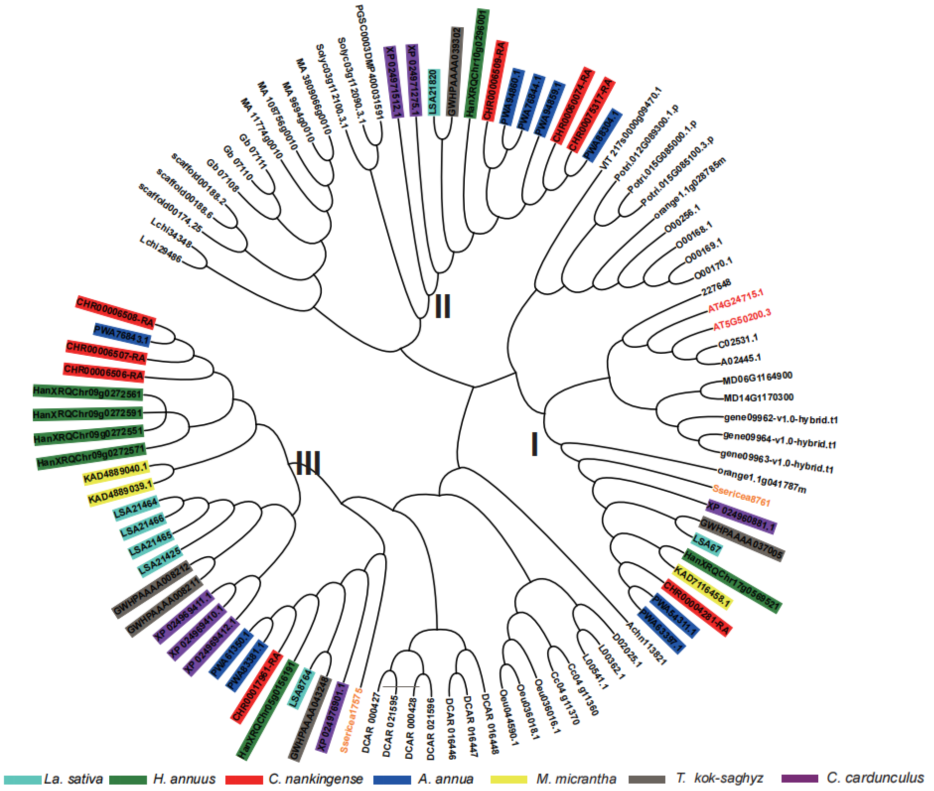

Supplementary Fig. 55. Phylogenetic tree of the NRT3 protein family in the investigated species.

Proteins from the Asteraceae family (*Lactuca sativa* var. *angustana*, *Cynara cardunculus* var. *scolymus*, *Helianthus annuus*, *Chrysanthemum nankingense*, *Artemisia annua*, *Taraxacum kok-saghyz* Rodin, and *Mikania micrantha*) are indicated in different colors. Genes from *Arabidopsis thaliana* are shown in red text.

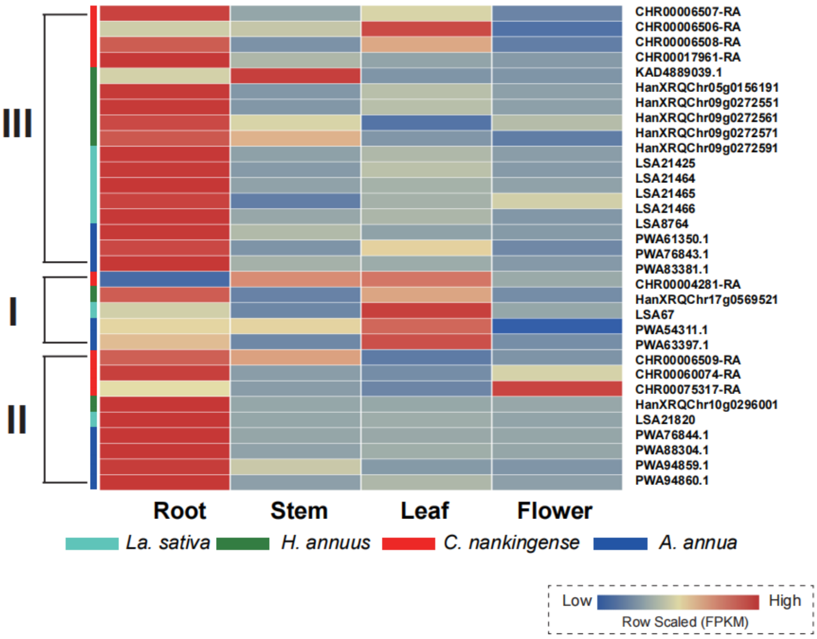

Supplementary Fig. 56. Heatmap representation of the gene expression of *NRT3* members in different tissues from the Asteraceae species.

Genes from the Asteraceae family (*Lactuca sativa* var. *angustana*, *Cynara cardunculus* var. *scolymus*, *Helianthus annuus*, *Chrysanthemum nankingense*, *Artemisia annua*, *Taraxacum kok-saghyz* Rodin, and *Mikania micrantha*) are indicated in different colors.

Source data are provided as a Source Data file.

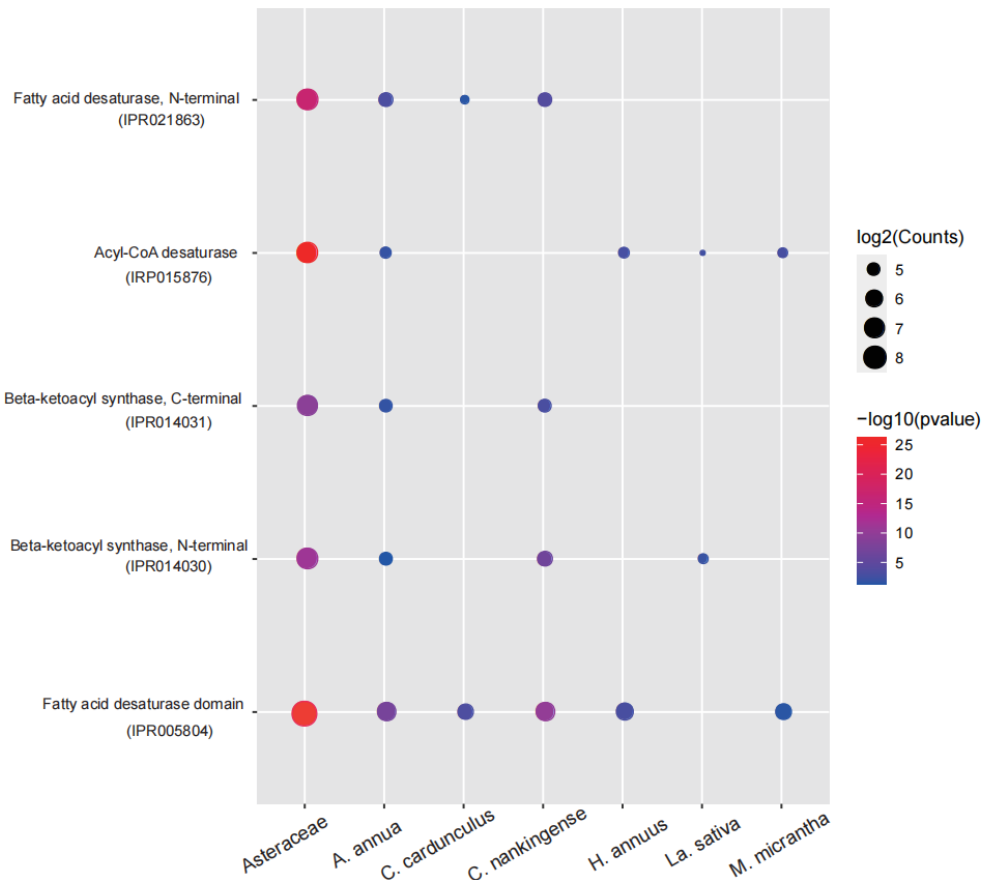

Supplementary Fig. 57. Enriched InterPro entries involved in fatty acid biosynthesis in the Asteraceae family.

A one-tailed Fisher's test was adopted to identified the enriched IntroPro entries. Source data are provided as a Source Data file.

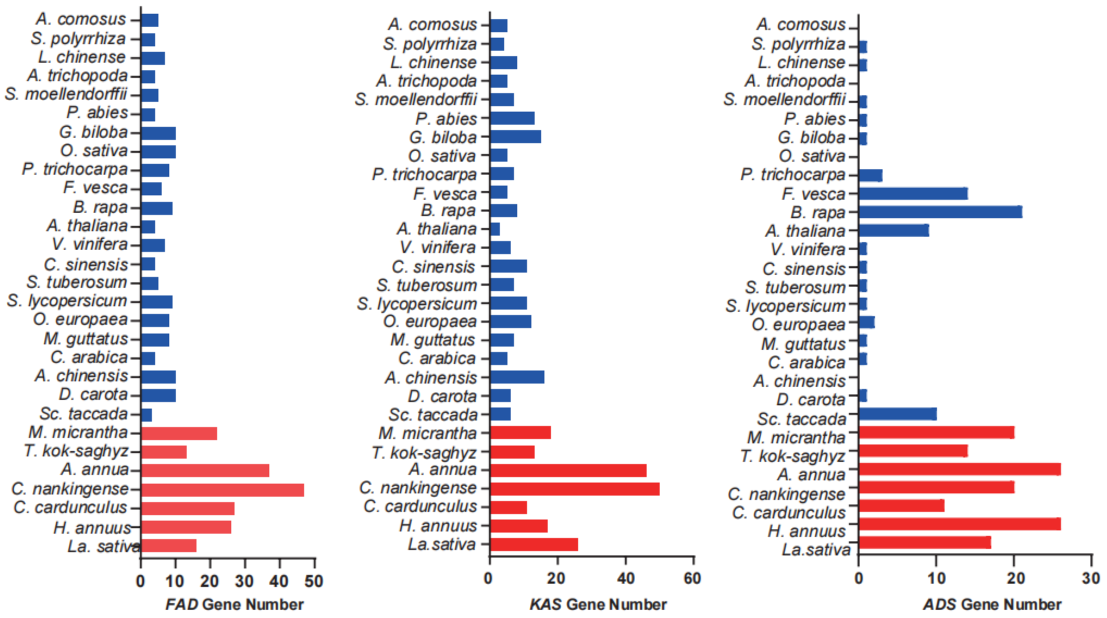

Supplementary Fig. 58. Numbers of *FAD*, *KAS*, and *ADS* genes in the investigated species. Source data are provided as a Source Data file.

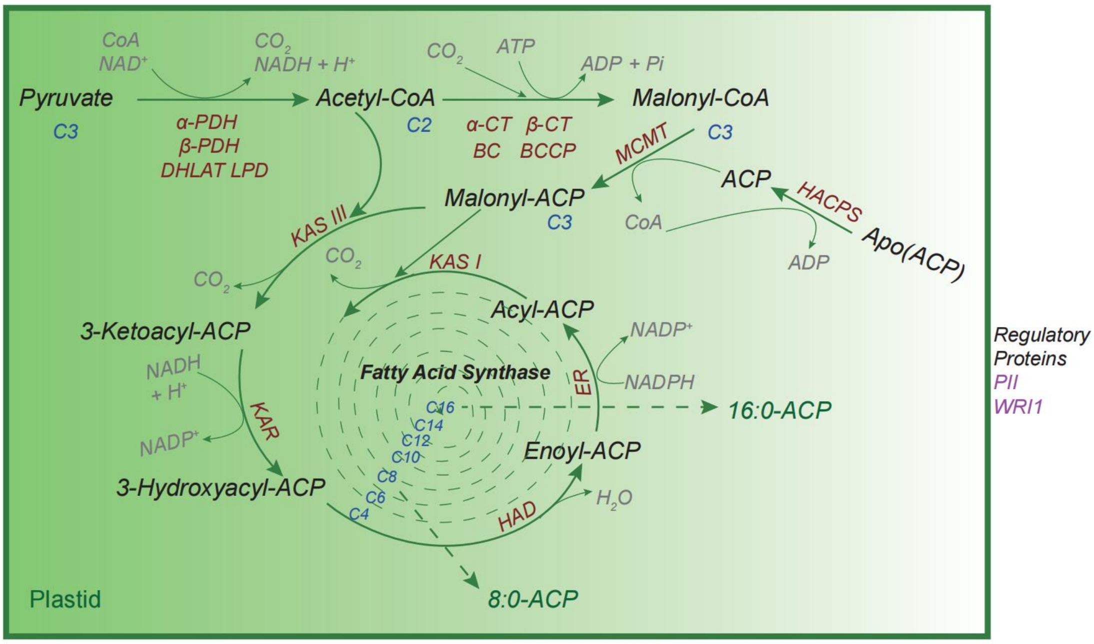

Supplementary Fig. 59. Schematic diagram of the fatty acid biosynthesis pathway.

The plastid pyruvate dehydrogenase complex generates acetyl-coenzyme A that is used as a building block for fatty acid production. Fatty acid chains extend by sequential condensation of two-carbon units catalyzed by enzymes of the fatty acid synthase complex. During each cycle, four reactions take place: condensation, reduction, dehydration, and reduction. Acyl carrier protein is a cofactor in all reactions. Biosynthesis of a C16 fatty acid requires that the cycle be repeated seven times. During the first round of the cycle, the condensation reaction is catalyzed by ketoacyl-ACP synthase (KAS) III. For the next six rounds of the cycle, the condensation reaction is catalyzed by isoform I of KAS. Finally, KAS II is used during the conversion of 16:0 to 18:0.

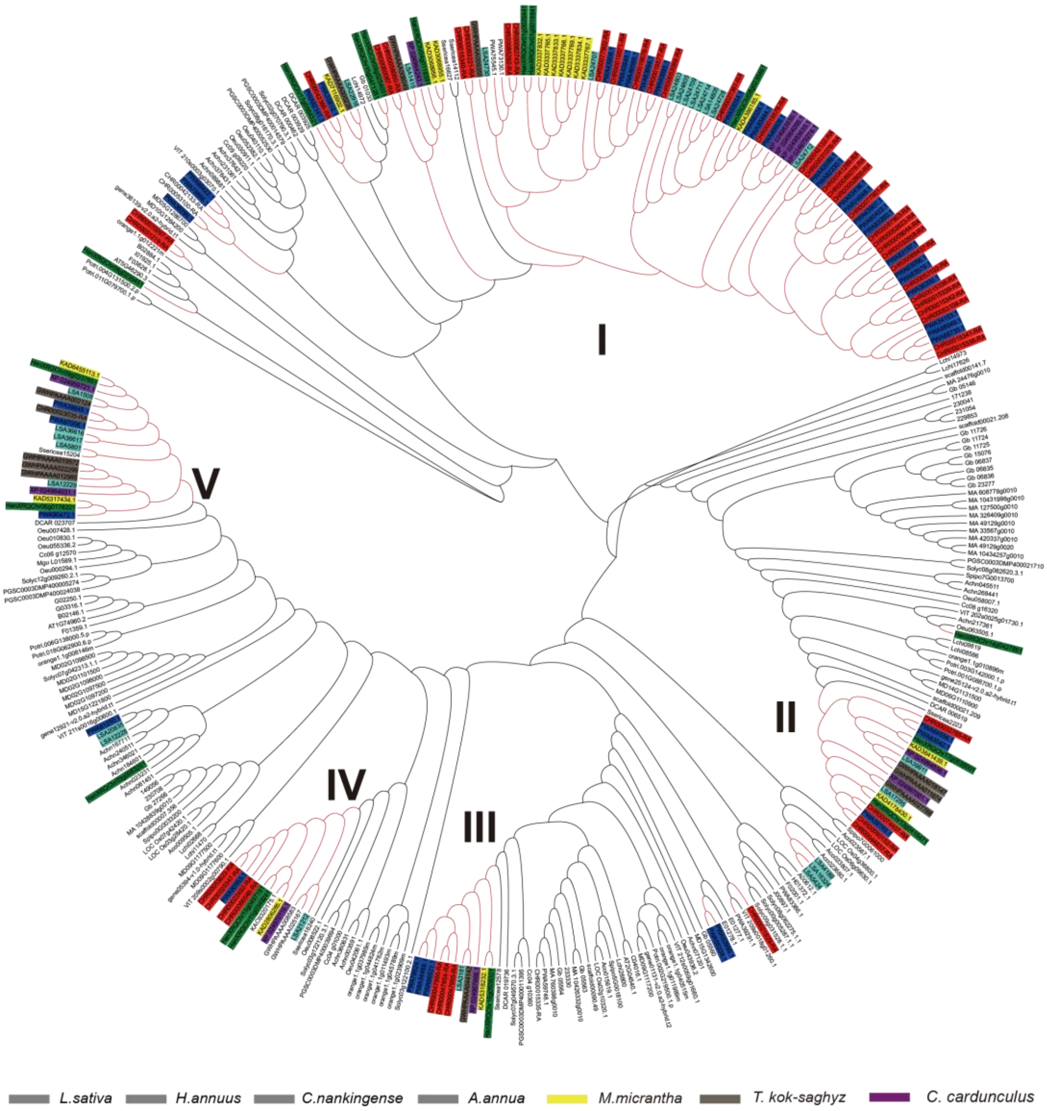

Supplementary Fig. 60. Phylogenetic tree of the KAS protein family in the investigated species. Proteins from seven Asteraceae species (*Lactuca sativa* var. *angustana*, *Cynara cardunculus* var. *scolymus*, *Helianthus annuus*, *Chrysanthemum nankingense*, *Artemisia annua*, *Taraxacum kok-saghyz* Rodin, and *Mikania micrantha*) are indicated in different colors.

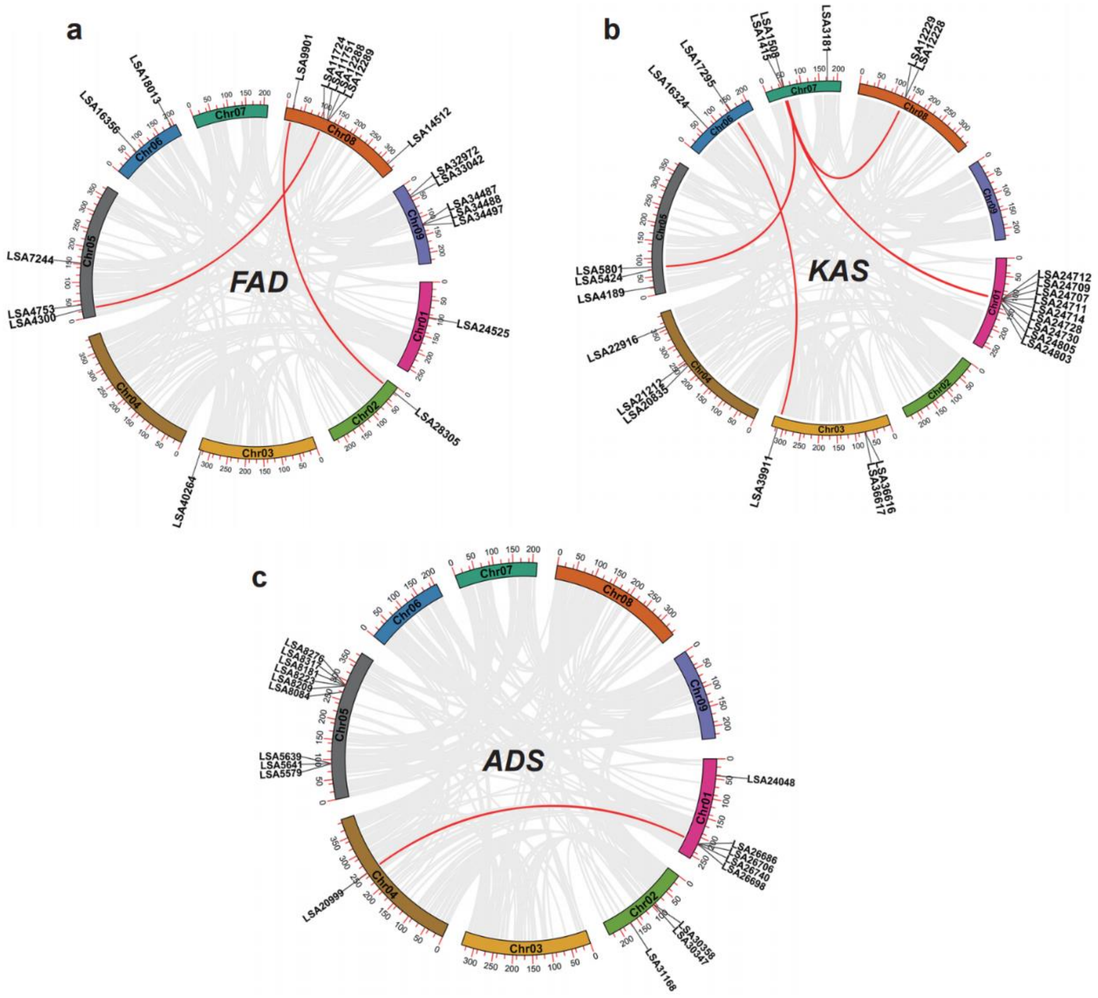

Supplementary Fig. 61. The genomic locations of *FAD*, *KAS*, and *ADS* gene families in the *Lactuca sativa* genome.

The karyotypes are represented as Circos plots. The orthologous gene pairs in the synteny blocks are linked with red lines. The gene names of (**a**) *FAD*s, (**b**) *KAS*s, and (**c**) *ADS*s are shown, and genes located in the syntenic blocks are highlighted with red lines.

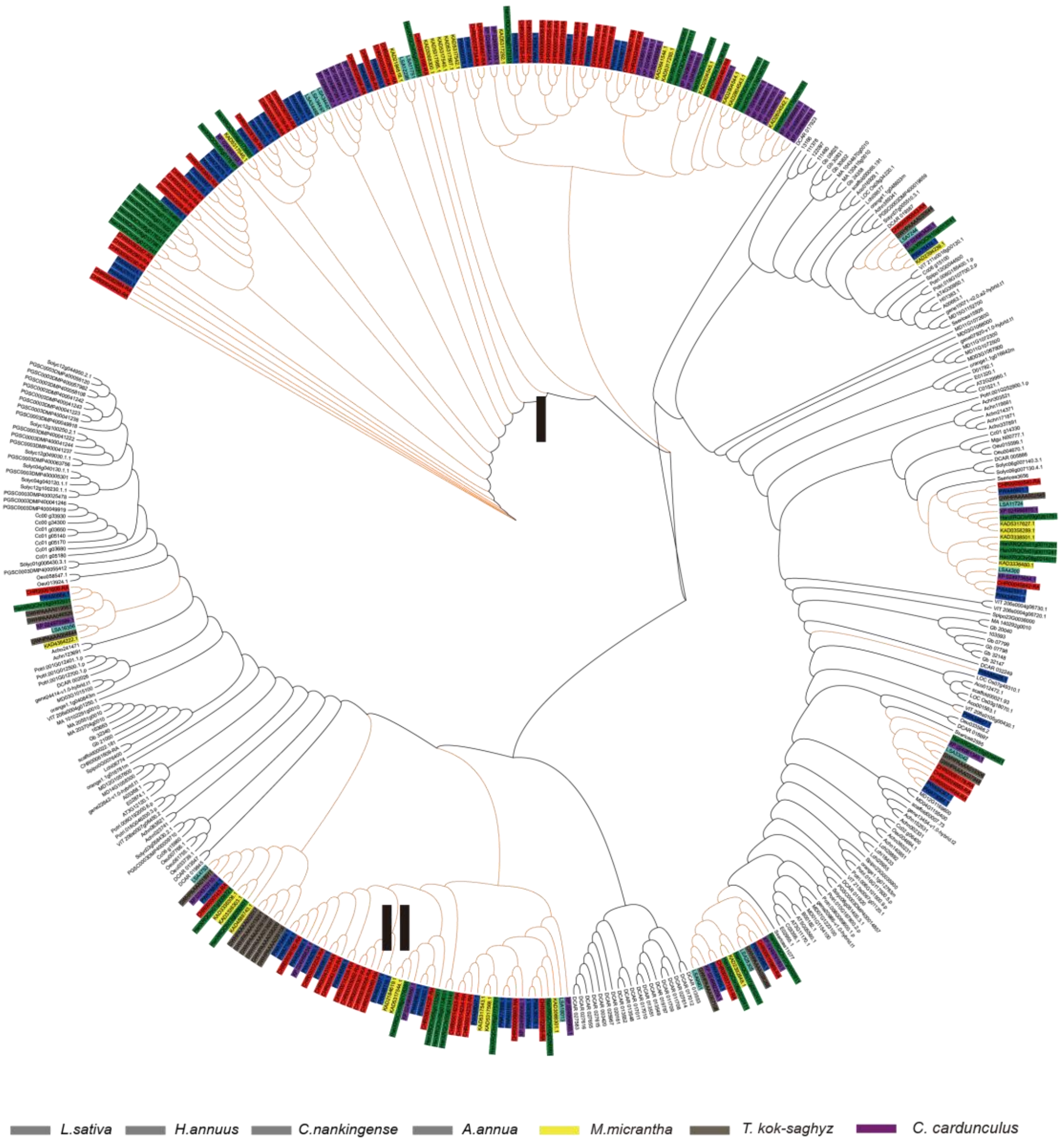

Supplementary Fig. 62. Phylogenetic tree of the FAD protein family in the investigated species. Proteins from seven Asteraceae species (*Lactuca sativa* var. *angustana*, *Cynara cardunculus* var. *scolymus*, *Helianthus annuus*, *Chrysanthemum nankingense*, *Artemisia annua*, *Taraxacum kok-saghyz* Rodin, and *Mikania micrantha*) are indicated in different colors.

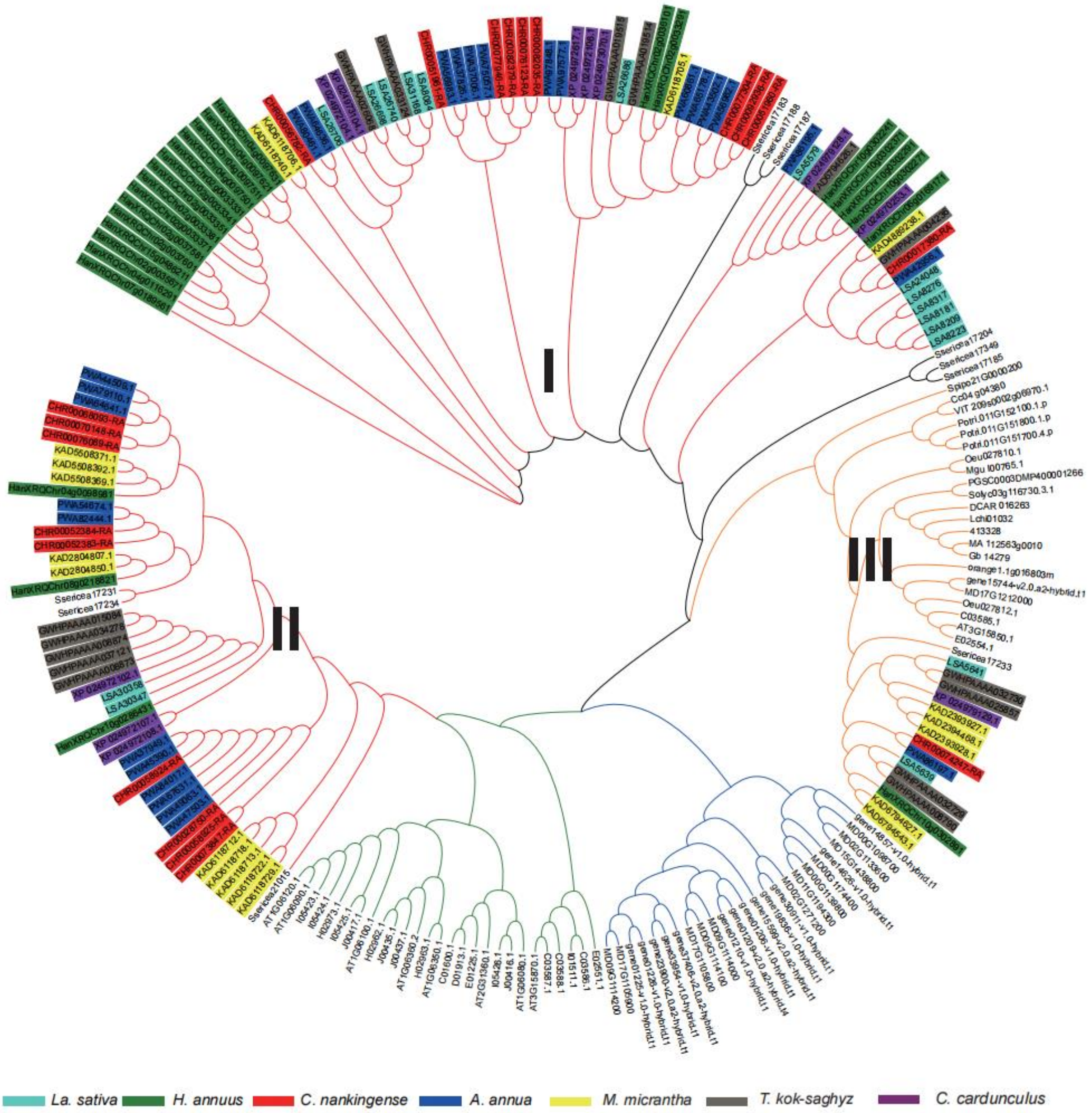

Supplementary Fig. 63. Phylogenetic tree of the ADS protein family in the investigated species. Proteins from seven Asteraceae species (*Lactuca sativa* var. *angustana*, *Cynara cardunculus* var. *scolymus*, *Helianthus annuus*, *Chrysanthemum nankingense*, *Artemisia annua*, *Taraxacum kok-saghyz* Rodin, and *Mikania micrantha*) are indicated in different colors.

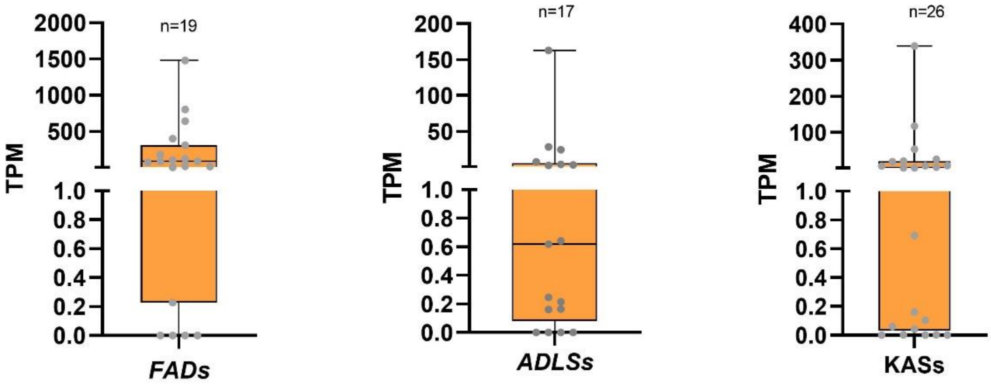

Supplementary Fig. 64. The expression levels (TPM) of the *FAD*s, *ADLS*s, *KAS*s genes in lettuce.

The genes were quantified by RNA-seq from different tissues, including root, stem, leaf, and flower. The expression levels were summed to obtain the final data. TPM: Transcripts Per Million. In box plots, central line: median values; box boundaries: 25th and 75th percentiles; whiskers: 1.5 * IQR (IQR: the interquartile range between the 25th and 75th percentile). The number (n) of data point for each gene was indicated in the figure.

Source data are provided as a Source Data file.

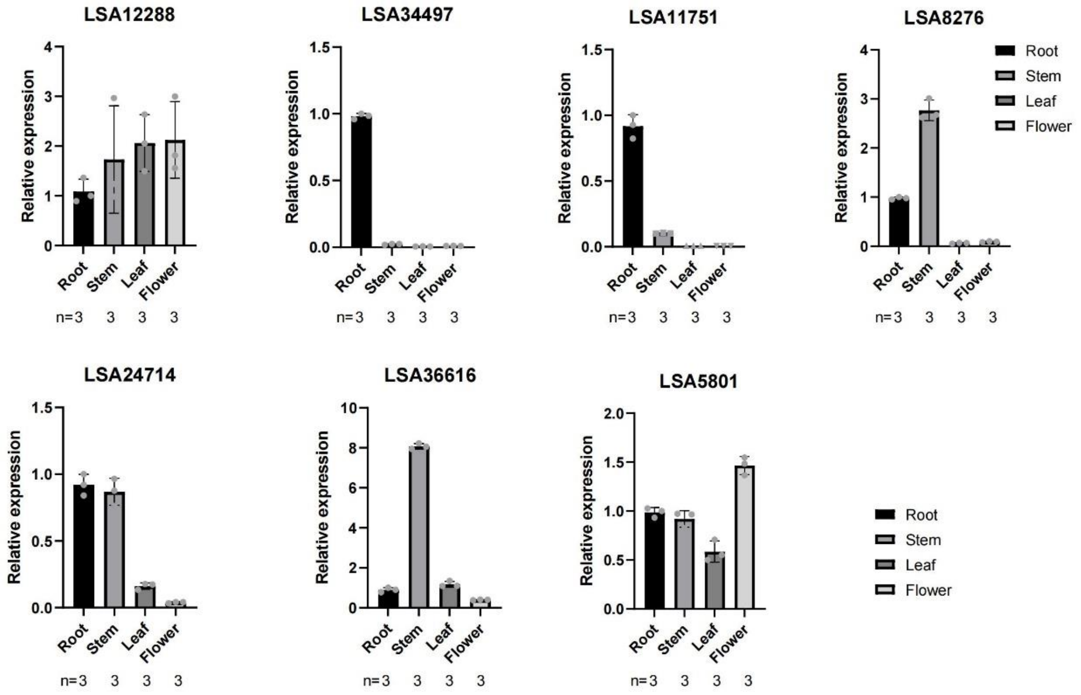

Supplementary Fig. 65. Real-Time qRT-PCR of the duplicated FAD genes in lettuce.

The number (n) of independent assays shown below the x-axes. Data were presented as the mean ± SD. Source data are provided as a Source Data file.

Supplementary Table 1. Raw libraries of *Lactuca sativa* var. *augustana*.

Source	Platform/ Tissue	Library type	Data					
			No. reads	Average length (nt)	N50 length	Label density (/100kb)	Total bases (Gb)	Depth (x)
Genome	Illumina	Paired-end	2,845,110,230	150	150	---	426.8	158
	HiC	Paired-end	1,952,521,748	150	150	---	292.9	108
	PacBio	CLR	20,020,790	14,231	19,814	---	284.7	105
	Bionano	Saphyr	1,318,023	242,963	279,750	17.419	320.2	119
RNA-Seq	Flower	Paired-end	62,893,268	150	150	---	9.4	---
	Leaf	Paired-end	27,622,152	150	150	---	4.1	---
	Stem	Paired-end	47,387,729	150	150	---	7.1	---
	Root	Paired-end	33,357,143	150	150	---	5.0	---

Supplementary Table 2. Statistics of the assembly process of the stem lettuce genome.

Assembly stage	Stage 1[a]	Stage 2[b]	Stage 3[c]
Contig/Scaffold number	1,959	27	9
Total length (bp)	2,597,237,272	2,589,460,708	2,589,759,771
Longest contig/Scaffold (bp)	20,983,318	332,303,623	397,864,723
N10 (bp)	12,801,060	332,303,623	397,864,723
N20 (bp)	8,826,272	266,214,141	377,806,080
N30 (bp)	7,409,652	229,960,483	346,092,795
N40 (bp)	6,057,126	205,685,218	346,092,795
N50 (bp)	4,950,584	186,521,119	332,303,623
N60 (bp)	4,067,331	153,625,972	258,638,545
N70 (bp)	3,165,126	130,827,339	230,623,264
N80 (bp)	2,424,089	114,737,243	229,960,483
N90 (bp)	1,417,346	86,202,022	210,785,040

Note: Stage 1a: the assembly generated only by PacBio data.

Stage 2b: the assembly generated by PacBio data and Bionano data.

Stage 3c: the assembly generated by PacBio, Bionano and HiC data.

Supplementary Table 3. Lettuce genome sequence assembly organized by pseudomolecules (chromosomes).

Pseudomolecule	Length (nt)	No. of sequences*	No. of superscaffolds**	Average superscaffold length (nt)	Total superscaffold length (nt)
Chr00	30,866,348	640	3	2,885,271	8,655,813
Chr01	258,638,545	17	4	64,560,433	258,241,731
Chr02	230,623,264	10	3	76,782,220	230,346,660
Chr03	332,303,623	1	1	332,303,623	332,303,623
Chr04	397,864,723	17	3	132,443,911	397,331,732
Chr05	377,806,080	37	6	62,779,626	376,677,756
Chr06	205,685,218	1	1	205,685,218	205,685,218
Chr07	210,785,040	11	2	105,198,609	210,397,217
Chr08	346,092,795	156	3	113,286,825	339,860,475
Chr09	229,960,483	1	1	229,960,483	229,960,483
Total	2,620,626,119	891	24	107,894,196	2,589,460,708

*: Including contigs and scaffolds. **: Superscaffolds generated by Bionano data

Supplementary Table 4. Statistics of protein-coding genes in the stem lettuce genome.

Type		Number	Percentage (%)
**Annotation**	Swissprot	26895	66.67%
	Interpro	34150	84.65%
	KEGG	14888	36.91%
	GO	21097	52.30%
**Total**	Annotated	34966	86.68%
	Gene	40341	

Supplementary Table 5. Statistics of identified non-coding RNAs in the stem lettuce genome.

Pipeline	Type	Number
RepeatMasker	rRNA	2080
RepeatMasker	tRNA	1144
RepeatMasker	snRNA	590
Maker-P (trna-scan)	tRNA	1111
Rfam	SRP RNA	90
Rfam	microRNA	137
Rfam	snoRNA	278
Rfam	snRNA	23
Total		5453

Supplementary Table 6. Statistics of Illumina pair-end reads mapped to the lettuce assembly.

PE library	Cleaned read pairs	Properly paired mapped	Percent
In this study	1,964,393,808	1,932,299,337	98.37%
Salinas	1,768,300,285	1,672,209,990	94.57%

Supplementary Table 7. Assessment of the gene space coverage using publicly available lettuce EST data.

Dataset	Number of ESTs	Number of mapped ESTs	Mapped ratio (%)	Total length (nt)	Mapped length
All	81,330	77,520	95.32	53,433,457	46,079,376
≥200nt	78,837	75,589	95.88	53,039,882	45,787,962
≥500nt	60,633	59,077	97.43	46,712,585	40,491,667

Supplementary Table 8. Assessment of the gene space coverage based upon alignment of the RNA-Seq data obtained from different tissues against the lettuce assembly.

Resource type	Tissue type	Number of reads pairs	Percent aligned
Illumina, PE	Root	33,357,143	97.18%
Illumina, PE	Leaf	27,622,152	97.46%
Illumina, PE	Flower	62,893,268	96.59%
Illumina, PE	Stem	47,387,729	97.97%
Total RNA seq		171,260,292	97.30%

Supplementary Table 9. BUSCO statistics of the lettuce genome.

Database odb10	Genome		High-confidence gene set	
	Number	%	Number	%
Complete BUSCOs (C)	1312	95.42%	1268	92.22%
Complete and single-copy BUSCOs (S)	1256	91.35%	1211	88.07%
Complete and duplicated BUSCOs (D)	56	4.07%	57	4.15%
Fragmented BUSCOs (F)	8	0.58%	51	3.71%
Missing BUSCOs (M)	55	4.00%	56	4.07%
Total BUSCO groups searched	1375	100.00%	1375	100.00%

Supplementary Table 10. Organization of repetitive sequences in the lettuce genome.

	Number*	Length (bp)	Percent of assembled genome
Total repeat fraction	3,878,044	2,304,889,068	87.85%
Mobile Element		2,275,929,496	86.85 %
Class I: Retroelement	2,483,282	2,086,817,471	79.63%
LTR Retrotransposon	2,466,671	2,078,079,576	79.30 %
non-LTR Retrotransposon	16,611	8,737,895	0.33%
LINE	14,904	8,373,697	0.32 %
SINE	1,707	364,198	0.01 %
Class II: DNA Transposon	88,596	37,873,059	1.45 %
Unclassified	812,162	151,238,966	5.77 %
Tandem Repeats	494,004	28,959,572	1.00%
Satellites	2,723	592,198	0.02 %
Simple repeats	455,672	26,553,909	1.01 %
Low complexity	35,609	1,813,465	0.07 %

Supplementary Table 11. Potential telomeric sites identified by searching both ends of the pseudo-chromosomes for high copy number repeats with the repeat unit 5-TTTAGGG-3 in the stem lettuce genome.

Chromosome	Begin position	End position	Chromosome length (nt)	Distance (nt)	Motif
Chr01	258,617,095	258,617,403	258,638,545	21,142	Target "Motif:(TTTAGGG)n" 1 315
Chr01	258,619,293	258,619,601	258,638,545	18,944	Target "Motif:(TTTAGGG)n" 1 315
Chr02	230,581,337	230,581,592	230,623,264	41,672	Target "Motif:(TTTAGGG)n" 1 255
Chr03	332,261,167	332,261,299	332,303,623	42,324	Target "Motif:(TTTAGGG)n" 1 136
Chr05	377,803,014	377,806,080	377,806,080	0	Target "Motif:(TTTAGGG)n" 1 3084
Chr07	210,783,892	210,784,780	210,785,040	260	Target "Motif:(TTTAGGG)n" 1 885

Supplementary Table 12. Raw libraries of *Scaevola taccada*.

Source	Platform	Library type	Data						
			No. reads pairs	Total bases	Depth (x)	Average length (nt)	Max length (nt)	Min length (nt)	N50 length (nt)
	Illumina	Paired-end	770,834,028	115,625,104,200	105	---	---	---	---
	HiC	Paired-end	746,645,416	111,996,812,400	102	---	---	---	---
	PacBio	CLR	6695768	111,358,850,139	102	16631.23	246999	100	25395
RNA-Seq	Leaf	Paired-end	22,631,051	6,789,315,300	---	---	---	---	---
	Stem	Paired-end	23,076,016	6,922,804,800	---	---	---	---	---
	Flower	Paired-end	22,950,831	6,885,249,300	---	---	---	---	---
	Root	Paired-end	19,380,453	5,814,135,900	---	---	---	---	---

Supplementary Table 13. Statistics of genome assembly of *Scaevola taccada*.

**Chromosome**	**Length (nt)**
Chr01	173,820,968
Chr02	162,192,376
Chr03	158,120,924
Chr04	138,867,822
Chr05	136,048,263
Chr06	131,537,989
Chr07	129,547,837
Chr08	106,008,981
Chr00	22,714,821
Total	1,158,859,981

Supplementary Table 14. Statistics of initial genome assembly of *Scaevola taccada*.

Category	Value
Total length (nt)	1,158,970,963
Longest contig (bp)	58,091,406
N10 (bp)	29,738,695
N50 (bp)	9,636,078
N90 (bp)	1,010,778
Contig #	499

Supplementary Table 15. Organization of repetitive sequences in the *Scaevola taccada* genome.

	Number*	Length (bp)	Percent of assembled genome
Total repeat fraction		952,316,438	80.69 %
Mobile Element		948,096,315	80.33 %
Class I: Retroelement			
LTR Retrotransposon	771,536	782,658,701	66.32 %
non-LTR Retrotransposon			
LINE	13,323	4,159,639	0.35 %
SINE	2,568	379,310	0.03 %
Class II: DNA Transposon	95,534	41,389,895	3.51 %
Unclassified	540,055	119,508,770	10.13 %
Tandem Repeats			
Satellites	1,677	571,042	0.05 %
Simple repeats	210,558	10,822,439	0.92 %
Low complexity	37,096	1,807,773	0.15 %
Small RNA	5,079	708486.0	0.06 %

Supplementary Table 16. Statistics of protein-coding genes in the the *Scaevola taccada* genome.

Type		Number	Percentage (%)
Annotation	Swissprot	16817	66.40%
	Interpro	22777	89.93%
	KEGG	8390	33.13%
	GO	20145	79.54%
Total	Annotated	25003	98.72%
	Gene	25328	

Supplementary Table 17. Statistics of non-coding RNAs in the *Scaevola taccada* genome.

Type	Number
rRNA	1654
tRNA	829
SRP RNA	40
microRNA	1516
snoRNA	17
snRNA	163
Total	4219

Supplementary Table 18. BUSCO statistics of the genome assembly of *Scaevola taccada*.

Database odb10	Genome		High-confidence gene set	
	**Number**	**%**	**Number**	**%**
Complete BUSCOs (C)	1296	94.20%	1258	91.50%
Complete and single-copy BUSCOs (S)	1279	93.00%	1228	89.30%
Complete and duplicated BUSCOs (D)	17	1.20%	30	2.20%
Fragmented BUSCOs (F)	14	1.00%	69	5.00%
Missing BUSCOs (M)	65	4.80%	48	3.50%
Total BUSCO groups searched	1375	100.00%	1375	100.00%

Supplementary Table 19. Statistics of Illumina PE reads mapped to the genome assembly of *Scaevola taccada*.

Resource type	Tissue type	Number of reads pairs	Percent aligned
Illumina, PE, DNA	Leaf	770,834,028	98.90%
Illumina, PE, RNA	Leaf	22,631,051	89.64%
Illumina, PE, RNA	Stem	23,076,016	90.32%
Illumina, PE, RNA	Flower	22,950,831	93.58%
Illumina, PE, RNA	Root	19,380,453	93.92%
Total RNA seq		88,038,351	

Supplementary Table 20. Statistics of the genes and synteny blocks in the triplication retained genomic regions of the Asteraceae family.

Species[a]	Poidy[b]	Synteny blocks[c]	Triplicated retention regions[d]	Retention genes[e]
Lactuca sativa	3n	524	468	1406
Cynara cardunculus	3n	656	597	1473
Helianthus annuus	6n	1534	506	946
Mikania micrantha	6n	1170	545	1394

Note: **a**, chromosome-scale genomes in the Asteraceae family; **b**, the relative ploidy when Scaevola(n) and Vitis (n); **c**, the identified synteny blocks versus Scaevola; **d**, the retained genomic regions after WGT-1 event in the Asteraceae family; **e**, the retention genes in the triplication retained regions.

Supplementary Table 21. Statistics of transcription factor binding site (TFBS) introduced by repeat sequences in the Asteraceae family.

Species	Affected genes	Possibly introduced TFBS	Whole gene set
Lactuca sativa	1254	4913	40341
Cynara cardunculus	884	2400	26326
Helianthus annuus	1163	4013	52243
Mikania micrantha	1806	7176	46329

Supplementary Table 22. Statistics of the candidate genes of the FERONIA family in the surveyed species.

Species	# blastp (best hit)	# genes containing malectin-like	# genes not containing malectin-like
Lactuca sativa var. angustana	169	14	155
Cynara cardunculus var. scolymus	53	17	36
Artemisa annua	288	26	262
Chrysanthemum nankingense	483	24	459
Helianthus annuus	284	44	240
Taraxacum kok-saghyz Rodin	160	18	142
Mikania micrantha	78	16	62
Scaevola sericea	22	11	11
Oryza sativa	27	18	9
Solanum tuberosum	44	22	22
Liriodendron chinense	32	25	7
Fragaria vesca	25	22	3
Ginkgo biloba	14	6	8
Ananas comosus	15	10	5
Daucus carota	28	22	6
Arabidopsis thaliana	21	17	4
Mimulus guttatus	39	24	15
Citrus sinensis	25	16	9
Amborella trichopoda	14	9	5
Picea abies	21	10	11
Actinidia chinensis	60	41	19
Selaginella moellendorffii	8	2	6
Olea europaea	33	22	11
Brassica rapa	30	27	3
Coffea arabica	33	26	7
Vitis vinifera	47	29	18
Solanum lycopersicum	36	17	19
Populus trichocarpa	39	30	9
Spirodela polyrrhiza	14	11	3

Supplementary Table 23. The lineage-specific genes of Asteraceae family related to cell wall.

OG	Family	Description	Function
OG0016185	COBRA-like protein 4	Lipoprotein	Cell wall
OG0012721	Wall-associated receptor kinase 2	Receptor-like protein kinase	Cell wall
OG0015289	Beta-fructofuranosidase	Glycosyl hydrolase 32	Cell wall beta-fructosidase
OG0012717	Filament-like plant protein 7	Coiled-coil protein	Cell wall
OG0012698	Pectate lyase	Lyase	Cell wall
OG0014060	Pectinesterase 2	Hydrolase	Cell wall
OG0014037	Protein trichome birefringence-like 34	O-acetyltransferase	Cell wall

Supplementary Table 24. Statistics of lineage-specific lost orthogroups in the species of Asteraceae family.

Orthogroup ID	Pfam ID	Description
OG0007067	PF08127*	Peptidase family C1 propeptide
OG0007427	PF01925	Sulfite exporter TauE/SafE
OG0008158	PF09459*	Ethylbenzene dehydrogenase
OG0009054	PF05724	Thiopurine S-methyltransferase (TPMT)
OG0009375	PF00543*	Nitrogen regulatory protein P-II
OG0010854	PF00481	Protein phosphatase 2C

Note: * indicated that the Pfam domains were only belonged to the orthologous groups in the genome of each species.

Supplementary Table 25. Statistics of Asteraceae species in OneKP project.

Species	Family	Clade	Order	PII (Yes/NO)*
Inula helenium	Asteraceae	Asterids	Asterales	NO
Flaveria pringlei	Asteraceae	Asterids	Asterales	NO
Phelline lucida	Phellinaceae	Asterids	Asterales	YES
Flaveria cronquistii	Asteraceae	Asterids	Asterales	NO
Senecio rowleyanus	Asteraceae	Asterids	Asterales	NO
Carthamus lanatus	Asteraceae	Asterids	Asterales	NO
Tragopogon dubius	Asteraceae	Asterids	Asterales	NO
Erigeron canadensis	Asteraceae	Asterids	Asterales	NO
Leontopodium alpinum	Asteraceae	Asterids	Asterales	NO
Helenium autumnale	Asteraceae	Asterids	Asterales	NO
Tanacetum parthenium	Asteraceae	Asterids	Asterales	NO
Silybum marianum	Asteraceae	Asterids	Asterales	NO
Tragopogon pratensis	Asteraceae	Asterids	Asterales	NO
Stylidium adnatum	Stylidiaceae	Asterids	Asterales	YES
Corokia cotoneaster	Argophyllaceae	Asterids	Asterales	YES
Anthemis tinctoria	Asteraceae	Asterids	Asterales	NO
Scaevola mossambicensis	Goodeniaceae	Asterids	Asterales	NO
Flaveria vaginata	Asteraceae	Asterids	Asterales	NO
Platycodon grandiflorus	Campanulaceae	Asterids	Asterales	YES
Menyanthes trifoliata	Menyanthaceae	Asterids	Asterales	YES
Lobelia siphilitica	Campanulaceae	Asterids	Asterales	YES
Flaveria bidentis	Asteraceae	Asterids	Asterales	NO
Cicerbita plumieri	Asteraceae	Asterids	Asterales	NO
Flaveria brownii	Asteraceae	Asterids	Asterales	NO
Tragopogon castellanus	Asteraceae	Asterids	Asterales	NO
Tragopogon porrifolius	Asteraceae	Asterids	Asterales	NO
Flaveria pubescens	Asteraceae	Asterids	Asterales	NO
Conyza canadensis	Asteraceae	Asterids	Asterales	NO
Flaveria palmeri	Asteraceae	Asterids	Asterales	NO
Matricaria matricarioides	Asteraceae	Asterids	Asterales	NO
Flaveria kochiana	Asteraceae	Asterids	Asterales	NO
Lactuca graminifolia	Asteraceae	Asterids	Asterales	NO
Solidago canadensis	Asteraceae	Asterids	Asterales	NO
Platyspermation crassifolium	Alseuosmiaceae	Asterids	Asterales	YES
Xanthium strumarium	Asteraceae	Asterids	Asterales	NO
Flaveria sonorensis	Asteraceae	Asterids	Asterales	NO
Aster tataricus	Asteraceae	Asterids	Asterales	NO
Flaveria angustifolia	Asteraceae	Asterids	Asterales	NO
Flaveria trinervia	Asteraceae	Asterids	Asterales	NO

Note: * harboring PII or not

Supplementary references

1. Amborella Genome Project *et al.* The Amborella genome and the evolution of flowering plants. *Science.* **342**, 1241089 (2013).
2. Mandel, J. R. *et al.* A fully resolved backbone phylogeny reveals numerous dispersals and explosive diversifications throughout the history of Asteraceae. *Proc. Natl. Acad. Sci. U. S. A.* **116**, 14083–14088 (2019).
3. Panero, J. L. & Crozier, B. S. Macroevolutionary dynamics in the early diversification of Asteraceae. *Mol. Phylogenet. Evol.* **99**, 116–132 (2016).
4. Funk, V. a. *et al.* Everywhere but Antarctica: Using a supertree to understand the diversity and distribution of the Compositae. *K. Danske Vidensk. Selsk. Biol. Skr.* **55**, 343–373 (2005).
5. Barreda, V. D. *et al.* Early evolution of the angiosperm clade Asteraceae in the Cretaceous of Antarctica. *Proc. Natl. Acad. Sci. U. S. A.* **112**, 10989–10994 (2015).
6. Valluru, R. & Van Den Ende, W. Plant fructans in stress environments: Emerging concepts and future prospects. *J. Exp. Bot.* **59**, 2905–2916 (2008).
7. Wei, H. *et al.* CiMYB17, a stress-induced chicory R2R3-MYB transcription factor, activates promoters of genes involved in fructan synthesis and degradation. *New Phytol.* **215**, 281–298 (2017).
8. Kim, K. J., Choi, K. S. & Jansen, R. K. Two chloroplast DNA inversions originated simultaneously during the early evolution of the sunflower family (Asteraceae). *Mol. Biol. Evol.* **22**, 1783–1792 (2005).
9. Barker, M. S. *et al.* Multiple paleopolyploidizations during the evolution of the compositae reveal parallel patterns of duplicate gene retention after millions of years. *Mol. Biol. Evol.* **25**, 2445–2455 (2008).
10. Zhang, C. *et al.* Phylotranscriptomic insights into Asteraceae diversity, polyploidy, and morphological innovation. *J. Integr. Plant Biol.* **63**, 1273–1293 (2021).
11. Marçais, G. & Kingsford, C. A fast, lock-free approach for efficient parallel counting of occurrences of k-mers. *Bioinformatics* **27**, 764–770 (2011).
12. Vurture, G. W. *et al.* GenomeScope: Fast reference-free genome profiling from short reads. *Bioinformatics* **33**, 2202–2204 (2017).
13. Reyes-Chin-Wo, S. *et al.* Genome assembly with in vitro proximity ligation data and whole-genome triplication in lettuce. *Nat. Commun.* **8**, 14953 (2017).
14. Dudchenko, O. *et al. De novo* assembly of the *Aedes aegypti* genome using Hi-C yields chromosome-length scaffolds. *Science* **356**, 92–95 (2017).
15. Chin, C. S. *et al.* Phased diploid genome assembly with single-molecule real-time sequencing. *Nat. Methods* **13**, 1050–1054 (2016).
16. Walker, B. J. *et al.* Pilon: An integrated tool for comprehensive microbial variant detection and genome assembly improvement. *PLoS One* **9**, e112963 (2014).
17. Marçais, G. *et al.* MUMmer4: A fast and versatile genome alignment system. *PLoS Comput. Biol.***14**, e1005944 (2018).
18. Durand, N. C. *et al.* Juicer provides a one-click system for analyzing loop-resolution Hi-C experiments. *Cell Syst.* **3**, 95–98 (2016).
19. Jin, J. J. *et al.* GetOrganelle: A fast and versatile toolkit for accurate de novo assembly of organelle genomes. *Genome Biol.* **21**, 241 (2020).
20. Tillich, M. *et al.* GeSeq - Versatile and accurate annotation of organelle genomes. *Nucleic*

Acids Res. **45**, W6–W11 (2017).
21. Greiner, S., Lehwark, P. & Bock, R. OrganellarGenomeDRAW (OGDRAW) version 1.3.1: Expanded toolkit for the graphical visualization of organellar genomes. *Nucleic Acids Res.* **47**, W59–W64 (2019).
22. Campbell, M. S. *et al.* MAKER-P: A Tool kit for the rapid creation, management, and quality control of plant genome annotations. *Plant Physiol.* **164**, 513–524 (2014).
23. Grabherr, M. G. *et al.* Full-length transcriptome assembly from RNA-Seq data without a reference genome. *Nat. Biotechnol.* **29**, 644–652 (2011).
24. Jones, P. *et al.* InterProScan 5: Genome-scale protein function classification. *Bioinformatics* **30**, 1236–1240 (2014).
25. Bru, C. *et al.* The ProDom database of protein domain families: More emphasis on 3D. *Nucleic Acids Res.* **33**, D212-215 (2005).
26. Attwood, T. K. The PRINTS database: a resource for identification of protein families. *Brief. Bioinform.* **3**, 252–263 (2002).
27. Finn, R. D. *et al.* Pfam: The protein families database. *Nucleic Acids Res.* **42**, D222–D230 (2014).
28. Letunic, I. & Bork, P. 20 years of the SMART protein domain annotation resource. *Nucleic Acids Res.* **46**, D493–D496 (2018).
29. Thomas, P. D. *et al.* PANTHER: A library of protein families and subfamilies indexed by function. *Genome Res.* **13**, 2129–2141 (2003).
30. Hulo, N. *et al.* The PROSITE database. *Nucleic Acids Res.* **34**, D227-230 (2006).
31. Bateman, A. *et al.* UniProt: A hub for protein information. *Nucleic Acids Res.* **43**, D204–D212 (2015).
32. Burge, S. W. *et al.* Rfam 11.0: 10 years of RNA families. *Nucleic Acids Res.* **41**, D226-232 (2013).
33. Li, H. & Durbin, R. Fast and accurate short read alignment with Burrows-Wheeler transform. *Bioinformatics* **25**, 1754–1760 (2009).
34. Friedländer, M. R., MacKowiak, S. D., Li, N., Chen, W. & Rajewsky, N. MiRDeep2 accurately identifies known and hundreds of novel microRNA genes in seven animal clades. *Nucleic Acids Res.* **40**, 37–52 (2012).
35. Han, Y. & Wessler, S. R. MITE-Hunter: A program for discovering miniature inverted-repeat transposable elements from genomic sequences. *Nucleic Acids Res.* **38**, e199 (2010).
36. Ou, S. & Jiang, N. LTR_retriever: A highly accurate and sensitive program for identification of long terminal repeat retrotransposons. *Plant Physiol.* **176**, 1410–1422 (2018).
37. Xu, Z. & Wang, H. LTR-FINDER: An efficient tool for the prediction of full-length LTR retrotransposons. *Nucleic Acids Res.* **35**, W265--W268 (2007).
38. Ellinghaus, D., Kurtz, S. & Willhoeft, U. LTRharvest, an efficient and flexible software for de novo detection of LTR retrotransposons. *BMC Bioinformatics* **9**, 18 (2008).
39. Gordon, D. *et al.* Long-read sequence assembly of the gorilla genome. *Science* **352**, aae0344 (2016).
40. Li, H. *et al.* The Sequence Alignment/Map format and SAMtools. *Bioinformatics* **25**, 2078–2079 (2009).
41. Bickhart, D. M. *et al.* Single-molecule sequencing and chromatin conformation capture enable de novo reference assembly of the domestic goat genome. *Nat. Genet.* **49**, 643–650 (2017).

42. Kent, W. J. BLAT —The BLAST -Like Alignment Tool . *Genome Res.* **12**, 656–664 (2002).
43. Li, H. Minimap2: Pairwise alignment for nucleotide sequences. *Bioinformatics* **34**, 3094–3100 (2018).
44. Ou, S., Chen, J. & Jiang, N. Assessing genome assembly quality using the LTR Assembly Index (LAI). *Nucleic Acids Res.* **46**, e126 (2018).
45. Porebski, S., Bailey, L. G. & Baum, B. R. Modification of a CTAB DNA extraction protocol for plants containing high polysaccharide and polyphenol components. *Plant Mol. Biol. Rep.* **15**, 8–15 (1997).
46. Koren, S. *et al.* Canu: Scalable and accurate long-read assembly via adaptive κ-mer weighting and repeat separation. *Genome Res.* **27**, 722–736 (2017).
47. Ruan, J. & Li, H. Fast and accurate long-read assembly with wtdbg2. *Nat. Methods* **17**, 155–158 (2020).
48. Emms, D. M. & Kelly, S. OrthoFinder: solving fundamental biases in whole genome comparisons dramatically improves orthogroup inference accuracy. *Genome Biol.* **16**, 157 (2015).
49. Edgar, R. C. MUSCLE: Multiple sequence alignment with high accuracy and high throughput. *Nucleic Acids Res.* **32**, 1792–1797 (2004).
50. Capella-Gutiérrez, S., Silla-Martínez, J. M. & Gabaldón, T. trimAl: A tool for automated alignment trimming in large-scale phylogenetic analyses. *Bioinformatics* **25**, 1972–1973 (2009).
51. Stamatakis, A. RAxML version 8: A tool for phylogenetic analysis and post-analysis of large phylogenies. *Bioinformatics* **30**, 1312–1313 (2014).
52. Mirarab, S. *et al.* ASTRAL: Genome-scale coalescent-based species tree estimation. *Bioinformatics* **30**, i541-548 (2014).
53. Yang, Z. PAML 4: Phylogenetic analysis by maximum likelihood. *Mol. Biol. Evol.* **24**, 1586–1591 (2007).
54. Sanderson, M. J. r8s: Inferring absolute rates of molecular evolution and divergence times in the absence of a molecular clock. *Bioinformatics* **19**, 301–302 (2003).
55. Kumar, S., Stecher, G., Suleski, M. & Hedges, S. B. TimeTree: A resource for timelines, timetrees, and divergence times. *Mol. Biol. Evol.* **34**, 1812–1819 (2017).
56. Wang, Y. *et al.* MCScanX: A toolkit for detection and evolutionary analysis of gene synteny and collinearity. *Nucleic Acids Res.* **40**, e49 (2012).
57. Badouin, H. *et al.* The sunflower genome provides insights into oil metabolism, flowering and Asterid evolution. *Nature* **546**, 148–152 (2017).
58. Jiao, Y. *et al.* Ancestral polyploidy in seed plants and angiosperms. *Nature* **473**, 97–100 (2011).
59. Pelloux, J., Rustérucci, C. & Mellerowicz, E. J. New insights into pectin methylesterase structure and function. *Trends Plant Sci.* 12, 267–277 (2007).
60. Vitales, D., Fernández, P., Garnatje, T. & Garcia, S. Progress in the study of genome size evolution in Asteraceae: Analysis of the last update. *Database* **2019**, (2019).
61. Staton, S. E. & Burke, J. M. Evolutionary transitions in the Asteraceae coincide with marked shifts in transposable element abundance. *BMC Genom.* **16**, 623 (2015).
62. Bourque, G. *et al.* Ten things you should know about transposable elements. *Genome Biol.* **19**, 199 (2018).

63. Airoldi, C. A. *et al.* The arabidopsis BET bromodomain factor GTE4 is involved in maintenance of the mitotic cell cycle during plant development. *Plant Physiol.* **152**, 1320–1334 (2010).
64. Emms, D. M. & Kelly, S. OrthoFinder: phylogenetic orthology inference for comparative genomics. *Genome Biol.* **20**, 238 (2019).
65. De Bie, T., Cristianini, N., Demuth, J. P. & Hahn, M. W. CAFE: A computational tool for the study of gene family evolution. *Bioinformatics* **22**, 1269–1271 (2006).
66. Khalturin, K., Hemmrich, G., Fraune, S., Augustin, R. & Bosch, T. C. G. More than just orphans: are taxonomically-restricted genes important in evolution? *Trends Genet.* **25**, 404–413 (2009).
67. Stracke, R., Werber, M. & Weisshaar, B. The *R2R3-MYB* gene family in *Arabidopsis thaliana*. *Curr. Opin. Plant Biol.* **4**, 447–456 (2001).
68. Lichman, B. R. The scaffold-forming steps of plant alkaloid biosynthesis. *Nat. Prod. Rep.* **38**, 103–129 (2021).
69. Chellamuthu, V. R. *et al.* A widespread glutamine-sensing mechanism in the plant kingdom. *Cell* **159**, 1188–1199 (2014).
70. Chen, Y. M. *et al.* The PII signal transduction protein of *Arabidopsis thaliana* forms an arginine-regulated complex with plastid N-acetyl glutamate kinase. *J. Biol. Chem.* **281**, 24084 (2006).
71. Fokina, O., Chellamuthu, V. R., Forchhammer, K. & Zeth, K. Mechanism of 2-oxoglutarate signaling by the *Synechococcus elongatus* P II signal transduction protein. *Proc. Natl. Acad. Sci. U. S. A.* **107**, 19760–19765 (2010).
72. Bourrellier, A. B. F. *et al.* Chloroplast acetyl-CoA carboxylase activity is 2-oxoglutarate-regulated by interaction of PII with the biotin carboxyl carrier subunit. *Proc. Natl. Acad. Sci. U. S. A.* **107**, 502–507 (2010).
73. Hsieh, M. H., Lam, H. M., Van De Loo, F. J. & Coruzzi, G. A PII-like protein in Arabidopsis: Putative role in nitrogen sensing. *Proc. Natl. Acad. Sci. U. S. A.* **95**, 13965–13970 (1998).
74. Ferrario-Méry, S., Besin, E., Pichon, O., Meyer, C. & Hodges, M. The regulatory PII protein controls arginine biosynthesis in Arabidopsis. *FEBS Lett.* **580**, 2015–2020 (2006).
75. Baud, S. *et al.* PII is induced by WRINKLED1 and fine-tunes fatty acid composition in seeds of Arabidopsis thaliana. *Plant J.* **64**, 291–303 (2010).
76. Beez, S., Fokina, O., Herrmann, C. & Forchhammer, K. N-acetyl-l-glutamate kinase (NAGK) from oxygenic phototrophs: PII signal transduction across domains of life reveals novel insights in NAGK control. *J. Mol. Biol.* **389**, 748–758 (2009).
77. Selim, K. A., Ermilova, E. & Forchhammer, K. From cyanobacteria to Archaeplastida: new evolutionary insights into PII signalling in the plant kingdom. *New Phytol.* **227**, 722–731 (2020).
78. Watzer, B. *et al.* The signal transduction protein PII controls ammonium, nitrate and urea uptake in cyanobacteria. *Front. Microbiol.* **10**, 1428 (2019).
79. Ferrario-Méry, S. *et al.* Physiological characterisation of Arabidopsis mutants affected in the expression of the putative regulatory protein PII. *Planta* **223**, 28–39 (2005).
80. Ferrario-Méry, S., Meyer, C. & Hodges, M. Chloroplast nitrite uptake is enhanced in Arabidopsis PII mutants. *FEBS Lett.* **582**, 1061–1066 (2008).
81. Llácer, J. L. *et al.* The crystal structure of the complex of PII and acetylglutamate kinase

reveals how PII controls the storage of nitrogen as arginine. *Proc. Natl. Acad. Sci. U. S. A.* **104**, 17644–17649 (2007).
82. Mizuno, Y., Moorhead, G. B. G. & Ng, K. K. S. Structural basis for the regulation of N-acetylglutamate kinase by PII in *Arabidopsis thaliana*. *J. Biol. Chem.* **282**, 35733–35740 (2007).
83. Krapp, A. *et al.* Nitrate transport and signalling in Arabidopsis. *J. Exp. Bot.* **65**, 789–798 (2014).
84. Lezhneva, L. *et al.* The Arabidopsis nitrate transporter NRT2.5 plays a role in nitrate acquisition and remobilization in nitrogen-starved plants. *Plant J.* **80**, 230–241 (2014).
85. Chopin, F. *et al.* The Arabidopsis ATNRT2.7 nitrate transporter controls nitrate content in seeds. *Plant Cell* **19**, 1590–1602 (2007).
86. Ewald, R., Kolukisaoglu, Ü., Bauwe, U., Mikkat, S. & Bauwe, H. Mitochondrial protein lipoylation does not exclusively depend on the mtKAS pathway of *de novo* fatty acid synthesis in arabidopsis. *Plant Physiol.* **145**, 41–48 (2007).
87. Yao, K. *et al.* Expression of the Arabidopsis ADS1 gene in *Brassica juncea* results in a decreased level of total saturated fatty acids. *Plant Biotechnol. J.* **1**, 221–229 (2003).
