# Supplementary material for: Comparative genomics reveals a unique nitrogen-carbon balance system in Asteraceae
Source: Nat Commun. 2023 Jul 20;14:4334. doi: 10.1038/s41467-023-40002-9 (PMC10359422; doi:10.1038/s41467-023-40002-9)
Supplement: Supplementary file 3 — Description of Additional Supplementary Files [file 41467_2023_40002_MOESM3_ESM.pdf]

Description of Additional Supplementary Files

File Name: Supplementary Data 1
Description: Statistics of the enriched gene ontology (GO) terms based on the retention genes in the triplicated genomic regions of different species

File Name: Supplementary Data 2
Description: Statistics of the preferential retention genes in the triplicated genomic regions of Lactuca sativa var. angustana

File Name: Supplementary Data 3
Description: Statistics of the preferential retention genes in the triplicated genomic regions of Cynara

File Name: Supplementary Data 4
Description: Statistics of the preferential retention genes in the triplicated genomic regions of Helianthus annuus

File Name: Supplementary Data 5
Description: Statistics of the preferential retention genes in the triplicated genomic regions of Mikania

File Name: Supplementary Data 6
Description: Statistics of the LTR retrotransposons identified in the surveied genomes

File Name: Supplementary Data 7
Description: Statistics of the enriched InterPro entries associated with repetitive DNA elements in the Asteraceae species

File Name: Supplementary Data 8
Description: Prediction of genes inserted with repeat sequences in the Asteraceae family

File Name: Supplementary Data 9
Description: Prediction of transcription factor binding site (TFBS) introducted by repeat sequences in the Asteraceae family

File Name: Supplementary Data 10
Description: Statistics of the tanscription factor binding site (TFBS) introducted by repeat sequences with top ten occurrence

File Name: Supplementary Data 11
Description: Statistics of the enriched Interpro entries in Asteraceae

File Name: Supplementary Data 12
Description: Statistics of lineage-specific orthogroups in Asteraceae

File Name: Supplementary Data 13
Description: The lineage-specific genes of Asteraceae family related to stress-related genes

File Name: Supplementary Data 14

Description: Distribution of PII gene in all the species in OneKP project

File Name: Supplementary Data 15
Description: Summary of the primer sequences used in this study
