# Supplementary material for: Comparative genomics reveals a unique nitrogen-carbon balance system in Asteraceae
Source: Nat Commun. 2023 Jul 20;14:4334. doi: 10.1038/s41467-023-40002-9 (PMC10359422; doi:10.1038/s41467-023-40002-9)
Supplement: Supplementary file 19 — Reporting Summary [file 41467_2023_40002_MOESM19_ESM.pdf]

nature portfolio

Corresponding author(s): Xiaozeng Yang
Last updated by author(s): Jun 19, 2023

Reporting Summary

Nature Portfolio wishes to improve the reproducibility of the work that we publish. This form provides structure for consistency and transparency in reporting. For further information on Nature Portfolio policies, see our Editorial Policies and the Editorial Policy Checklist.

Statistics

For all statistical analyses, confirm that the following items are present in the figure legend, table legend, main text, or Methods section.

n/a	Confirmed	
☐	☒	The exact sample size (*n*) for each experimental group/condition, given as a discrete number and unit of measurement
☐	☒	A statement on whether measurements were taken from distinct samples or whether the same sample was measured repeatedly
☐	☒	The statistical test(s) used AND whether they are one- or two-sided<br>*Only common tests should be described solely by name; describe more complex techniques in the Methods section.*
☐	☒	A description of all covariates tested
☐	☒	A description of any assumptions or corrections, such as tests of normality and adjustment for multiple comparisons
☐	☒	A full description of the statistical parameters including central tendency (e.g. means) or other basic estimates (e.g. regression coefficient) AND variation (e.g. standard deviation) or associated estimates of uncertainty (e.g. confidence intervals)
☐	☒	For null hypothesis testing, the test statistic (e.g. *F*, *t*, *r*) with confidence intervals, effect sizes, degrees of freedom and *P* value noted<br>*Give P values as exact values whenever suitable.*
☐	☒	For Bayesian analysis, information on the choice of priors and Markov chain Monte Carlo settings
☐	☒	For hierarchical and complex designs, identification of the appropriate level for tests and full reporting of outcomes
☒	☐	Estimates of effect sizes (e.g. Cohen's *d*, Pearson's *r*), indicating how they were calculated

Our web collection on statistics for biologists contains articles on many of the points above.

Software and code

Policy information about availability of computer code

Data collection	NO software was used for the data collection.
Data analysis	3D-DNA (v201008)<br>Arrow v2.2.3<br>Augustus v3.0.3<br>BioNano Solve v3.0.1<br>BLASTp v2.9.0<br>BWA 0.7.12-r1039<br>CANU v1.7.1<br>cd-hit v4.6<br>Falcon v0.4<br>FastTree 2 v2.1.10<br>genomescope v1.0.0<br>Hisat2 v2.1.2<br>Infernal 1.1.12<br>InterProScan v5.24<br>IrysView v2.5.1<br>Juicer v1.5<br>LTR_Finder v1.07

LTR_retriever v2.9.0
LTRharvest (v1.5.9)
MAKER-P (v2.31.10)
MITE-Hunter (v1.0)
MUMmer v4.0.0rc
MUSCLE v3.8.31
trimAL v1.2
PASA (v2.4.1)
Pilon v1.2
R 3.5.0
RepeatMasker v4.0.6

SAMtools v1.4
StringTie 1.3.4
tRNAscan-SE v2.0.0
SigmaPlot v10.0
RNAmmer v1.2
cmscan (v1.1.4)
WTDBG2 (v2.5)
RAxML v.7.7.8
ASTRAL v5.5.1
miRdeep v0.1.3
PAML (v4.10.0)
CAFÉ v4.2.1
MCSCAN v1.3.6
EDTA v2.0.0
Cytoscape v3.8.2
Minimap2 v2.18
Jellyfish v2.0
Genomescope
Trinity v2.8.2
SNAP v1.0
RepeatModeler v1.0.11
MAFFT v7.429
OrthoFinder v2.4.0
r8s v1.8.1

For manuscripts utilizing custom algorithms or software that are central to the research but not yet described in published literature, software must be made available to editors and reviewers. We strongly encourage code deposition in a community repository (e.g. GitHub). See the Nature Portfolio guidelines for submitting code & software for further information.

Data

Policy information about availability of data

All manuscripts must include a data availability statement. This statement should provide the following information, where applicable:

- Accession codes, unique identifiers, or web links for publicly available datasets
- A description of any restrictions on data availability
- For clinical datasets or third party data, please ensure that the statement adheres to our policy

The sequencing data used in this study, assembled chromosomes, unplaced scaffolds, and annotations have been deposited into the Genome Sequence Archive (GSA) and Genome Warehouse (GWH) database in the BIG Data Center (https://bigd.big.ac.cn/gsa/index.jsp) under accession code PRJCA007442. Annotated information on stem lettuce in detail can also be found in LettuceGDB (https://lettucegdb.com/). Additional files such as the customized repeat library, gene trees and phylogenetic trees have been uploaded to Zenodo (https://zenodo.org/record/8058114). Source data are provided with this paper.

Human research participants

Policy information about studies involving human research participants and Sex and Gender in Research.

Reporting on sex and gender	N/A
Population characteristics	N/A
Recruitment	N/A
Ethics oversight	N/A

Note that full information on the approval of the study protocol must also be provided in the manuscript.

Field-specific reporting

Please select the one below that is the best fit for your research. If you are not sure, read the appropriate sections before making your selection.

- [x] Life sciences
- [ ] Behavioural & social sciences
- [ ] Ecological, evolutionary & environmental sciences

For a reference copy of the document with all sections, see nature.com/documents/nr-reporting-summary-flat.pdf

Life sciences study design

All studies must disclose on these points even when the disclosure is negative.

Sample size	The genomes of two samples (lettuce and Scaevola taccada) were sequenced. The leaf, root, stem and flower tissues for the two species for transcriptome sequencing in three biological replicates. For the comparative genomic anlaysis, a total of 29 representative plant species were selected.
Data exclusions	For the Illunima sequencing reads, we removed the adapter sequences and filtered out the low-quality reads.<br>For the genome assembly, we searched for the bacterial database to rule out the possible contamination. Additionally, the sequence from the organellar genomes were also excluded in the contigs.
Replication	Replications were used in the plant transformation, detection of metabolites, RNA-seq and RT-qPCR. Moreover, various bootstraps replicates were used in the phylogenetic analysis.
Randomization	No randomization were involved in our study.
Blinding	No experiment involved in the blinding were carried out.

Reporting for specific materials, systems and methods

We require information from authors about some types of materials, experimental systems and methods used in many studies. Here, indicate whether each material, system or method listed is relevant to your study. If you are not sure if a list item applies to your research, read the appropriate section before selecting a response.

Materials & experimental systems

n/a	Involved in the study	
[ ]	[x]	Antibodies
[x]	[ ]	Eukaryotic cell lines
[x]	[ ]	Palaeontology and archaeology
[x]	[ ]	Animals and other organisms
[x]	[ ]	Clinical data
[x]	[ ]	Dual use research of concern

Methods

n/a	Involved in the study	
[x]	[ ]	ChIP-seq
[ ]	[x]	Flow cytometry
[x]	[ ]	MRI-based neuroimaging

Antibodies

Antibodies used	HRP-conjugated GST-tag, Mouse mAb (Yeasen, #30903ES10)<br>MBP-tag, Rabbit pAb (Yeasen, #31201ES20)<br>Goat Anti-Rabbit Mouse lgG-HRP (Abmart,#M21003S)
Validation	All primary antibodies used in this study were commercially purchased and validation was performed by the individual companies. Validation data for the specific application is present on the data sheets provided by the company websites.

Flow Cytometry

Plots

Confirm that:

☒ The axis labels state the marker and fluorochrome used (e.g. CD4-FITC).

☒ The axis scales are clearly visible. Include numbers along axes only for bottom left plot of group (a 'group' is an analysis of identical markers).

☒ All plots are contour plots with outliers or pseudocolor plots.

☒ A numerical value for number of cells or percentage (with statistics) is provided.

Methodology

Sample preparation	Tender leaves were collected from the sequenced Sc. taccada plant and analysed using a flow cytometer. Populus trichocarpa (2n=2x=38) and tomato (Solanum lycopersicum) (2n=2x=24) samples were analysed to serve as the genome size reference.
Instrument	CyFlow Space flow cytometer (Partec, Germany), equipped with a UV-LED source (with emission at 365nm) and blue solid-state laser (λ = 455 nm)
Software	The data were analysed using Flomax2.8 (Sysmex Partec, France) , and the coefficient variation < 5%.
Cell population abundance	Over 5,000 nuclei per sample were collected and detected.
Gating strategy	Filter-625/26 was used in gating. The FL3-H/SSC-H gate method was used to eliminate the debris and cell fragments.

☐ Tick this box to confirm that a figure exemplifying the gating strategy is provided in the Supplementary Information.
